# Transformative biomechanics and mechanobiology breakthroughs shaping the future of health and medicine

**DOI:** 10.1016/j.xinn.2026.101307

**Published:** 2026-02-06

**Authors:** Long Li, Jing Ji, He Ren, Lilan Gao, Xiaona Li, Ning Li, Songbai Zhang, Kai Tang, Zedong Li, Weiyan Ren, Qing-ping Yao, Kai Huang, He Gong, Yingfeng Shao, Xianglong Lin, Xin Wang, Xiuqing Qian, Jie Song, Yiran Jiang, Hui Chen, Bo Che, Dongyuan Lü, Yu Du, Fan Feng, Yanli Liu, Yan Li, Meiying Luo, Ruotian Du, Cunyu Zhang, Guanshuo Hu, Yufei Ma, Shutong Wang, Rui Yang, Fang Pu, Bingjie Xiang, Ming Zhang, Xinghua Shi, Lizhen Wang, Bo Li, Damir Kračun, Qian Chen, Ahmed Elsheikh, Zi-Jun Liu, Baoyu Liu, Chuanrong Zhao, Yonggang Lü, Zhu Zeng, Zhiyong Li, Yiyao Liu, Guixue Wang, Wenchang Tan, Chunqiu Zhang, Min Zhang, Jizhong Lou, Youhua Tan, Linhong Deng, Mian Long, Ying-Xin Qi, Weiyi Chen, Feng Xu, Yubo Fan, Fan Song

**Affiliations:** 1 State Key Laboratory of Nonlinear Mechanics and Beijing Key Laboratory of Engineered Construction and Mechanobiology, Institute of Mechanics, Chinese Academy of Sciences, Beijing 100190, China; 2 Center of Materials Science and Optoelectronics Engineering, University of Chinese Academy of Sciences, Beijing 100049, China; 3 Key Laboratory for Biomechanics and Mechanobiology of the Ministry of Education, School of Biological Science and Medical Engineering, Beihang University, Beijing 100083, China; 4 Tianjin Key Laboratory for Advanced Mechatronic System Design and Intelligent Control, National Demonstration Center for Experimental Mechanical and Electrical Engineering Education, School of Mechanical Engineering, Tianjin University of Technology, Tianjin 300384, China; 5 Institute of Biomedical Engineering, Taiyuan University of Technology, Taiyuan 030024, China; 6 Center for Biomechanics and Bioengineering, Beijing Key Laboratory of Engineered Construction and Mechanobiology and Key Laboratory of Microgravity (National Microgravity Laboratory), Institute of Mechanics, Chinese Academy of Sciences, Beijing 100190, China; 7 State Key Laboratory of Oral & Maxillofacial Reconstruction and Regeneration, National Clinical Research Center for Oral Diseases, Shaanxi International Joint Research Center for Oral Diseases, Department of General Dentistry and Emergency, School of Stomatology, Fourth Military Medical University, Xi’an 710032, China; 8 The Hong Kong Polytechnic University Shenzhen Research Institute, Shenzhen 518057, China; 9 Department of Biomedical Engineering, The Hong Kong Polytechnic University, Hong Kong 999077, China; 10 The Key Laboratory of Biomedical Information Engineering of the Ministry of Education, School of Life Science and Technology, Xi’an Jiaotong University, Xi’an 710049, China; 11 Bioinspired Engineering and Biomechanics Center (BEBC), School of Life Science and Technology, Xi’an Jiaotong University, Xi’an 710049, China; 12 School of Engineering Medicine, Beihang University, Beijing 100083, China; 13 Institute of Mechanobiology & Medical Engineering, School of Life Sciences & Biotechnology, Shanghai Jiao Tong University, Shanghai 200240, China; 14 Innovation Center for Medical Engineering & Engineering Medicine, Hangzhou International Innovation Institute, Beihang University, Hangzhou 311115, China; 15 School of Biomedical Engineering, Capital Medical University, Beijing 100086, China; 16 State Key Laboratory of Epigenetic Regulation and Intervention, CAS Center for Excellence in Biomacromolecules, Institute of Biophysics, Chinese Academy of Sciences, Beijing 100101, China; 17 Institute of Biomedical Engineering and Health Sciences, Changzhou University, Changzhou 213164, China; 18 National Center for Nanoscience and Technology, Chinese Academy of Sciences, Beijing 100190, China; 19 School of Aerospace Engineering, Tsinghua University, Beijing 100084, China; 20 University Clinic Balgrist, Orthopaedic Biomechanics, Forchstrasse 340, 8008 Zurich, Switzerland; 21 Institute for Biomechanics, ETH Zurich, 8092 Zurich, Switzerland; 22 Laboratory of Molecular Biology and Nanomedicine, Department of Orthopaedics, Warren Alpert Medical School of Brown University, Providence, RI 02912, USA; 23 School of Engineering, University of Liverpool, Liverpool L69 7ZX, UK; 24 National Institute for Health Research (NIHR) Biomedical Research Centre, Moorfields Eye Hospital NHS Foundation Trust and UCL Institute of Ophthalmology, London EC1V 2PD, UK; 25 Department of Orthodontics, University of Washington, Washington, WA 98195-7446, USA; 26 Department of Pathology, University of Utah, Salt Lake City, UT 84111, USA; 27 Bioengineering College of Chongqing University, Chongqing 400044, China; 28 State Key Laboratory of New Textile Materials and Advanced Processing, Wuhan Textile University, Wuhan 430200, China; 29 Key Laboratory of Infectious Immune and Antibody Engineering of Guizhou Province, Engineering Research Center of Cellular Immunotherapy of Guizhou Province, Guizhou Medical University, Guiyang 561113, China; 30 Faculty of Sports Science, Ningbo University, Ningbo 315211, China; 31 Sichuan Provincial Key Laboratory for Human Disease Gene Study, Center for Medical Genetics, Sichuan Provincial People’s Hospital, School of Life Science and Technology, University of Electronic Science and Technology of China, Chengdu 610054, China; 32 TCM Regulating Metabolic Diseases Key Laboratory of Sichuan Province, Hospital of Chengdu University of Traditional Chinese Medicine, Chengdu 610072, China; 33 Key Laboratory for Biorheological Science and Technology of the Ministry of Education, State and Local Joint Engineering Laboratory for Vascular Implants, Bioengineering College of Chongqing University, Chongqing 400030, China; 34 Department of Mechanics and Engineering Science, Peking University, Beijing 100871, China; 35 Shenzhen Graduate School, Peking University, Shenzhen 518055, China; 36 College of Life Sciences, University of Chinese Academy of Sciences, Beijing 100049, China; 37 School of Engineering Sciences, University of Chinese Academy of Sciences, Beijing 100049, China

**Keywords:** biomechanics, mechanobiology, mechanodiagnosis, mechanotherapy, rehabilitation engineering

## Abstract

The mechanical environments endured by the human body profoundly influence life activities across different scales, from single molecules to complicated systems. Gaining insight into the mechanical factors and their biological implications is crucial for deciphering physiological and pathological processes and advancing innovations in drug development and therapeutic approaches for various diseases. Recently, we have witnessed rapid advances in biomechanics and mechanobiology, which, however, are not fully recognized by the clinical community and effectively integrated into medical decision-making, highlighting a translational gap between mechano-based discovery and therapeutic application. Here, we first provide a comprehensive review of research progress in biomechanics and mechanobiology, focusing on key areas such as the cardiovascular system, bone and joints, ocular tissues, liver, lung, the craniomandibular system, cancer, and immunology. We demonstrate how mechanical cues drive health and disease across biological levels, offering insights into complex physiological and pathological mechanisms. Further, we explore the diverse applications of biomechanics and mechanobiology in disease diagnosis, treatment, and rehabilitation. Mechanical insights fuel medical innovations through advanced diagnostic tools, novel therapies, and effective rehabilitation protocols, enhancing clinical outcomes. Looking ahead, we outline future directions of biomechanics and mechanobiology, emphasizing interdisciplinary integration, artificial intelligence, model development, and extreme environments, which hold the promise to deepen scientific understanding and propel technological innovations. This review highlights the transformative potential of biomechanics and mechanobiology in driving scientific and clinical advancements and helps bridge the long-standing gap between biomechanical research and clinical practice.

## Public summary

• How mechanical cues drive health and disease across biological levels is comprehensively elucidated.
• Therapeutic applications of biomechanics and mechanobiology are extensively explored.
• Future directions of biomechanics and mechanobiology are outlined to steer next-generation studies.
• Our work helps bridge the translational gap between biomechanical research and clinical practice.

## Graphical abstract

**Figure:**
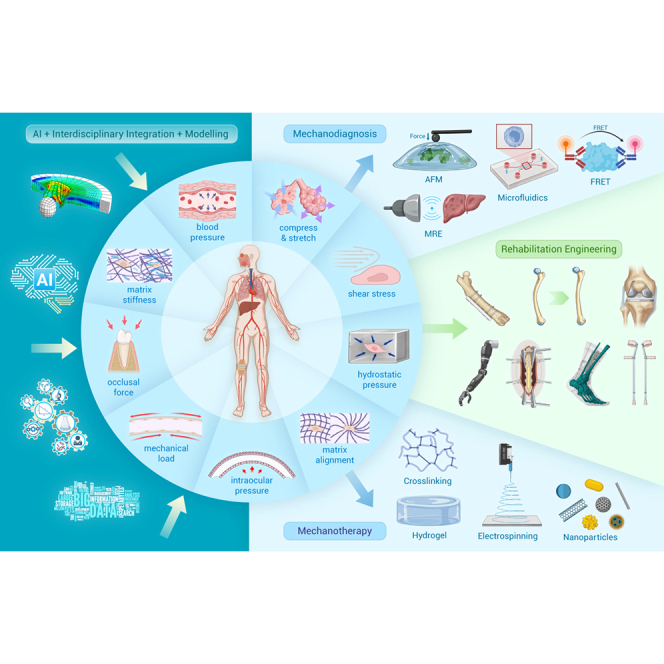

## Introduction

The human body operates within complex mechanical environments. Mechanical factors play a crucial role in shaping life, influencing biological processes across molecular, cellular, tissue, organ, and systemic levels.^1^^,^^2^^,^^3^^,^^4^^,^^5^^,^^6^ For example, the contraction and relaxation of the heart pump blood throughout the circulatory system, delivering oxygen and nutrients while removing metabolic waste products, including carbon dioxide and urea. Abnormalities in these mechanical activities are closely associated with the onset and progression of cardiovascular diseases, including heart failure and atherosclerosis. Similarly, the mechanical microenvironment surrounding cells has been recognized as a critical factor for various cellular processes, including growth, proliferation, differentiation, senescence, apoptosis, and even tumorigenesis. Targeting these mechanical microenvironments has offered novel therapeutic strategies for many diseases. Studying mechanical factors and their effects is therefore pivotal for uncovering the laws of life, elucidating disease mechanisms, and advancing innovations in drug development and therapeutic approaches.

Biomechanics, established as an independent scientific discipline in the 1960s, exemplifies an interdisciplinary approach by incorporating principles from biology, medicine, chemistry, and materials science. The term “biomechanics” originates from the Ancient Greek: βίος (life) and μηχανική (mechanics). By applying fundamental principles of mechanics to biological systems, biomechanics investigates the deformation and motion of living organisms to elucidate biological mechanisms and address scientific challenges in health and life sciences. This discipline can be broadly categorized into biofluid mechanics, biosolid mechanics, and sports biomechanics, based on the classical mechanics classifications and the unique characteristics of biological systems. Biofluid mechanics explores the flow behavior of bodily fluids and gases within living organisms, employing principles of fluid mechanics and aerodynamics. Biosolid mechanics focuses on the mechanical properties of solid biological tissues such as bones, muscles, and blood vessels, utilizing theories of material mechanics, elasticity, plasticity, and fracture mechanics. Sports biomechanics analyzes the movements of living organisms, applying the principles of statics, kinematics, and dynamics.

Pioneering research has systematically characterized the mechanical properties and behaviors of cells, tissues (e.g., muscles, bones, skin, and teeth), and organs (e.g., brain, lungs, liver, and eyes); fluid and gas dynamics; as well as sports injury rehabilitation. These studies have developed experimental methods for determining biomechanical properties, established constitutive relationships for biological materials, and uncovered fundamental laws governing the fluid flow within organisms and the mechanical motion of biological systems. These efforts have provided us with significant insights and enlightening information regarding how mechanical environments interact with living systems. A landmark contribution was made by Dr. Yuan-Cheng Fung, often regarded as the “father of biomechanics,” who explored the critical relationship between stress and growth of living organisms.^7^ Starting with the measurement of zero-stress states and the analysis of residual stress in biological tissues, Fung proposed the renowned “stress-growth law.”^8^ This theory has provided essential guidance applicable to many medical fields, including cardiovascular interventions, bone injury rehabilitation, and orthodontics. His work exemplifies the transformative shift in biomechanics from a discipline focused on applying mechanics to biology to one that seamlessly integrates mechanical principles with biological processes.

Advancements in science and technology have further propelled the evolution of biomechanics into the realm of mechanobiology. This emerging field investigates how mechanical environments (stimuli) influence health, disease, and injury at molecular and cellular levels and explores how organisms perceive and respond to mechanical signals. Mechanobiology seeks to elucidate the interplay between mechanical factors and biological processes, such as growth, remodeling, adaptive change, and repair. By bridging biomechanics with molecular biology, mechanobiology fosters the development of diagnostic and therapeutic innovations, thereby advancing both biomedical research and clinical applications.

Despite significant progress, a comprehensive literature review reveals that there remains a substantial gap in our understanding of the mechanical environments that underlie various physiological and pathological states. The clinical community has yet to fully recognize the diagnostic and therapeutic potential of mechanical cues, hindering the translation of biomechanical insights into medical practice. This review bridges this knowledge gap by providing a thorough and up-to-date overview of research progress in biomechanics and mechanobiology across critical areas such as cardiovascular systems, bone and joints, ocular tissues, liver, lung, the craniomandibular system, cancer, and immunology. The central thesis of this review is to establish a coherent and integrated narrative that spans fundamental biomechanics and mechanobiology, their biomedical applications, and future perspectives. More specifically, we begin by introducing a conserved set of mechanical variables—including stretch, compression, shear stress, hydrostatic pressure, and extracellular matrix (ECM) stiffness—under both physiological and pathological conditions across those critical areas. We then synthesize important progress in biomechanics, with particular focus on the mechanical properties and behaviors of cells, tissues, and organs, as well as the dynamics of fluid and gas. Within the mechanobiological framework, we further discuss advances in how mechanical cues regulate molecular, cellular, and tissue functions, emphasizing key mechanotransducer and mechanoeffectors—including integrins, Piezo channels, Yes-associated protein (YAP), transforming growth factor β (TGF-β), protein kinase B, Notch, and Wingless/Int (Wnt)—and their downstream signaling pathways. By elucidating how mechanical cues drive health and disease at multiple biological levels, we offer novel insights into complex physiological mechanisms and explore their practical applications in disease diagnosis, treatment, and rehabilitation, with representative translational cases included. Finally, we further highlight future directions, emphasizing interdisciplinary integration, artificial intelligence (AI), model development, and extreme environments, as well as emerging branches such as mechanogenomics and mechanoepigenetics. Existing reviews have examined biomechanics or mechanobiology. However, most focus on a single domain, and even those addressing both fields typically confine their scope to a specific biological context. By contrast, our review integrates biomechanics and mechanobiology across multiple systems and scales, providing a more comprehensive and holistic perspective than currently available in the literature. Our integrative approach distinguishes this review from existing literature in terms of comprehensiveness, depth, and foresight, making it a valuable resource for researchers, clinicians, and innovators dedicated to advancing health and medical sciences through the transformative power of biomechanics and mechanobiology.

## Biomechanical and mechanobiological research advancement

In this section, we summarize biomechanical and mechanobiological research advancements across multiple areas, including the cardiovascular system, bone and joints, ocular tissues, liver, lung, the craniomandibular system, cancer, and immunology. The mechanical cues and processes of mechanosensation and mechanotransduction are discussed in each system to elucidate how cells interpret physical cues and convert them into biochemical signals that drive physiological function and pathology. In particular, we list the key mechanotransduction pathways associated with Piezo1 and integrins in Tables S1 and S2 for each reviewed area to offer comparative insights and enhance clarity. While these eight areas form the primary focus of our discussion, it is important to note that the mechanical factors also exert a significant influence on biological processes in many other systems, such as brain, kidney, stomach, gastrointestinal tract, skin, and bladder. For example, mechanical stimuli have been shown to regulate skin growth and wound healing^9^ and to influence bladder compliance and urinary incontinence.^10^ Although these systems are not elaborated in detail here due to space constraints, these findings underscore the broad and fundamental relevance of biomechanics and mechanobiology across the human body.

### Cardiovascular system

**Figure 1:**
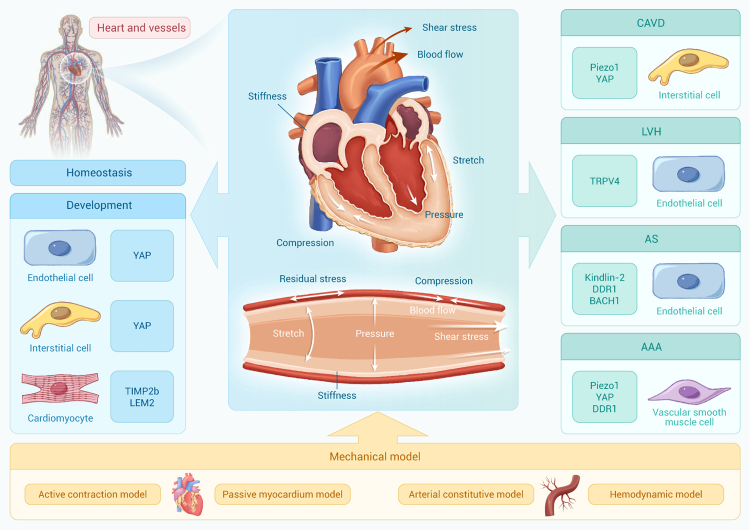
Biomechanics and mechanobiology in the cardiovascular system

The cardiovascular system consists of the heart, blood vessels, and blood (Figure 1). In the cardiovascular system, cardiac pumping generates forces that deliver blood to peripheral vessels.^11^^,^^12^^,^^13^^,^^14^ The contraction and relaxation of the heart promote blood flow to generate various mechanical forces, including cyclic stretch (rhythmic elongation and widening of the cardiovascular wall), compression (axial shortening due to external force), shear stress (parallel to lumen walls, caused by blood flow resistance), and hydrostatic pressure (perpendicular to lumen walls, resulting from fluid column pressure).^15^ Residual stress arises from intrinsic material heterogeneity within the tissue, which results from various factors such as tissue development, growth, remodeling, and osmotic swelling.^16^ Moreover, increased attention has recently been given to the mechanical microenvironment of the ECM^15^ and the viscoelasticity of cardiovascular tissue.^16^^,^^17^

These physiological and pathological mechanical stimuli are collectively referred to as “mechanical stresses.” Indeed, cells sense mechanical stresses via mechanosensors, triggering intracellular signaling networks—a process known as mechanotransduction. Mechanosensors and downstream mechanotransduction signaling pathways play important roles in the cardiovascular system, including tissue development, homeostasis maintenance, and disease progression.^15^^,^^18^^,^^19^^,^^20^ Abnormal mechanical stresses contribute to cardiovascular diseases, such as excessive stretch in hypertension, low and disturbed shear stress in atherosclerosis, and increased stiffness in calcification.^15^ Conducting research in biomechanics and mechanobiology is critical for understanding the underlying mechanisms of cardiovascular physiology and pathology. Several important reviews have addressed mechanotransduction processes in the cardiovascular system,^15^^,^^21^^,^^22^^,^^23^^,^^24^ providing insight into how mechanical signals influence cellular behavior, tissue function, and disease progression. Herein, we summarize the latest research, focusing on both biomechanical and mechanobiological perspectives of the heart and vessels, to discuss future directions in the cardiovascular system.

#### Biomechanical studies of the heart and vessels

Quantifying mechanical parameters such as morphology, material properties, and hemodynamics; reproducing the cardiovascular system *in vitro*; and analyzing both biological and pathological processes are the primary aims of cardiovascular biomechanics.^25^^,^^26^ Advances in experimental measurement, computational modeling, and data-driven techniques have markedly deepened our understanding of these fundamental aspects.

In the realm of experimental measurement, a broad spectrum of techniques spanning organ, tissue, and cellular scales has fundamentally advanced biomechanical studies of the heart and vessels. At the whole-organ level, cardiac magnetic resonance imaging (MRI) provides high-fidelity quantification of myocardial strain, wall motion, and volumetric changes throughout the cardiac cycle.^27^ Four-dimensional (4D) flow MRI has emerged as a powerful modality, enabling direct measurement of three-dimensional (3D) blood flow patterns, vorticity structures, and wall shear stress in major vessels.^28^ These measurements have been instrumental in elucidating hemodynamic abnormalities in congenital heart disease, aneurysms, and valvular disorders. At the tissue scale, biaxial tensile testing is widely employed to characterize nonlinear, anisotropic behavior of arterial walls, yielding essential insights into collagen fiber recruitment, elastin degradation, and residual stress. At even finer scales, techniques such as atomic force microscopy (AFM) and micropipette aspiration allow mechanical probing of individual vascular cells and ECM components.^29^ These cellular-level assays help to elucidate how the mechanical behavior of cells residing in the cardiovascular system changes in response to shear stress, cyclic stretch, and substrate stiffness.

Computational modeling has also made substantial contributions to advancing cardiovascular biomechanics. In the heart, electrophysiological activation induces cyclic contraction and relaxation of myocardial walls, which are typically reproduced by combining an active contraction model with a passive myocardium model.^30^ There are three principal approaches for constructing an active contraction mode: the active stress, the active strain, and the hybrid approach.^31^ A recent study integrated the active stress approach, based on the Hill model, with the active strain approach to develop a novel hybrid contraction model, which is more suitable for personalized cardiac modeling.^31^ Passive myocardium models not only simulate macroscale stress-strain behavior at the tissue level but also incorporate myofiber-collagen interaction.^32^ The current micro-model, based on micro-anatomically realistic finite element remodeling, can simulate the mechanical behavior of individual fibers and reproduce macroscale cardiac function.^32^ Furthermore, comprehensive models that incorporate both active muscle contraction and passive myocardial tissue can realistically simulate cardiac tissue structure, electrophysiology, and the mechanical actuation of the heart.^33^

The arterial wall consists of intima, media, and adventitia. The adventitia is mainly composed of fibroblasts and collagen fibers, which respond to mechanical stress in a nonlinear manner. The media contains an elastic fiber-rich sheet-like network known as lamellae, which responds to mechanical stress in a predominantly linear manner.^34^ Vascular smooth muscle cells (VSMCs), collagen, and elastic fibers are interspersed between the lamellae and collectively constitute the media. The intima, composed of a monolayer of endothelial cells (ECs), contributes little to the mechanical properties of the vessel wall, but it can be integrated with the media to form a two-layer constitutive model of the artery.^34^ Computational fluid dynamics (CFD) based on 3D imaging is a well-established approach for simulating arterial blood flow, but it is limited by its high computational cost.^35^ Current studies focus on combining 3D and one-dimensional (1D) models. Namely, the global arterial tree is represented by a 1D model, while the region of interest is modeled in 3D to reduce computational cost and ensure local accuracy.^35^

Numerous indices derived from mechanical models either show clinical potential or are already being applied to evaluate cardiovascular diseases. Increased left ventricular stiffness and elevated filling pressure are important clinical indicators used to evaluate left ventricular dysfunction.^36^ Hemodynamic models are used to analyze the development and progression of coronary stenosis,^37^ artery aneurysms,^38^ or branch lesions.^39^ Analysis of the biological structure of atherosclerotic plaques using biomechanical computation helps to understand their formation and risk of rupture in individual patients.^40^

The future of biomechanics depends on its integration with emerging technologies, making this convergence essential for advancing research and clinical application. For example, with the rapid development of AI, mechanical models have emerged as powerful tools to simulate *in vivo* conditions and predict underlying physiological mechanisms. In particular, AI- and machine-learning-driven CFD simulations of hemodynamics have offered a powerful solution to the challenges of complex operations and high computational cost inherent in traditional methods. The efficiency and reliability of these algorithms make them a valuable complement for the studies of cardiovascular biomechanics, hemodynamics, and treatment planning.^41^^,^^42^^,^^43^

#### Mechanobiological studies of the heart and vessels

The heart is the first functional organ of the developing vertebrate embryo that is continuously exposed to mechanical stresses throughout life. Initially, the mechanical microenvironment of the ECM, including stiffness, is necessary for cardiac morphogenesis and final maturation.^21^ The stiffening, which results from changes in ECM composition and contractile proteins expression, triggers the mechanical responses of cardiomyocyte progenitors and fibroblast progenitors.^21^ Blood flow applies both shear stress and pressure to promote heart development, involving cellular proliferation, migration, and differentiation.^44^ Wang et al. revealed that shear stress and hydrostatic pressure activate YAP, a crucial mechanosensitive effector shuttling between cytoplasm and nucleus, to promote heart valve formation.^45^ In addition, cardiac contraction and blood flow promote ECM remodeling in the developing zebrafish heart, a process that partly relies on tissue inhibitor of metalloproteinase 2b (TIMP2b), indicating interactions among different mechanical stresses.^46^

It has been widely accepted that mechanical stresses activate membrane mechanosensors such as integrins and Piezo and then provoke downstream signaling pathways to regulate heart development,^44^ whereas the transduction of mechanical signaling does not solely rely on membrane mechanosensors, as direct physical changes of cellular shape can transmit mechanical changes from the cytoskeleton to the nucleus.^44^ The nuclear lamina (composed of lamins A/C, B1, and B2) underlying the nuclear envelope (NE) is critical to maintain nuclear shape and normal cellular functions.^44^ In addition, the linker of nucleoskeleton and cytoskeleton (LINC) complex, together with its associated proteins—including SUN and nesprin family members—plays a crucial role in nuclear-cytoskeletal coupling.^47^ Proteins of the inner nuclear membrane, such as Lamina-associated peptide 2 (LAP2), Emerin, and LEM-domain-containing proteins (LEMDs), also contribute importantly to cardiac nuclear integrity and mechanotransduction.^47^ For example, LEMD2 has been shown to maintain nuclear integrity during heart development, particularly in stages when the NE remains immature.^48^

In the mature heart, cardiac contraction is initiated by electrical signals, causing cardiomyocytes to shorten and eject blood against a resistance known as afterload. During cardiac relaxation, ventricular filling stretches the cardiomyocytes, generating a preload that influences the force of the next contraction.^49^ Preload and afterload vary from beat to beat through rapid mechanical feedback mechanisms. These feedback processes are mediated by mechano-electric coupling (MEC), which influences cardiac electrical activity, and mechano-mechanical coupling (MMC), which modulates myocardial contraction.^49^ Mechanical feedback is essential for cardiac function, enabling rapid adaptation to acute physiological changes such as variations in blood pressure or heart rate.^50^ However, the effects of MEC and MMC on the developing heart remain poorly understood.^49^ In short, the heart is continuously exposed to various mechanical stresses arising from the ECM, blood flow, and cardiac contraction, all of which play key roles in regulating its development and functions.

Abnormal mechanical stresses, such as excessive pressure, disturbed flow, or matrix stiffening, are recognized as significant contributors to various forms of heart disease, including hypertrophy, fibrosis, and valvular pathologies. During heart development, atrioventricular valve delamination depends on normal blood flow. Abnormal hemodynamics, characterized by increased reversing flow and more rapid change in flow patterns, inhibits the mesenchymal-endothelial transition (MEndoT) of abluminal cells. This effect is partly mediated by repression of the nuclear factor of activated T cells (NFAT) signaling pathway, leading to delayed delamination and congenital valve hyperplasia.^51^ Hemodynamic forces continue to play a critical role in the mature heart, influencing processes such as valve maintenance, myocardial remodeling, and vascular homeostasis. For example, oscillatory shear stress induces calcific aortic valve disease (CAVD) by activating the Piezo1/YAP/glutaminase1 signaling pathway and promoting osteogenic differentiation of valvular interstitial cells (VICs).^52^ Chronic hypertension and myocardial infarction are common causes of altered hemodynamics, often leading to left ventricular hypertrophy (LVH).^53^

Pressure overload is a key mechanical contributor to heart disease and can ultimately lead to heart failure, particularly when compensatory hypertrophy becomes maladaptive. The cardiomyocyte is the main cell type affected by the pressure-overload-induced dysfunction. Accumulated evidence indicates that restoring cellular metabolism and alleviating oxidative stress may offer promising therapeutic strategies.^15^^,^^54^^,^^55^ The pathogenesis of cardiomyocyte dysfunction may also be influenced by intracellular crosstalk with cardiac fibroblasts and vascular mural cells, which modulate the ECM, paracrine signaling, and microvascular integrity.^56^ In ECs, mechanical pressure activates the transient receptor potential (TRP) vanilloid family member 4 (TRPV4) channel, impairing coronary angiogenesis and contributing to hypertrophic stress.^53^

ECM remodeling is also recognized as an early event of CAVD, wherein changed stiffness serves as a key factor influencing mechanotransduction. Increased ECM stiffness activates various mechanosensitive pathways, including Piezo1, Ras homolog family member A (RhoA)/ROCK, and YAP/TAZ, and promotes the osteogenic differentiation of VICs.^57^ When combined with shear stress and inflammation, ECM stiffness creates a positive feedback loop that drives the progression of CAVD.^57^ Moreover, cyclic mechanical stretch contributes to cardiac pathological processes by promoting the activation of cardiac fibroblasts, ultimately leading to cardiac fibrosis.^58^

Knowledge from mechanogenomics and mechanoepigenetics provides insights into how mechanical forces influence nuclear architecture, chromatin conformation, and gene expression, thereby yielding a deeper fundamental understanding of the molecular mechanisms by which mechanical cues regulate cellular function and fate. Recently, Horii et al. found that substrate stiffness can induce vestigial-like family member 3 (VGLL3) expression and trigger its translocation into the nucleus of cardiac myofibroblasts through the integrin β1-Rho-actin pathway, which in turn increases collagen expression and promotes cardiac fibrosis.^59^ In addition, Hu et al. investigated how the communications between extracellular stress and chromatin structure modulate cellular mechanical behaviors. Their findings reveal that histone H1.0 orchestrates a genome-wide reorganization of chromatin to facilitate transcriptional changes in cytoskeletal and ECM genes, thereby directly controlling cellular force generation and ECM deposition. Meanwhile, they found that depleting histone H1.0 prevents cardiac fibrosis.^60^

In developing vessels, progressive tissue stiffening is essential for vascular network formation, in part through mechanical forces that modulate juxtacrine interaction between ECs and fibroblasts.^61^ Cellular contractile forces synergize with ECM remodeling and play a major role in the stiffening process, which depends on YAP-mediated mechanotransduction in the fibroblasts.^61^ In developing vessels lacking an organized medial layer, lumen diameter expansion depends on EC proliferation, migration, and enlargement—processes that are highly sensitive to hemodynamic forces.^62^ Subsequently, circumferential stretch resulting from increased lumen diameter and blood pressure promotes the differentiation of mural cells into mature VSMCs.^62^

In mature vessels, increasing evidence reveals the critical role of hemodynamic shear stress in maintaining EC homeostasis. Mechanosensitive K^+^ and Ca^2+^ ion channels in ECs respond to shear stress by regulating nitric oxide (NO) production and promoting vasodilation.^22^ In addition to membrane receptors, the physical properties of EC plasma membranes change in response to the mechanical microenvironment, thereby activating intracellular signaling pathways. For example, ECs reduce membrane cholesterol—a key component that determines membrane mechanical properties—in order to adapt to shear stress, thereby activating mitochondrial oxidative phosphorylation.^63^

A classic example of how VSMCs respond to mechanical stress is the myogenic response, which refers to acute vascular vasoconstriction that occurs in response to elevated transmural pressure (reviewed by Davis et al.^64^). The myogenic response is an intrinsic property of VSMCs and occurs independently of ECs or the nervous system.^22^^,^^64^ Various mechanosensors are activated by stretch and trigger the downstream signaling pathways, with myosin phosphorylation playing a central role, in generating contractile force in VSMCs.^22^^,^^65^

Collectively, these studies underscore the vital role of mechanical stress in shaping vascular development, regulating endothelial and smooth muscle cell behavior, and maintaining vascular physiology. Furthermore, ECs and VSMCs employ distinct mechanisms to maintain homeostasis in response to mechanical perturbations. Notably, vascular mechanotransduction research has primarily focused on ECs and VSMCs, while other cell types—such as fibroblasts and immune cells—remain understudied and warrant further investigation.

It is well established that straight arteries experiencing laminar blood flow with high shear stress are protected against atherogenesis. In contrast, arterial branches and curvatures exposed to disturbed or low shear stress are prone to atherosclerosis.^66^ More specifically, under low-shear-stress conditions, early atherosclerotic lesions typically exhibit increased fibrous and fibrofatty tissue, which induces a continuous rise in plaque structural stress.^67^ As the plaque progresses, the resulting elevation in plaque structural stress consequently slows further fibrous accumulation and promotes necrotic core development, thereby rendering the plaque increasingly vulnerable.^67^ Additionally, mechanical wall stress induced by abnormal blood pressure has also been significantly correlated with plaque progression,^68^ providing evidence that aberrant mechanical cues exacerbate vascular diseases.

In ECs, in addition to previously described mechanotransduction pathways—including Krüppel-like factor 2 (KLF2), nuclear factor erythroid 2-related factor 2 (NRF2), YAP, Notch, and Wnt^69^—recent studies have identified novel flow-responsive molecules that contribute to atherogenesis, such as mitochondrial uncoupling protein 2 (UCP2)^70^ and mammalian sterile 20-like kinase 1 (MST1).^71^ These novel mechanoresponsive molecules provide potential pharmacological targets for atherosclerosis. A recent study found that endothelial enhancers, a type of *cis*-regulatory element, are sensitive to hemodynamic changes generated by disturbed flow and are involved in atherogenesis.^72^ These endothelial enhancers physically interact with the promoters of genes associated with vascular pathology, offering an epigenetic perspective on vascular lesions induced by abnormal mechanical stress.^72^

In VSMCs, the cytoskeleton has been shown to be essential for transducing mechanical cues—such as stretch, stiffness, and pressure—in various pathological processes.^73^ The most remarkable change of VSMCs during these processes is phenotype switching, which refers to the loss of their contractile function and transformation into proliferative, osteogenic, senescent, or other functional phenotypes.^73^ Recently, Pan et al. systemically demonstrated that phenotypically switched VSMCs within atherosclerotic plaque exhibit multiple similarities to tumor cells, including resistance to replicative senescence and cell death, high proliferative and invasive potential, and activation of cancer-related gene regulatory networks.^74^ Cytoskeleton-mediated mechanotransduction has been implicated in the pathogenesis of abdominal aortic aneurysms (AAAs).^75^ During AAAs, Netrin-1 increases the cytoskeletal stiffness in VSMCs by upregulating α-actinin2, which in turn activates Piezo1 and promotes vascular remodeling.^75^

The discovery of microRNA (miRNA), recognized by the 2023 Nobel Prize in Physiology or Medicine, has contributed to our understanding of how mechanical stress regulates cardiovascular homeostasis.^76^ It has been previously found that cyclic stretch modulates lamin A/C via miR-124-3p, leading to apoptosis of VSMCs under hypertensive conditions.^77^ In the vein grafts, cyclic stretch has been shown to promote neointimal hyperplasia through the inhibition of miR-33.^78^ Non-coding RNAs, including miRNAs, offer novel insights into how mechanical stresses contribute to vascular dysfunction.

Current mechanobiological studies mainly focus on the large vessels, while research on microvessels is still very limited. Hemodynamic forces remain essential for regulating microvascular remodeling, despite the smaller-lumen-diameter and low-flow-rate characteristics of microvessels.^79^ Mechanosensors such as Piezo1 and integrins are also functional in microvessels, allowing ECs to detect and respond to local hemodynamic changes. For example, blood flow regulates the activity of Piezo1 in capillaries of the central nervous system.^80^ Capillaries, as the smallest blood vessels, experience unique mechanical environments resulting from the squeezing of red blood cells (RBCs) through their narrow lumen,^80^ as well as from the formation of biomolecular condensates at liquid-liquid and liquid-solid interfaces.^81^ Capillary lesions—such as ventilator-induced lung injury,^82^ traumatic-brain-injury-induced blood-brain dysfunction,^83^ and glomerular injury caused by congenital anomalies of the kidney and urinary tract^84^–are strongly associated with mechanical stressors. However, the effects of these mechanical stresses on capillary physiology and pathology are poorly understood.

#### Future remarks

Building on the foundational and remarkable work of Dr. Yuan-Cheng Fung^85^ and Dr. Shu Chien,^86^ cardiovascular biomechanics remains one of the most actively researched and rapidly advancing fields within the broader landscape of mechanobiology. However, the intricate variability of hemodynamics in the cardiovascular system, along with the complex constitutive behavior of cardiovascular tissues, poses significant challenges to this field. For example, the intrinsic rhythmic contraction of the heart generates a unique and complex mechanical environment that differs markedly from those in other organ systems. Mechanical stresses vary considerably along the vasculature; hence, vascular cells in various regional circulations and in different vascular segments are exposed to different stimuli.

The development of biomechanical studies needs to constantly overcome the complexity of the mechanical environment and pursue flexible multiscale simulation. The future of biomechanics lies in its integration with emerging technologies, including AI, 3D printing, and immersive platforms such as augmented and virtual reality (AR and VR). In fact, in advanced clinical practice, AR and VR have already begun transforming fields, e.g., cardiovascular education and intervention planning, by offering interactive, real-scale anatomical models—paving the way for customized and more accurate procedures. Recently, mobile AR tools such as HybridCollab have enabled both local and remote teams to manipulate a shared 3D heart model during surgical planning sessions.^87^ Applying more efficient, precise, safe, and economical biomechanical techniques to clinical diagnosis and treatment is an eternal topic. From a mechanobiological perspective, although there are multitudinous studies for every type of mechanical force, the interactions between these forces or their hierarchical relationships are still unclear. In the future, the crosstalk between different cells or organs needs to be focused on. The VSMC phenotype has recently been found to have a commonality from a view of tumor cells, indicating that the current description of cytopathological mechanisms and phenomena may require revision. The endothelial enhancers and cancer-related gene regulatory networks in VSMC mentioned above both hint that deeper secrets may be hidden in the genes. Furthermore, mechanisms in the nucleus, especially the role of NE proteins in mechanotransduction, need more attention. In addition, multiple classes of mechanosensitive molecules—including integrins, cadherins, selectins, and cytoskeletal proteins—exhibit catch-bond behavior that modulates adhesion strength and signaling under load. Further in-depth investigation of molecular interaction and force-dependent mechanotransduction mechanisms in the cardiovascular system will help clarify how mechanical cues shape cellular decision-making, remodel vascular structures, and contribute to the initiation and progression of cardiovascular diseases. Despite considerable therapeutic potential, epigenetic therapies still have substantial limitations, particularly their broad spectrum of action, which can inadvertently affect multiple cell types and organs. Therefore, future therapeutic strategies will require addressing challenges of cell specificity and side effects.

### Bone and joints

**Figure 2:**
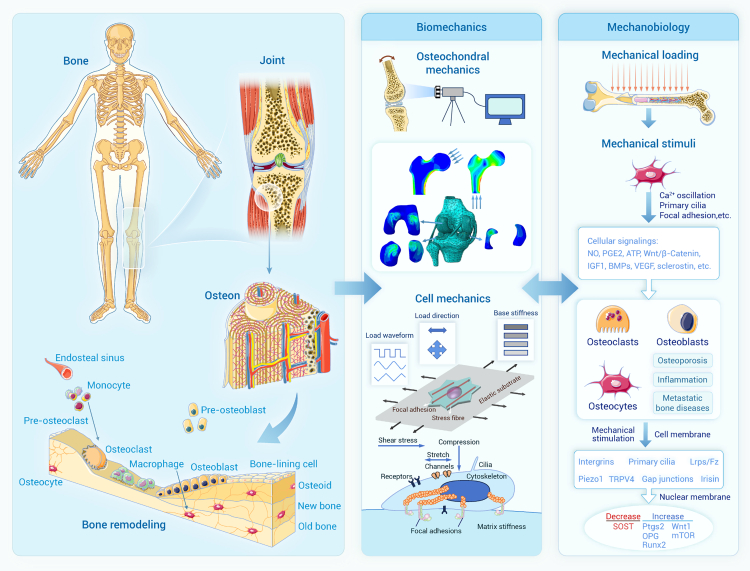
Biomechanics and mechanobiology in bones and joints

Bones and joints, as key load-bearing structures enabling support and movement, are regulated by mechanical loads from their growth and development to injury and aging.^88^ Therefore, knowledge of biomechanical properties and mechanobiological responses of bone and joints is critical to gain insight into their physiological function and pathological process. With the rapid advancement of science and technology, new theories and methods have been applied to biomechanical research on bone and joints,^89^ leading to significant progress and breakthroughs in various aspects, including research scope, methodology, and content.^90^ At present, the mechanobiology of bone and joints is being developed in depth (Figure 2), which has enhanced our understanding of fundamental mechanisms underlying, e.g., bone development, remodeling, and adaptation.^91^

#### Biomechanical studies of bone and joints

Significant progress has been made in biomechanical research on bone and joints. So far, the methods for testing the anisotropy and micro-mechanical properties of bone and cartilage tissue have been well established using different loading devices (including static/dynamic compression, tension, sliding, and rolling) in combination with non-contact digital correlation techniques.^92^^,^^93^ These methods have been applied to biomechanical studies on healthy, degenerated, defective, and repaired bone and cartilage, yielding their quasi-static and dynamic mechanical properties across multiple scales. It has been well established that bones exhibit anisotropic, viscoelastic, creep-recovery, and relaxation behavior in response to mechanical loadings, and the material and mechanical properties of bones vary under different loading conditions, due to their complex biological structures and biochemical composition. Meanwhile, the stress and strain thresholds for initial damage and the load thresholds for irreversible mechanical deformation of bone and cartilage have been determined.^94^ These mechanical performance indicators serve as benchmarks for the construction of engineered bone and cartilage tissue.^95^

At the cellular level, the mechanical properties of cells (e.g., osteoblasts, osteoclasts, osteocytes, and chondrocytes) in bone and joint tissues are also affected by their surrounding mechanical stimuli, such as compression, tension, shear stress, and ECM stiffness, under both physiological and pathological conditions. For example, the elastic modulus of osteocytes and chondrocytes has been established to be 1–2 and 0.5 kPa, respectively.^96^ When subjected to cyclic stress, osteocytes and chondrocytes undergo cytoskeletal rearrangements. This process increases the stiffness of osteocytes and alters the viscoelastic properties of chondrocytes, thereby maintaining the elasticity and compressibility of bone and cartilage.^97^^,^^98^ However, under pathological conditions, the mechanical microenvironment where the cells reside can be changed, subsequently exerting adverse effects on cellular mechanical properties, function, and metabolism.^97^^,^^99^^,^^100^ For the case of osteoporosis and other bone metabolic diseases, abnormal mechanical stimuli to osteocytes result in a reduced cellular elastic modulus and stiffness, which, in turn, weakens their ability to resist deformation.^99^ In contrast, in the case of osteoarthritis, the articular cartilage endures prolonged and excessive exposure to abnormal mechanical stimuli such as excessive pressure and friction. Chondrocyte elastic modulus and stiffness are significantly increased as a result of the changed mechanical properties of the cell membrane, cytoplasm, and cytoskeleton.^97^

Due to the complexity, diversity, and multiscale features of bone and joints, mechanical modeling and simulations, in addition to experimental investigations, have played a significant role in elucidating the mechanical behavior of bone and joints. Bone structure morphology can be accurately obtained by clinical computed tomography (CT) and magnetic resonance (MR), and the heterogeneous, anisotropic material properties of bone tissue are derived from mechanical tests to establish the relationship between the mechanical parameters of bone tissue and the grayscale values in images. Based on this, nonlinear finite element models using clinical medical images can be developed to accurately calculate bone strength.^101^^,^^102^^,^^103^ In recent years, studies have extracted material distribution, geometric morphology, and even radiomic features from clinical CT images as inputs, with bone strength calculated from finite element analyses as the output, to establish machine learning models such as artificial neural networks and support vector machines. These models can be more conveniently applied in clinics to evaluate bone strength and fracture risk.^104^^,^^105^^,^^106^

While macroscopic finite element models are widely used to assess overall bone strength, a full understanding of bone responses under mechanical loading necessitates the integration of cellular-level processes and multiscale modeling approaches. For example, osteocytes, the key mechanosensory cells in bone, detect mechanical signals and initiate biochemical responses.^107^ Sub-microscopic-scale finite element models have been developed to characterize the changes in mechanical stimulation perceived by osteocytes during bone disease progression and to quantify the response of osteocytes to the local mechanical environment.^108^^,^^109^ However, such models fail to capture the complex mechanical microenvironment surrounding osteocytes *in vivo*. To address this issue, multiscale finite element modeling offers a promising framework for linking macroscopic loading to cellular-level mechanical responses. For instance, to investigate the mechanical response of bone tissue across age groups, Cen et al. have recently constructed a multiscale finite element model spanning four hierarchical levels—from the proximal femur (macroscale) to cortical bone (mesoscale), the Haversian system (microscale), and the osteocytic lacuna-canalicular system (sub-microscale).^110^ Another study developed a multiscale computational model of the osteon, incorporating the Haversian canal and the lacuna-canalicular system, with detailed representations of the osteocyte cytoplasm, nucleus, and cytoskeleton.^111^ These advancements underscore the critical role of multiscale modeling in bridging structural and cellular biomechanics and highlight its potential as a powerful tool for providing further insight into the structure, function, and pathology of bone and joints. AI- and machine-learning-integrated computational modeling enables substantial reductions in computational time while maintaining high predictive accuracy. Recently, Awal et al. developed a machine-learning-based modeling approach integrating the finite element method (FEM) for hip fracture risk assessment and visualization. Their finding highlights the efficacy of machine-learning-based surrogate modeling for predicting hip fracture risk and visually identifying fracture locations even under limited training data conditions.^112^ In addition, the AI- and machine-learning-integrated modeling has also been applied to assess femoral strength,^113^ bone remodeling,^114^ and distal radius fracture healing.^115^ These algorithms offer a promising complementary approach for efficiently investigating the biomechanics of bone and joints.

#### Mechanobiological studies of bone and joints

Bones and joints constantly respond and adapt to changes in mechanical environments. Their formation, growth, remodeling, adaptation, and regeneration are closely associated with cell mechanobiology. Over the past several decades, many cell types residing in bone and joint tissues, such as osteocytes, osteoblasts, osteoclasts, chondrocytes, and their progenitors, have been identified as capable of sensing mechanical loading and orchestrating gene expression, protein synthesis, matrix production, and cell proliferation, differentiation, and death.^116^ Several cellular mechanosensing mechanisms have been established on these cells, including ion channels, integrins, G-protein-coupled membrane receptors, primary cilia, gap junctions, and actin cytoskeleton.^117^ The mechanosensors on these cells function as detectors for receiving mechanical signals and transduce these extracellular signals into intracellular responses via, e.g., calcium (Ca^2+^), NO, Wnt, and mitogen-activated protein kinase (MAPK)-dependent pathways.^117^ Emerging evidence suggests that mechanical sensing protein Piezo1 is also involved in regulating bone remodeling.^118^ Wang et al. reported that Piezo1 in osteoblasts controls the YAP-dependent expression of type II and IX collagens to coordinate osteoblast-osteoclast crosstalk, osteoclast differentiation, and bone homeostasis in response to mechanical load. Piezo1 deficiency leads to increased fracture risk, bone loss, and resorption.^118^ Consistent with these observations, Li et al. showed that Piezo1 in both osteoblasts and osteocytes can sense and respond to fluid shear stress. Piezo1 deletion results in a reduction in bone formation and mass by controlling Wnt1 expression via a transcriptional coactivator with PDZ-binding motif (TAZ) and YAP1.^119^ These findings together highlight the important role of Piezo1 in bone homeostasis and provide an alternative target for bone therapy. Recently, Li et al. found that variation in extracellular osmolarity arising from changes in the surrounding fluid environment affects osteoblast migration through TRPV4-Rho/ROCK signaling, providing important insight into the biomechanical mechanism regulating bone formation.^120^

The progression of bone and joint diseases is accompanied by altered mechanical environments, mechanosensation mechanisms, and biochemical responses.^117^ *In situ* experiments show that bone loss during early-stage osteoporosis results in elevated mechanical stimuli sensed by osteocytes and osteoblasts, which are proposed to trigger a compensatory mechanobiological response designed to restore the mechanical environment of bone tissue.^121^^,^^122^ In addition to changes in the mechanical environments, perturbations in the biochemical environment also occur during osteoporosis. For example, circulating estrogen levels are reduced during osteoporosis, an important factor proven to influence the responsiveness of bone cells to mechanical stimuli.^123^^,^^124^^,^^125^ *In vitro* studies indicate that estrogen enhances the shear-stress-induced activation of extracellular signal-regulated kinase (ERK) and p38 MAPK pathways and gene expression of c-Fos and Cox-2 through the estrogen receptor-mediated expression of β1-integrin.^123^ In a separate study, estrogen deficiency inhibits fluid-flow-induced [Ca^2+^]_i_ oscillations and mechanobiological responses of osteocytes characterized by attenuated downstream signaling of NO and prostaglandin E2 (PGE2), which ultimately affect osteocyte function and differentiation.^124^ In addition, osteoblasts are observed to show an impaired proliferative response to mechanical strain in an estrogen-deficient environment.^125^ These results together highlight the synergistic roles of estrogen and mechanical stimuli in regulating signal transduction and gene expression. Much effort has also been devoted to other bone and joint diseases, including osteoarthritis, osteosarcoma, metastatic bone diseases, and fracture healing.^91^^,^^117^^,^^126^^,^^127^^,^^128^^,^^129^^,^^130^ For example, Mathavan et al. have recently investigated gene expression as a function of the local strain magnitude within a mechanically loaded fracture site. They found differential gene expression profiles in regions subjected to high versus low strain, and these mechanical regions are associated with bone formation and resorption, respectively. Their findings provide additional insights into the mechano-regulation of fracture healing.^130^ In addition, knowledge from mechanoepigenetics has also advanced our understanding of the role of the dynamic mechanical environment in the regulation of the epigenome in the context of musculoskeletal diseases such as osteoarthritis.^131^ These insights shed light on disease mechanisms and contribute to the development of novel therapeutic strategies for musculoskeletal diseases. Although significant progress has been made in the mechanobiological research of bone and joint diseases, further studies of the mechanobiological responses of bone cells and underlying mechanisms are required to understand the pathogenesis of bone and joint diseases and provide alternative treatment options.

#### Remarks on the future

Biomechanical and mechanobiological research in bone and joints has laid a solid foundation for the repair, regeneration, rehabilitation, and healthcare of bone and cartilage. However, there remains a pressing need for extensive long-term clinical validation studies on human biomechanics and mechanobiology, focusing on, e.g., fracture fixation, prosthesis replacement, and artificial tissue defect repair and regeneration. As medical imaging technology advances, these studies are expected to progress rapidly. A key challenge for future research is how to precisely treat bone and joint diseases through mechanical stimuli, such as ultrasound and shock waves. In addition, the often contradictory roles of mechanical stimuli across different disease states pose an additional major challenge for therapeutic development. This necessitates exploring the detailed responses within the intricate interplay of mechanical, chemical, and biological coupling, delving deeper into the complex signaling pathways and numerous targets within bone cells, as well as clarifying the primary and secondary roles of mechano-regulatory pathways within bone and joints. Moreover, bones and joints interact with multiple organs and tissues in the body, and mechanical factors affect bone growth, development, and reconstruction, which, in turn, influence the biological processes of other organs and tissues. This dynamic interplay is another pivotal area of focus for future research endeavors. In addition, multiple classes of mechanosensitive molecules, such as integrins, cadherins, selectins, and cytoskeletal proteins, exhibit load-dependent binding behaviors. Deeper exploration of molecular binding and force-regulated mechanotransduction in bone and joints will advance our understanding of how mechanical forces govern osteoblast and osteoclast activities, influence cartilage integrity, and drive the development of skeletal pathologies such as osteoporosis and osteoarthritis.

### Eye

**Figure 3:**
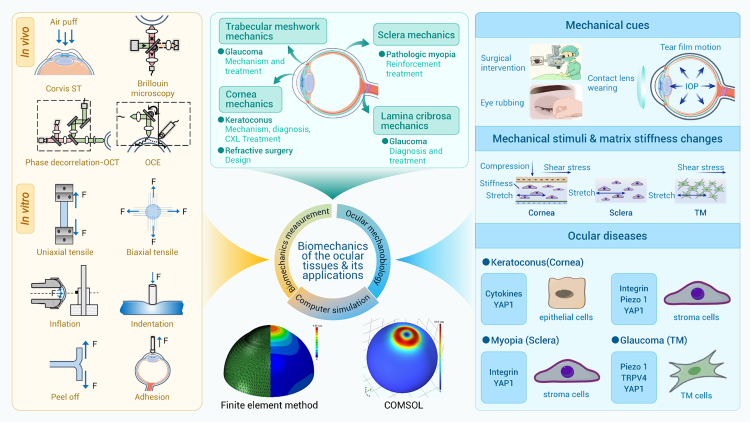
Biomechanics and mechanobiology in eye

Biomechanical homeostasis of ocular tissues is essential for maintaining the shape of the eye and supporting normal physiological functions. Ocular tissues are continuously subjected to complex mechanical forces generated by intraocular pressure (IOP), and their mechanical responses, in turn, can influence IOP regulation through biomechanical feedback mechanisms (Figure 3). To preserve normal visual functions—such as light refraction and optic nerve signal conduction—it is critical that these tissues respond to IOP with appropriate levels of deformation: neither excessive nor insufficient. Disruptions to this delicate mechanical balance can lead to various ocular pathologies. For example, elevated IOP may compress the optic nerve head through the lamina cribrosa, contributing to the development of glaucoma. Similarly, thinning or biomechanical weakening of the cornea is associated with keratoconus (KC), while refractive surgeries must account for postoperative corneal load-bearing capacity to ensure long-term safety and efficacy. Investigating the structure-function relationships of ocular tissues from biomechanical and mechanobiological perspectives across multiple scales is vital for understanding both physiological regulation and disease progression, as well as for advancing diagnostic and therapeutic strategies in ophthalmology.

#### Biomechanical and mechanobiological studies of the cornea and sclera

As the main load-bearing connective tissue of the eyeball, the cornea and sclera are centrally important for maintaining the shape of the eyeball and refractive status. Alterations in the structure and biomechanical properties of the cornea and sclera are closely associated with vision impairment and can even lead to blindness. The cornea and sclera are primarily subjected to tensile forces along the tangential direction of the eyeball and exhibit typical nonlinear mechanical features. Generally, hyperelastic mechanics models, such as the Mooney-Rivlin, neo-Hookean, and Ogden models, can be used to describe the quasi-static behavior of cornea and sclera. Meanwhile, the cornea and sclera are also typical viscoelastic solid materials, exhibiting creep and stress relaxation behaviors. These can be described by linear viscoelastic models or quasi-linear viscoelastic models.

Biomechanical research into the cornea and sclera in patients is particularly important for eye disease diagnosis and treatment. As a typical anisotropic tissue, the tangent modulus of the cornea in the horizontal direction is observed to decrease more obviously than that in the vertical direction with the increase of axial length for patients with myopia.^132^ Reduced mechanical property of the sclera is a characteristic of high myopia, leading to excessive elongation of the eyeball axis under IOP and a series of ocular fundus lesions, such as retinal degeneration and detachment. Currently, therapies targeting the mechanical properties of the cornea and sclera have been proposed to treat related ocular diseases. Take, for example, collagen cross-linking (CXL), a process that can enhance tissue stiffness by creating additional chemical bonds within the tissue. CXL has been widely adopted for the treatment of KC, with its efficacy in halting or delaying the disease’s natural progression confirmed.^133^ However, due to the non-uniformity of the corneal structure and mechanical properties at the site of corneal protrusion in KC, a standard broad-beam CXL pattern does not address myopic and astigmatic refractive changes resulting from the abnormal corneal shape. Therefore, personalized cross-linking by controlling the riboflavin penetration concentration and ultraviolet irradiation intensity at different sites of the cross-linked area has emerged,^134^^,^^135^ which can solve the above problems by improving local mechanical properties of the cornea as needed. As a promising alternative for the treatment of progressive myopia, the efficacy and safety of riboflavin/ultraviolet-A sclera cross-linking have been demonstrated in *in vitro* and *in vivo* animal experiments.^136^^,^^137^^,^^138^ Recently, a preliminary study on blind human eyes has also confirmed its feasibility and safety.^139^ Future efforts should be made to verify its long-term surgical outcomes, including its efficacy, stability, and safety. In this regard, the mathematical modeling and computer simulations have made important contributions. In particular, the FEM has proven to be an effective tool for analyzing the mechanical responses of ocular tissues. For example, FEM-based analysis of stress and strain distribution across the cornea has informed the design of refractive surgery, CXL surgery, and orthokeratology (OK) lenses, as well as the evaluation of surgery safety.^140^

Determining the *in vivo* mechanical characteristics of the cornea and sclera is another research focus from the perspective of clinical application. Many measurement techniques have been developed, including the air-puff method (e.g., ocular response analyzer [ORA] and Scheimpflug technology [Corvis ST]), ultrasound surface wave elastometry (USWE), and optical techniques (phase-decorrelation optical coherence tomography [PhD-OCT] and Brillouin microscopy) (Figure 3). Each technique has its own pros and cons. ORA and Corvis ST are commercial products already widely applied in clinical practice.^141^ Combined with the stress-strain index (SSI) values obtained via Corvis ST and geometric information via corneal topography, an SSI map is derived using finite element inverse analysis considering collagen distribution. It allows visualization of the stiffness distribution across the cornea surface in both healthy subjects and patients with KC.^142^ It is expected to become a tool for evaluating KC progression and the efficacy of customized treatments.^143^ Although OCT-based technologies, Brillouin microscopy, and optical coherence elastography have also been developed to assess *in vivo* scleral biomechanical properties, they are still in their infancy. Recently, an ultrasound elastography (UE) system with high resolution has been developed to simultaneously evaluate the biomechanical properties of multiple ocular structures and detect dynamic alternations in these biomechanical properties. It could serve as a useful tool for providing a comprehensive, 3D analysis of the ocular biomechanics and geometric parameters.^144^ The air-puff test is a contactless tonometry technique frequently used to assess the cornea’s biomechanical properties, wherein accurately estimating the distribution of air pressure on the cornea is essential. To overcome the time-consuming nature of the CFD-based fluid-structure interaction model, Desouky et al. incorporated a supervised regression machine learning algorithm, achieving comparable accuracy while markedly improving computational efficiency.^145^ Integration of physics-informed neural networks (PINNs) with this machine learning algorithm could help expand the model to a larger dataset, thereby improving the accuracy of corneal material properties predictions. Current *in vivo* biomechanical measurements using existing techniques may only capture partial mechanical behaviors of the cornea and sclera, particularly under dynamic loads. New methods for evaluating these properties under quasi-static loading are still needed.

Cells within the cornea and sclera can sense and respond to their surrounding mechanical environments, such as tension, compression, and matrix stiffness. Depending on their location within the cornea and sclera tissue, the mechanical cues perceived by cells within the cornea and sclera may differ. In response to these mechanical stimuli, changes occur in cell morphology, proliferation, differentiation, matrix production, gene expression, and protein synthesis.^146^ It is also found that the different mechanical environments can enable cells to respond variably to biochemical environments. A recent study indicated that keratocyte phenotype and differentiation exhibit a substrate-stiffness-dependent behavior in response to inflammatory factor IL-1β, providing deeper insight into corneal pathology and repair.^147^ So far, multiple signaling pathways, including those of Hippo, YAP/TAZ, TGF-β, RhoA, Wnt, protein kinase B (AKT), and Piezo1, are proposed to play important roles in mechanobiological responses.^146^ Abnormal mechanical environment in disease states, such as increased IOP and altered ECM composition, could exert a detrimental effect on resident cells and may ultimately result in cellular dysfunction through different mechanotransduction pathways discussed above. A recent study by Markov et al. exposed scleral fibroblasts to a physiological load and a pathological load, with the former mimicking the healthy IOP and the latter mimicking the elevated glaucomatous IOP.^148^ Their results indicate that physiological strain triggers a transient rearrangement of the F-actin cytoskeleton, whereas pathological strain reversibly postpones this rearrangement and concurrently induces increased chromatin condensation. This, in turn, could disrupt normal cellular remodeling and contribute to glaucomatous pathology.^148^ Other studies have revealed that the pathological mechanical environment in myopia can inhibit mitotic activity of scleral fibroblasts and downregulate the collagen synthesis, leading to scleral remodeling and thinning via, e.g., TGF-β1, Wnt3/β-catenin, and hypoxia-inducible factor 1α (HIF-1α) signaling pathways.^149^^,^^150^ Liu et al. recently showed that ECM stiffness regulates scleral remodeling in myopia through the integrin α1β1-F-actin-YAP axis, which targets COL1A1 expression in scleral fibroblasts.^151^ This finding highlights the roles of integrin α1β1 and F-actin in ECM-stiffness-regulated YAP activation and advances our understanding of how mechanical forces govern scleral remodeling in myopia. Furthering our understanding of corneal and scleral mechanobiology will undoubtedly contribute to the development of innovative treatment and clinical strategies.

#### Biomechanical and mechanobiological studies of the lamina cribrosa

The lamina cribrosa, tethered to the posterior sclera across the scleral canal in the optic nerve head, is a mesh-like connective tissue through which the central retinal vessels and retinal ganglion cell axons pass.^152^ This connective tissue consists of multilayered, orientation-dependent collagen fibrils and is subjected to IOP, intracranial pressure (ICP), and in-plane pretension.^153^ In response to such mechanical loadings, the lamina cribrosa deforms and remodels, accompanied by collagen synthesis and degradation. The heterogeneous, nonlinear, and viscoelastic properties of the lamina cribrosa give rise to rate- and time-dependent mechanical behavior.^154^

The lamina cribrosa is the primary site of glaucoma, the leading cause of irreversible blindness worldwide.^153^ The pathological mechanism underlying glaucoma is substantially attributed to the irreversible deformation of the lamina cribrosa, thus highlighting the significance and necessity of studying the deformation process and state of the lamina cribrosa. Although imaging techniques such as optic disc photography, confocal scanning laser ophthalmoscopy, and OCT have provided us with important information on the macro- and micro-architectural characteristics of the lamina cribrosa,^155^ it is rarely feasible to conduct real-time measurement of the stress and deformation state of the lamina cribrosa *in vivo* for the purpose of clinical diagnosis and treatment of glaucoma. Developing appropriate mechanical models remains an efficient way. So far, many mechanical models have been developed based on, e.g., thin-film theory and Kirchhoff’s thin-plate theory. Unfortunately, all of the results obtained fail to agree well with existing experimental results, partially due to neglecting the contribution made by the shear effects in the lamina cribrosa. To solve such an issue, Tian et al. developed a new mechanical model according to Reissner’s thin-plate theory.^156^ Their results are in good agreement with the experimental data and show the important role of shear deformation in optic nerve injury. They found that the shape of the optic nerve channel transforms from a straight cylinder into a tortuous elliptical horn, and the dislocation of the laminar sheets is large enough to damage the optic nerve axons. Their findings theoretically verify the clinical speculation and further support the mechanical theory of glaucomatous damage. Other mathematical models have also been developed to investigate the influence of the deformation of the laminar cribrosa on the hemodynamics in central retinal vessels.^157^^,^^158^ These studies have provided insight into the biomechanics-hemodynamics relationship within the laminar cribrosa. To more accurately describe the mechanical response of ocular tissues, substantial efforts have been made to incorporate microstructures into computational models.^154^^,^^159^^,^^160^^,^^161^ A recent microstructural FEM model has been developed to enable separate analysis of the mechanical behavior of the lamina cribrosa and neural tissues.^159^ Simulation results indicate that the prior homogenized continuum model underestimates the maximum strains in the lamina cribrosa beams and neural tissues by a factor of 2–3 compared to the microstructural FEM model, providing more insight into the pathogenesis of glaucoma.^159^

The dynamic deformation and remodeling of the laminar cribrosa under physiological and pathological conditions influence resident cells, such as astrocytes, laminar cribrosa cells, and microglia, via, e.g., integrins, mechanosensitive ion channels, and G-protein-coupled membrane receptors.^162^^,^^163^^,^^164^^,^^165^^,^^166^^,^^167^^,^^168^^,^^169^ Signaling pathways such as YAP/TAZ, MAPKs, protein kinase C (PKC), phosphoinositide 3-kinase (PI3K) have been identified to be involved in these mechanotransductions.^165^^,^^166^ In particular, the ECM architecture and composition within the glaucomatous lamina cribrosa change significantly in response to elevated IOP, with backward displacement and increased amounts of TGF-β2, elastin, proteoglycan, and collagen. This pathological fibrotic remodeling of glaucomatous lamina cribrosa results in marked alterations in the mechanical environment (e.g., stretch and ECM stiffness) perceived by resident cells.^170^^,^^171^ Mounting evidence shows that the astrocytes and laminar cribrosa cells play integral roles in lamina cribrosa remodeling when exposed to abnormal mechanical loading, TGF-β, and oxidative stress in glaucoma.^162^^,^^164^ These two types of cells are significant sources of ECM production and can express elevated levels of ECM composition, such as collagen, laminin, and fibulin, in glaucomatous lamina cribrosa compared to normal conditions.^162^^,^^164^ The exact functions of the astrocytes and laminar cribrosa cells in disease progression, beneficial or detrimental, remain to be fully explored. The answer will further provide potential therapeutic targets for glaucoma treatment. Growing evidence indicates an interplay of genetic and epigenetic factors in the pathology of glaucoma.^172^ Aberrant histone modifications, methylation patterns, and post-transcriptional processing change gene expression and contribute to the progressive course of glaucoma. Although studies on genetic and epigenetic regulation in glaucoma are just emerging, the genetic and epigenetic modifications are evidently integral to glaucoma pathogenesis and may hold promise for informing more targeted therapeutics.^172^

#### Biomechanical and mechanobiological studies of the trabecular meshwork

The trabecular meshwork (TM), located in the anterior chamber angle of the eye, is a fenestrated tissue that consists of multilayered beams or lamellae covered by TM cells. TM, as the primary site of aqueous outflow resistance, plays a crucial role in regulating aqueous humor drainage and maintaining IOP homeostasis. TM dysfunction results in increased outflow resistance and IOP elevation, which is the primary risk for glaucoma. We can utilize uniaxial tensile tests, AFM, OCT, and optical coherence elastography technology to measure the mechanical properties of the TM, which can be regulated by ECM component, age, genetic and gene mutation, cellularity, etc.^173^ AFM results show that the elastic modulus of glaucomatous TM is twenty times larger than that of normal TM.^174^ A recent study by Karimi et al. investigated the viscoelastic mechanical behavior and revealed significantly larger shear moduli in the glaucomatous ECM compared to the healthy controls.^175^ More importantly, existing results indicate that the mechanical properties of the TM are closely related to the outflow resistance and IOP elevation.^173^^,^^176^^,^^177^ These findings suggest that understanding the mechanical properties of the TM may serve as a potential target for the diagnosis and treatment of glaucoma.

Normal TM cells, as good biomechanosensors, sense and respond to mechanical stimuli such as IOP, stretch, shear, and osmotic stress, leading to changes in cellular activities including gene expression, adhesion, migration, proliferation, differentiation, and apoptosis.^178^ TM cells are often exposed to shear stress generated by aqueous humor flow.^179^ Yarishkin et al. reported that Piezo1 functions as the primary transducer of shear stress in the TM and is involved in dynamic control of TM cytoskeleton organization, focal cell-ECM contact, and aqueous outflow.^180^ Particularly, Piezo1 blockade by grammostola spatulata mechanotoxin 4 (GsMTx4) significantly decreased the outflow facility.^180^^,^^181^ In contrast, Piezo1 activation by agonist Yoda1 leads to a remarkable IOP-lowering effect, accompanied by increased matrix metalloproteinase (MMP)-2 expression and suppressed TM cell migration and proliferation.^182^ In addition, shear stress can also contribute to IOP homeostasis by modulating TRPV4-eNOS signaling.^183^ Recently, Du et al. revealed that cellular senescence impairs the mechanobiological response of TM cells to shear stress, establishing the correlation between dysfunction of TM and age.^184^ Other mechanical stimuli, such as ECM stiffness and stretch, significantly influence TM cell activity as well.^185^^,^^186^^,^^187^ For instance, alternation in ECM stiffness can affect TM cell morphology, adhesion, proliferation, and cytoskeleton remodeling through multiple signaling pathways, including those of YAP/TAZ, Rho/Rho-associated protein kinase (ROCK), Wnt, and ERK.^187^^,^^188^^,^^189^ The transcription complex formed by, e.g., YAP/TAZ in the nucleus activates various target genes to promote ECM remodeling, which can further aggravate TM stiffness. This will, in turn, increase aqueous humor outflow resistance and elevate IOP, thus exacerbating the progression of glaucoma.^188^ Recently, Xu et al. showed that TRPV4 activation engages the PI3K/AKT pathway, which in turn regulates YAP’s nuclear translocation and fibrotic gene expression. This study identifies the TRPV4-PI3K/AKT-YAP signaling axis as a critical regulator of TM cell stiffness and IOP homeostasis and highlights this pathway as a promising therapeutic strategy for reducing IOP and improving TM function.^190^ Collectively, these findings help to identify novel potential targets for IOP control and glaucoma treatment.

#### Future remarks

Understanding the pathogenesis of ophthalmic diseases—such as glaucoma, KC, and progressive myopia—and the design of clinical interventions, including refractive surgery, corneal cross-linking for KC, and posterior scleral reinforcement for pathological myopia, requires support from ocular biomechanics and mechanobiology. Many challenges remain to be addressed. For instance, investigating how lamina cribrosa and TM cells respond to mechanical stimuli will be beneficial to reveal the underlying pathogenesis of glaucoma. New potential therapeutic targets also need to be identified through studies on mechanosensitive factors and related pathways that regulate IOP or remodel the lamina cribrosa microstructure. Many mechanosensitive molecules, including integrins, cadherins, selectins, and cytoskeletal proteins, exhibit force-dependent binding behavior that tunes cellular mechanotransduction and responses. Expanding molecular-level investigations of binding biomechanics and force-dependent signaling in ocular tissues will deepen our understanding of how mechanical cues shape TM function, influence optic nerve head homeostasis, and contribute to disorders such as glaucoma, myopia progression, and corneal degenerative diseases. Moreover, accurate characterization *in vivo* of the mechanical behavior of both the cornea and sclera—totally and locally—will greatly enhance our ability to predict their health status and develop personalized treatment plans. Meanwhile, improving the widely used Brillouin microscope, developing new ultrasonic and corneoscleral mechanical testing devices, and ongoing research integrating the FEM with AI to analyze air-puff experimental data all offer promising solutions for achieving reliable and clinically meaningful results. In addition, building multiscale biomechanical models of ocular tissues will contribute to a better understanding of their interconnected behaviors and responses, as well as the underlying mechanisms of disease pathogenesis.

### Liver

**Figure 4:**
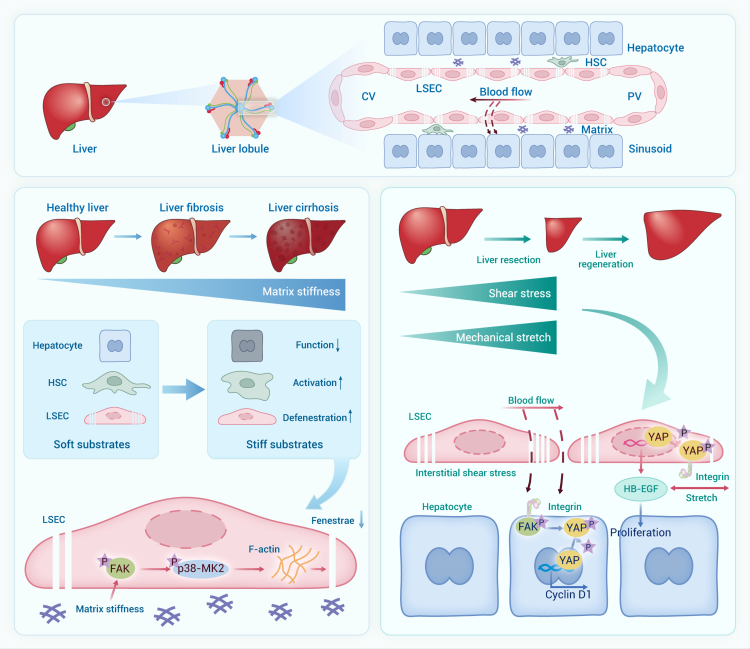
Biomechanics and mechanobiology in the liver

The liver is a unique organ in the human body, endowed with many functions including biosynthesis, metabolism, digestion, and detoxification, which are highly reliant on the complex microstructures, multiple cell-cell interactions, and sophisticated mechanical niches.^191^^,^^192^ As its basic elements, the hepatic lobules form the structural and functional units of the liver, comprising the hepatocytes radially arrayed in hepatic plates and the hepatic sinusoids interspersed therebetween.^193^^,^^194^ The sinusoid is a specialized capillary network lined by highly permeable liver sinusoidal ECs (LSECs) enriched with open fenestrae (with a diameter ranging from 50 to 300 nm), retaining the material exchange between the blood flow and hepatocytes.^195^^,^^196^ Kupffer cells are resident in the sinusoids and exert the function of immune surveillance, while quiescent hepatic stellate cells (HSCs) reside in the space of Disse between the hepatocytes and the sinusoids, generating minimal ECM in a healthy liver.^197^^,^^198^ Recently, a growing body of evidence suggests that the mechanical microenvironment, including matrix stiffness, fluid shear stress, and mechanical stretch, serves as a significant factor governing liver injury and regeneration (Figure 4).^191^^,^^194^^,^^199^^,^^200^^,^^201^^,^^202^

#### Biomechanical studies of the liver

The liver exhibits a complex biomechanical behavior arising from its heterogeneous architecture and flexible boundary conditions. Like most biological soft tissues, the liver exhibits viscoelastic and hyperelastic mechanical properties. Determining and quantifying the mechanical properties of the liver is, however, highly challenging due to its intrinsic labile nature. The measured mechanical properties of the liver vary considerably, depending strongly on the testing method and tissue model adopted.^203^ Currently, characterization of liver mechanical properties relies on direct mechanical tests (e.g., tension, compression, indentation, shear, and aspiration), MRI, and ultrasound-based techniques, with data commonly interpreted using viscoelastic frameworks such as the generalized Maxwell model, Kelvin-Voigt fractional derivative model, and porous visco-hyperelastic model.^203^ The development of pathophysiologically relevant tissue models, along with standardized guidelines for mechanical testing, is essential to generate reproducible and unbiased results.

Studying the biomechanical response of the liver is essential for understanding its mechanical behavior under physiological and pathological conditions and guiding surgical procedures. Many experimental efforts have been made to evaluate the mechanical response and injury tolerance of the liver under loading conditions such as compression and tension, providing important information regarding the mechanical deformation and fracture behavior.^204^ Meanwhile, the FEM, incorporating polynomial models, exponential and logarithmic models, the Veronda-Westmann model, the neo-Hookean model, and the Mooney-Rivlin model, has also been employed to simulate liver responses under complex loading conditions.^205^^,^^206^ These numerical studies have provided important insights into how the mechanical factors affect the biomechanical and failure responses of the liver parenchyma. However, the high computational cost of these numerical models limits their real-time applicability, such as in virtual surgery simulations. AI and machine learning have provided a powerful means of addressing this challenge. Zhu et al. proposed a feasible machine learning modeling scheme that achieves good real-time performance in predicting liver deformation under various loading conditions, offering high computational efficiency while maintaining FEM-level numerical accuracy.^207^ Recently, Hu et al. developed a neural-network-based real-time multi-physics modeling framework for simulating liver deformation and visualizing stress distribution. Compared to traditional methods, their method achieves a 1,000- to 10,000-fold improvement in computational efficiency, with only about a 1% loss in accuracy.^208^ The integration of computational modeling and AI holds great promise for real-time, patient-specific biomechanical assessment, contributing to improving clinical decision-making and surgical outcomes.

#### Mechanobiological studies of the liver

Mechanobiological studies of the liver have provided important insights into the molecular mechanisms underlying liver physiology and pathology. Here, we focus on liver fibrosis and generation to show how biomechanical cues drive cellular responses and disease progression.

Liver fibrosis is a pathological repair response marked by the excessive deposition and abnormal distribution of ECM, constituting a vital step in the progression of various chronic liver diseases toward liver cirrhosis.^24^^,^^191^ To date, no specific and effective therapies for liver fibrosis have been widely accepted in the clinic. Enhanced liver tissue stiffness represents the most notable mechanical alteration during liver fibrosis and has emerged as an outstanding biomarker for clinical diagnosis.^201^ Through non-invasive elastography, it can be determined that the elastic modulus of normal liver tissue is typically less than 6 kPa, ascending to 6–12.5 kPa in liver fibrosis and reaching 12.5 kPa for cirrhotic patients.^209^ In the rat liver fibrosis model induced by carbon tetrachloride (CCl_4_), the upregulation of liver tissue stiffness caused by ECM cross-linking occurs even prior to significant collagen deposition and plays an important role in promoting fibrosis development.^210^ High stiffness modulates the phenotypes of hepatic cells by inducing nucleus deformation through cytoskeleton-derived mechanical forces.^211^ Stiff substrates inhibit hepatocyte-specific functions such as albumin production, glycogen storage, and cytochrome P450 activity by downregulating the hepatocyte nuclear factor 4 alpha (HNF4α) transcriptional network mediated through the focal adhesion kinase (FAK) and ROCK pathway.^212^ HSCs grown on rigid substrates undergo differentiation into contractile myofibroblasts, which are characterized by elevated levels of α-smooth muscle actin (α-SMA) and an excessive accumulation of ECM.^213^ A recent study investigated the effect of ECM stiffness on genome-wide chromatin accessibility and gene expression in HSCs and demonstrated the cooperative effects of ECM composition and stiffness on H3K4 and H3K9 methylation/acetylation.^214^ In addition, this study revealed higher chromatin accessibility in HSCs on relatively soft substrates and identified two candidate regulatory factors, CEBPb and HSD11B1. Furthermore, Zhao et al. found that AP-1-induced chromatin priming mediates fibrogenesis in HSCs in response to increased matrix stiffness.^215^ Typically, it is only when liver fibrosis advances to the cirrhotic stage that the exceedingly high liver stiffness will facilitate the progression of hepatocellular carcinoma (HCC).^216^^,^^217^ Nevertheless, in metabolic dysfunction-associated steatotic liver disease (MASLD), lipid droplets function as intracellular mechanical stressors in a manner similar to matrix stiffening, impairing hepatocyte function and triggering nuclear rupture, which might contribute to the appearance of hepatocarcinogenesis at the non-cirrhotic stage.^200^^,^^217^^,^^218^ For patients with MASLD and type 2 diabetes mellitus (T2DM), an accumulation of advanced glycation end-products (AGEs) enhances ECM viscoelasticity with faster stress relaxation, promoting HCC progression in non-cirrhotic conditions.^216^

Moreover, matrix stiffening is also known to induce functional and phenotypic dysregulations of LSECs. The increased matrix stiffness recruits glycolytic enzyme phosphofructokinase 1 isoform P (PFKP) to the focal adhesion sites, facilitates the formation of stress fibers, and induces C-X-C chemokine ligand 1 (CXCL1) expression through nuclear pore expansion and an increase in nuclear factor-κB (NF-κB) translocation, thereby promoting neutrophil infiltration and the development of portal hypertension.^219^ LSECs cultured on substrates mimicking the stiffness of an early-stage fibrotic liver yield a tendency to form capillary-like structures, which in turn leads to HSC activation through collagen fibril remodeling.^220^ Furthermore, the defenestration of LSECs constitutes an initial and indispensable step in the progression of liver fibrosis, triggering HSC activation and exacerbating liver damage.^221^ A recent study provides a comprehensive elaboration on the mechanotransduction mechanism by which matrix stiffness initiates LSEC defenestration.^195^ In this recent study, soft and stiff polyacrylamide hydrogels with elastic moduli of 1.2 and 75 kPa are employed to mimic the stiffness of healthy and cirrhotic liver tissues *in vitro*. The fenestrae of LSECs cultured on various substrates are quantified by AFM imaging and fully convolutional network (FCN)-based automatic recognition.^196^ Soft hydrogels enable LSECs to maintain a highly differentiated phenotype featured as intact sieve plates, whereas stiff hydrogels facilitate LSEC defenestration, as confirmed by the reduced number of fenestrae and attenuated phenotypic markers.^195^ LSECs can sense the increase in matrix stiffness through FAK, giving rise to the activation of p38 and downstream MAPK-activated protein kinase 2 (MK2). Phosphorylated MK2 further activates LIM kinase 1 (LIMK1) and leads to the inactivation of cofilin. This signaling cascade triggers the remodeling of the actin cytoskeleton and eventually expedites LSEC defenestration. Intriguingly, the inhibition of the FAK or p38-MK2 pathway can effectively restore the fenestrae to a certain extent in LSECs isolated from CCl_4_-induced liver fibrosis mice. This typical study highlights the impact of mechanotransduction in LSEC defenestration and offers potential therapeutic strategies for liver fibrosis from a mechanotransduction perspective.

The liver exhibits a robust regenerative capacity in response to hepatic tissue injury, enabling it to restore the majority of its initial mass within 7–8 days following 70% partial hepatectomy (PHx) in rodent models.^194^ Immediately after PHx, the portal blood flow per unit volume of the residual liver is promptly augmented, resulting in a 3- to 5-fold increase in sinusoidal shear stress estimated by intravital microscopy and enhanced mechanical stretch on LSECs, as evidenced by vascular dilatation.^201^^,^^222^ Consequently, this operation modulates the release of paracrine factors that contribute to the process of liver regeneration.^191^ For instance, the secretion of TGF-β1 in LSECs is significantly reduced in response to the elevated shear stress, thereby facilitating hepatocyte proliferation during the initial stage of liver regeneration.^223^ In contrast, the low shear stress resulting from the incomplete remodeling of liver sinusoids is responsible for the Notch-induced senescence of LSECs, which hinders liver regeneration in the late stage.^224^ On the other hand, the sinusoidal diameter rises with increasing extent of hepatectomy, implying the enhancement of mechanical stretch on LSECs.^225^ Uniaxially stretched LSECs can secrete more hepatocyte growth factor (HGF) through the activated β1 integrin and the phosphorylated vascular endothelial growth factor receptor 3 (VEGFR-3) to promote hepatocyte proliferation.^226^ The application of biaxial stretch to LSECs *in vitro* leads to the upregulation of the gene and protein expression of heparin-binding EGF-like growth factor (HB-EGF) in an amplitude- and duration-dependent manner.^225^ Hepatocytes cultured with the supernatant derived from these stretched LSECs demonstrate the augmented proliferation and the improved functions of synthesis and metabolism. The enhanced HB-EGF expression and hepatocyte proliferation are confirmed by PHx in mice or *ex vivo* liver perfusion. This HB-EGF expression is mediated by YAP nuclear translocation and subsequently binds with TEA domain (TEAD) family transcription factors. YAP enters the nucleus via two F-actin-related transport mechanisms. Firstly, mechanical stretch induces nuclear pore expansion through F-actin remodeling, passively facilitating YAP to enter the nucleus and thereby enhancing the expression of HB-EGF. Secondly, F-actin reorganization upregulates the expression of BAG family molecular chaperone regulator 3 (BAG-3), which in turn binds to YAP and actively promotes YAP nuclear translocation. In this process, β1-integrin serves as a target sensor in stretch-induced mechanotransduction. Both the expression of HB-EGF and liver regeneration after 2/3 PHx are attenuated in EC-specific Yap1-deficient mice, verifying the above mechanical-biological coupling *in vivo*. In fact, stretch-induced LSEC paracrine also contributes to portal hypertension.^227^ Expression of CXCL1 is upregulated in stretched LSECs through integrin-dependent activation of Notch signaling and Piezo channels, resulting in the recruitment of neutrophils, which induce sinusoidal thromboses and promote portal hypertension through the formation of neutrophil extracellular traps (NETs). Besides LSECs, the hepatocytes beneath the sinusoidal endothelium are also competent in detecting the flow-induced mechanical alterations following PHx. In the hepatic sinusoids, the blood flow can traverse the fenestrae or intercellular gaps of the LSECs and enter the Disse’s space to form the interstitial flow, which comes into direct contact with hepatocytes and exerts shear stress on them.^193^ Hepatocyte proliferation can be initiated by enhanced interstitial flow, as revealed by *ex vivo* perfusion of the mouse liver.^228^ Flow-induced shear stress can favor hepatocytes to re-enter the cell cycle *in vitro*, in a shear duration- and amplitude-dependent manner. Shear stress activates FAK through the β1 integrin, and inhibiting the Hippo pathway can upregulate nuclear translocation of YAP under shear, which in turn facilitates hepatocyte proliferation. These findings provide a novel insight into understanding the contributions of mechanical forces in triggering liver regeneration.

#### Future remarks

The studies described above typically focus solely on the role of a single mechanical stimulus in liver injury or regeneration. However, *in vivo*, diverse mechanical cues are often interrelated to modulate hepatic cells.^191^ For instance, the increased interstitial shear flow in cirrhotic livers may magnify stiffness-induced responses of HCC cells, causing the epithelial-to-mesenchymal transition shown by the loss of liver-specific functions.^229^ For future perspectives, several issues are critical to be addressed: (1) can we apply the high-resolution *in vivo* imaging technique, deep learning algorithm, and CFD simulations to facilitate further investigations for liver injury or regeneration? (2) How can those *in vivo* multiple mechanical cues, including matrix stiffness and viscoelasticity, interstitial flow, and vascular stretch, work together to manipulate liver injury or regeneration? (3) How do we integrate the multiscale mechanical regulations, from the viewpoint of mechanosensation and mechanotransduction, to elucidate typical functional changes such as hepatocyte dysfunction, LSEC capillarization, and HSC activation? (4) Can the in-depth exploration of the complex mechanical microenvironments be coupled with the application of emerging technologies, such as organ-on-a-chip^192^^,^^230^^,^^231^ and organoids,^232^^,^^233^ to promote a comprehensive understanding of the mechanical regulation in the liver? In addition, a range of mechanosensitive molecules, such as integrins, cadherins, selectins, and cytoskeletal proteins, undergo structural changes that regulate protein interactions and downstream signaling in response to mechanical forces. Advancing molecular-scale studies of force-dependent binding and mechanotransduction in the liver will provide deeper fundamental insight into cell-cell and cell-ECM interactions. Evidently, deciphering these issues could further our understanding of mechanically modulated mechanisms and potential therapeutic targets for liver injury or regeneration.

### Lung

**Figure 5:**
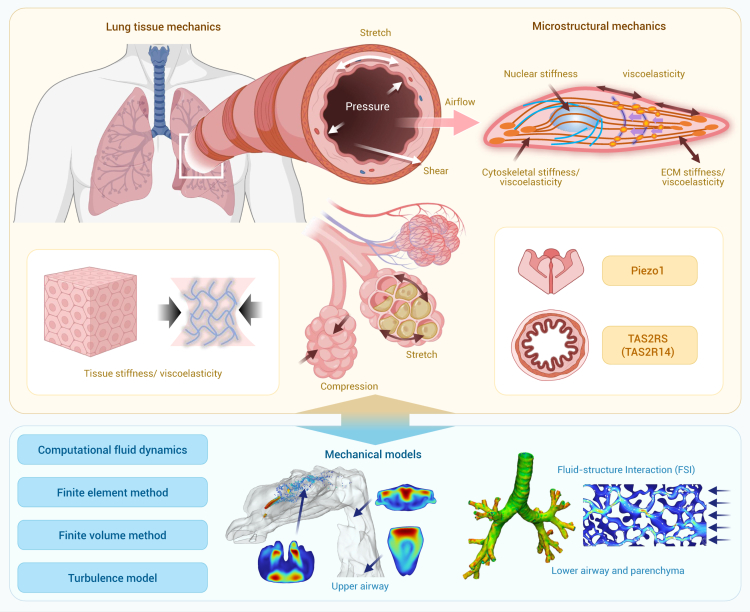
Biomechanics and mechanobiology in the lung

The lung comprises a hierarchically organized network of airways, alveoli, bronchioles, blood vessels, basal membranes, and surrounding ECM.^234^ It is the primary organ of the respiratory system, responsible for extracting oxygen from the inhaled air and transferring it into the bloodstream while simultaneously removing carbon dioxide during the process of gas exchange. Throughout the respiratory cycle, the lung and its associated structures are continuously subjected to complex mechanical forces, including cyclic stretch from ventilation, shear stress from airflow, transmural pressure differentials, and surface tension at the air-liquid interface (Figure 5). These mechanical forces are essential for normal lung function but, when dysregulated, can contribute to the development of various pathologies such as pulmonary fibrosis and emphysema, underscoring the importance of biomechanical and mechanobiological investigations in understanding lung physiology and disease. Recent advances in experimental technologies and computational modeling have propelled research in lung biomechanics and mechanobiology, enabling a deeper understanding of how mechanical cues shape lung development, maintain tissue homeostasis, and contribute to disease pathogenesis. Current research spans multiple structural compartments of the lung, including the airway wall, alveoli, and parenchyma. In this section, we focus specifically on the airway wall, examining its biomechanical properties and contributions to respiratory disease pathogenesis.

#### Biomechanical studies of the lung

The airway wall is a highly organized, multilayered structure that confers both mechanical resilience and functional adaptability to the respiratory tract.^235^ At the microstructural level, the mechanical properties of the airway wall are governed by the composition and organization of the ECM and resident cells. (1) Collagen provides essential tensile strength, forming a fibrous network that limits over-distension while preserving structural integrity.^236^ Its specific alignment allows for controlled flexibility during cyclic breathing, preventing rupture under physiological strain. (2) Elastin confers the lung’s hallmark elastic recoil, enabling efficient return to the resting state after inhalation. Adequate elastin content ensures long-term resilience against repeated deformation, a property critical for sustaining expiratory flow.^237^ (3) Proteoglycans, composed of a core protein and glycosaminoglycan side chains, facilitate lubrication between collagen and elastin fibers.^238^ By regulating interstitial fluid mechanics and dissipating mechanical energy, proteoglycans endow the tissue with viscoelasticity essential for smooth, low-resistance ventilation.

At the tissue level, the mechanical performance of the airway wall arises from the coordinated architecture and material properties of its constituent layers. (1) Mucosa, composed of the epithelium, basement membrane, and lamina propria, forms the luminal interface of the airway.^239^ Its intrinsic compliance enables it to buffer shear forces and pressure fluctuations during breathing. (2) Submucosa consists of loose connective tissue and helically arranged smooth muscle bundles.^240^ These muscle fibers exhibit tightly regulated contractility, allowing fine-tuned modulation of airway caliber in response to neural and humoral cues—without eliciting pathological obstruction or hyperresponsiveness. (3) Adventitia, serving as the outer anchoring interface, mechanically couples the airway to the surrounding parenchyma. This structural tethering provides mechanical support and positional stability, ensuring coordinated movement between conducting airways and alveolar units during ventilation.^241^

It has been well recognized that respiratory diseases, including asthma, chronic obstructive pulmonary disease (COPD), idiopathic pulmonary fibrosis (IPF), and acute respiratory distress syndrome (ARDS),^242^ frequently involve biomechanical alterations of the lung, although the nature of these alterations and their functional consequences vary substantially across conditions. For example, in asthma, airway remodeling—characterized by epithelial shedding, ECM reorganization, and smooth muscle hyperplasia—increases tissue stiffness and viscoelasticity, thereby reducing airway expandability and promoting ventilation heterogeneity.^243^^,^^244^^,^^245^^,^^246^ Similarly, in IPF, the lung features aberrant fibroblast activation and excessive collagen deposition,^247^^,^^248^ which drastically elevate the stiffness and diminish the elasticity and viscoelasticity of the lung tissue, thereby impairing the vital capacity of the lung.^249^ These findings underscore the important role of biomechanics in understanding and managing respiratory diseases.

Mechanical modeling and computational simulations have led to major new insights into the complex behavior of the airway wall and the dynamics of intrapulmonary airflow, advancing our understanding of human respiratory physiology and pathophysiology. Evolving from simplified frameworks such as algebraic or reduced-dimension models, lung modeling and simulations have progressed significantly, with a wide array of models developed to capture the structural and functional complexities of the respiratory system—propelled by increased computational power, the developments in medical imaging (e.g., CT and MRI) and experimental techniques, and the emergence of efficient numerical methods for solving ordinary and partial differential equations.^250^ In particular, CFD has been extensively applied to investigate a range of respiratory airflow phenomena while taking into account key factors such as turbulence, temperature and humidity, fluid-structure interactions, and aerosol transport and deposition. For example, based on the accurate geometry of the lower respiratory tract derived from medical imaging techniques, Ou et al. recently performed CFD simulations of the entire lower airway from generations G0-G23 and provided quantitative insight into the respiratory mechanics under spontaneous respiration, including the airflow distribution, the internal flow dynamics, and the tissue-level mechanical characteristics.^251^ Their model establishes an important platform for exploring biomechanical phenomena such as force-induced cellular morphogenesis and differentiation. To study the effect of the respiratory wall movement on the airflow through the respiratory tract, Emmerling et al. simulated a complete breathing cycle incorporating dynamically moving glottis walls and resolved intricate turbulent flow structures using a hybrid Reynolds-averaged Navier-Stokes/large eddy simulation (RANS-LES) method, i.e., the stress-blended eddy simulation (SBES) approach.^252^ They found that glottis motion imposes a profound impact on airflow structures throughout the entire breathing cycle, with marked variations in both flow patterns and velocities. These findings underscore the need for future respiratory airflow studies to incorporate dynamically moving airway walls in order to accurately capture the detailed flow structures. In addition, computational models have been increasingly utilized to elucidate the pathogenesis of respiratory diseases.^253^^,^^254^ For example, Tsega and Katiyar constructed four airway models to investigate the narrowing effects of asthma on inspiratory airflow dynamics.^254^ Inspiratory airflow velocity, wall pressure, and wall shear stress were found to be elevated in asthmatic airways compared to healthy counterparts, with more pronounced differences in flow patterns observed in upper airway generations. These findings offer quantitative insights into airflow dynamics during an asthma attack. With the rapid advancement of mechanical modeling and simulation techniques, computational models are now closer than ever to being used in clinical practice.^250^ In particular, patient-specific models have garnered increasing attention due to their ability to capture the individual anatomical and physiological variations,^255^^,^^256^ thereby supporting a more tailored approach for personalized diagnosis and intervention. The strategic integration of AI techniques into the CFD computational framework has opened new possibilities for efficient airflow modeling.^257^ Recently, Talaat et al. developed a PINN-CFD framework for 3D respiratory flow simulations that reduces meshing burden and computational cost while maintaining predictive fidelity. It is worth noting that, despite these advantages, training efficiency and scalability remain significant challenges.^258^

#### Mechanobiological studies of the lung

Mechanobiological studies have provided enlightening insight into how mechanical cues drive respiratory diseases. Take asthma as an example. This respiratory disease is diagnostically characterized by bronchoconstriction, and its pathogenesis has traditionally been attributed primarily to inflammation. Recent findings indicate that the mechanical constriction can trigger inflammation through pathological epithelial extrusion.^259^ During bronchoconstriction, contraction of airway smooth muscle (ASM) leads to severe epithelial crowding, which activates a conserved mechanotransduction cascade. More specifically, pathological compression activates stretch-activated channels (SACs; e.g., Piezo1) and TRP channels (TRPV1/TRPM8), leading to the production of sphingosine-1-phosphate (S1P). S1P subsequently binds to S1P_2_ receptors, triggering Rho-mediated actomyosin contraction that drives apical extrusion of live epithelial cells.^259^^,^^260^ Excessive extrusion denudes the epithelium, compromising both its physical integrity and immunological barrier function. This exposes the underlying tissue to pathogens and inflammatory mediators, thereby triggering immune cell infiltration, including eosinophils and neutrophils. Notably, standard bronchodilators (e.g., albuterol) relax ASM but do not prevent epithelial extrusion or detachment. In addition, bronchoconstriction has also been shown to mechanically induce mucus hypersecretion through calcium-dependent signaling pathways.^259^^,^^261^ Inhibiting extrusion upstream using SAC/TRP blockers (Gd^3+^) or S1P antagonists (SKI-II or JTE-013) preserves epithelial integrity, suppresses inflammation, and mitigates mucus overproduction, offering a mechanistically informed approach to disease modification.

Aberrant contraction of ASM cells (ASMCs) is another pathogenic driver of bronchospasm and asthma. Although β-adrenergic receptor agonists remain the mainstay of bronchodilator therapy in bronchial asthma, their clinical utility is often constrained by adverse effects. Among emerging alternatives, bitter taste receptor (TAS2R)-targeting bronchodilators have gained increasing attention. It has been revealed that TAS2R14 activation in ASMCs by bitter substances can trigger endoplasmic reticulum Ca^2+^ release, stimulating big potassium (BK) channels to reduce ASM contractility and normalize pathological stiffness.^262^^,^^263^ Most existing screening strategies for bitter compounds rely heavily on intracellular signaling readouts (e.g., calcium flux), but may suffer from limited efficiency or frequent false negatives. To address this issue, cellular stiffness has been recently leveraged as a functionally relevant biophysical readout for identifying candidate TAS2R agonists. Through this biomechanical screening approach, flufenamic acid (FFA) was identified as one of the most potent TAS2R14 agonists.^264^ Interestingly, it has been revealed that FFA not only elicits potent relaxation of ASMCs but also suppresses proinflammatory cytokine release, positioning it as a promising dual-action therapeutic candidate targeting both bronchoconstriction and inflammation in asthma.

In addition, pathological tissue stiffness in asthma has been shown to hyperactivate mechanosensitive ion channels (e.g., Piezo), which are closely associated with ASM migration, adhesion, and contractility.^265^ Targeting these channels pharmacologically may therefore alleviate downstream mechanical dysfunctions and offer a promising strategy for asthma therapy. Among these, Piezo1 has attracted particular interest. Accumulating evidence suggests that modulating Piezo1 in ASMCs offers dual therapeutic benefits in asthma therapy by both preventing bronchoconstriction and reversing established airway narrowing. Specifically, activation of Piezo1 with selective agonists (e.g., Yoda1) has been shown to inhibit the initiation of contractile responses^266^ and rapidly relax precontracted ASMCs.^267^

In IPF, cells within the fibrotic lung, including fibroblasts and alveolar epithelial cells, sense and respond to increased stiffness through the Piezo1-RhoA/ROCK-YAP/TAZ signaling pathway. In the nucleus, YAP/TAZ initiates a pro-fibrogenic gene expression program, upregulating the production of α-SMA and ECM components, including collagen.^268^ This creates a self-perpetuating vicious cycle: matrix stiffening activates fibroblasts to produce more ECM, which further increases stiffness, perpetuating the fibrotic process.^269^ Preclinical studies demonstrate that inhibiting Piezo1, either genetically or pharmacologically with tools such as the peptide inhibitor GsMTx4, can effectively blunt the pro-fibrotic response.^270^ More recently, to elucidate how matrix stiffness influences the epigenetic state to regulate transcription, Cosgrove et al. identified a class of mechanosensitive genomic enhancers that potentiate the cellular response to matrix stiffness. Meanwhile, their results indicate that epigenetic editing of mechanoenhancers can regulate the activation of disease-associated genes in lung fibroblasts, offering potential for precise intervention in mechanically driven diseases.^271^

#### Future remarks

Future studies should aim to comprehensively characterize the multiscale mechanical behaviors of the lung and its associated structures in both healthy and diseased states and to uncover how these tissues and resident cells sense and respond to diverse physiological and pathological mechanical stimuli. In particular, emphasis should be placed on characterizing key mechanical properties—such as elasticity, viscoelasticity, and anisotropy—across lung tissues and on elucidating the mechanisms of mechanosensation and mechanotransduction in diverse resident cell types. In particular, investigating the force-dependent interactions of mechanosensitive proteins, such as integrins, cadherins, selectins, and cytoskeletal proteins, will deepen our understanding of how cells sense and respond to mechanical cues. Meanwhile, substantial efforts should be directed toward translating these findings into clinical innovations that can transform respiratory care. A key priority is to standardize quantitative biomarkers at the molecular, cellular, and tissue levels to enable reliable early detection of pathological processes such as fibrosis and remodeling. Therapeutically, substantial challenges persist in translating mechanobiological discoveries into targeted therapies. The ubiquitous nature of many mechanosensitive targets, including Piezo channels, necessitates developing lung-specific delivery systems to minimize off-target effects. It is essential to validate the universality of well-established mechano-targeted interventions in specific respiratory diseases and to explore their potential applicability across a broader spectrum of respiratory pathologies. Achieving these goals will require advances in methodology, technology, and computational modeling, and AI might make significant contributions to these areas. Fully harnessing advances in lung mechanics and mechanobiology will undoubtedly enhance respiratory diagnosis and therapeutics.

### Craniomandibular system

**Figure 6:**
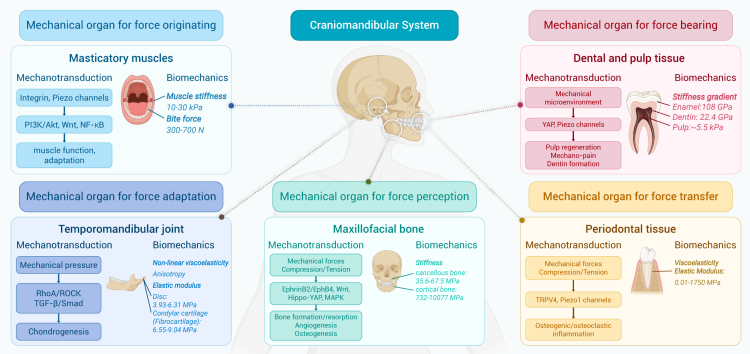
Biomechanics and mechanobiology in the craniomandibular system

The craniomandibular system consists of muscles (including masticatory, tongue, palatal, and hyoid muscles), tooth and periodontal tissue, the upper and lower jaw, and the temporomandibular joint (TMJ) (Figure 6). All components cooperate closely in the generation and conduction of biting force and jointly safeguard the essential life function of mastication.^272^ Therefore, “force” is widely permeated in the biological basis and clinical application of various disciplines of stomatology,^273^ such as orthodontics,^274^ prosthodontics,^275^ periodontology,^276^ implantology,^277^ and occlusion.^278^ In-depth understanding of dental biomechanics and mechanobiology helps to provide an important guarantee for dental treatment. The bones, joints, and ligaments involved in the craniomandibular system exhibit unique characteristics. The alveolar bone (AB), which forms the tooth-supporting structure, is a highly vascularized trabecular bone with a considerable capacity for remodeling,^279^ distinguishing it from the long bones discussed in section bone and joints. Also different from hyaline cartilage in large joints, TMJ condylar cartilage belongs to fibrocartilage and has a dense prechondroblastic layer with strong proliferation potential, which is the structural basis for TMJ maintaining strong lifelong remodeling ability. Unlike the predominantly collagenous and relatively avascular ligaments found elsewhere in the body (e.g., knee or ankle ligaments), the periodontal ligament (PDL) surrounding the tooth root is uniquely characterized by its high cellularity, rich vascularization, and remarkable capacity for continuous remodeling. These structural differences are critical to understanding the unique biomechanics and mechanobiology of the craniomandibular system.

#### Biomechanical and mechanobiological studies of masticatory muscles

The masticatory muscles consist of four main groups: masseter, temporalis, medial pterygoid, and lateral pterygoid. They facilitate mandibular movement essential for chewing, speaking, and swallowing. These muscles generate forces that drive mandibular motion to create the so-called bite force, stimulating the teeth and ABs, periodontal tissues, and TMJ. The biomechanical properties of masticatory muscles reflect their function and health, aiding in disease diagnosis and treatment. Bite force, a key force source in the system, ranges from 300 to 700 N in adults, with higher values in males.^280^ Conditions such as muscle atrophy, aging, neurological disorders (e.g., facial palsy), or craniofacial deformities (notably high-angle malocclusion) can reduce bite force.^281^ In high-angle cases, characterized by a steep mandibular plane angle (>28°), reduced masticatory muscle efficiency and shorter mandibular ramus height compromise force generation during occlusion.^282^ Muscle stiffness, measurable by elastography or hardness meters, typically falls between 10,000 and 30,000 N/m^2^, depending on muscle state.^283^^,^^284^ Disorders such as myofascial pain syndrome or nerve damage alter stiffness significantly. Mechanotransduction in masticatory muscles involves integrin-mediated signaling, mechanosensitive channels such as Piezo1/2, and pathways such as PI3K/Akt, Wnt, and NF-κB.^285^^,^^286^^,^^287^^,^^288^ These mechanotransductions regulate cytoskeletal remodeling, proliferation, and differentiation and are crucial for understanding muscle dysfunction and developing therapeutic strategies.

#### Biomechanical and mechanobiological studies of tooth tissue

As the primary biomechanical organ responsible for bite force bearing, teeth can withstand exceptionally high chewing and biting forces for decades without sustaining damage, underscoring their distinctive and superior biomechanical properties. There exists a pronounced gradient distribution of mechanical performance across the various layers of teeth, from the surface to the interior. Specifically, in terms of elastic modulus, values range from 108 GPa in enamel to 22.4 GPa in dentin and approximately 5.5 kPa in dental pulp.^289^ This biomechanical complexity is related to component differences and hierarchical arrangement, which play a key role in the physiological processes of teeth.

As the hardest structure in mammals, enamel, the outermost structure of a tooth crown, has a variety of distinct mechanical properties such as stiffness, viscoelasticity, strength, and toughness.^290^ Studying the relationship between the biomechanical properties and the microstructure of teeth provides a basic basis for the design of high-performance biomimetic materials. Some achievements have been made in the field of designing biomimetic mineralization systems to promote the self-healing of demineralized teeth for the prevention and treatment of dental caries^291^ and the *in vitro* synthesis of multi-layer and high-mechanical-property biomimetic materials.^289^^,^^292^ In the future, it remains a great challenge to develop high-performance materials for dental hard tissue repair under physiological conditions to extend the lifespan of teeth.^293^

Dental pulp is the loose connective tissue at the very center of the tooth, which is rich in blood vessels and nerves. In pathological conditions such as irreversible pulpitis, the dental pulp ECM undergoes significant remodeling characterized by increased collagen deposition and cross-linking, leading to elevated tissue stiffness and altered viscoelastic properties. These changes disrupt the normal mechanotransduction pathways (e.g., Wnt signaling) that are crucial for pulp homeostasis and repair, ultimately contributing to cellular dysfunction and necrosis.^294^^,^^295^ Recent research reveals that aging significantly alters the mechanical microenvironment of dental pulp, leading to reduced stiffness and slower stress relaxation (i.e., degraded viscoelasticity), which impairs regeneration capacity.^296^ In recent years, the development of cell culture systems based on simulating the *in vivo* physical environment (such as 3D culture, nano/micrometer topology, layered structure, and matrix mechanical properties) has been an area of research interest.^297^^,^^298^ Notably, biomimetic hydrogels engineered to replicate the “Young-Mechanical Niche” (characterized by high stiffness and rapid stress relaxation akin to young dental pulp) have demonstrated exceptional efficacy.^296^ It is believed that tissue engineering scaffold materials (such as functional hydrogels, microspheres, and acellular matrix) that mimic the mechanical properties of dental pulp tissue can promote cell proliferation, migration, and directional differentiation, ensure the secretion of ECM, and achieve the best regeneration of dental pulp tissue.^299^^,^^300^ Specifically, studies show that stiffness-dominated mechanotransduction through the YAP/TEAD1/CTGF/Cyr61 pathway is critical for enhancing human dental pulp stem cells (hDPSCs) odontogenic differentiation and neovascularization *in vivo*, with stiffness exerting a more dominant effect than viscoelasticity alone.^296^ The mechanobiological mechanisms need to be further studied. Mechanical stimulation of dental pulp stem cells to promote extracellular vesicle production and optimize their function for dental pulp regeneration is also worthy of future exploration.^301^

Many researchers have focused on the role of the newly discovered mechanosensing receptor, the Piezo ion channel, in the mechanosensing of odontoblasts and other dental pulp cells.^302^^,^^303^ Studies have shown that Piezo1 and Piezo2 regulate mechanical pain responses, inflammatory responses, dentin development, and (re)mineralization in a mechanosensitive manner.^303^^,^^304^ The clarification of the mechanobiological mechanism provides a theoretical basis for the design of drug targets to treat dental pulp diseases such as toothache and dentin hypersensitivity and offers novel strategies for dental pulp regeneration.

#### Biomechanical and mechanobiological studies of periodontal tissue

The PDL, a viscoelastic connective tissue, connects teeth to the AB, buffering occlusal forces, protecting tissues, and regulating remodeling.^305^ *In vivo* studies in pigs reveal that mastication induces complex tooth mobility: maxillary molars primarily exhibit buccal tipping and intrusion (average displacement: 192 ± 95 μm), with concurrent compression of buccal AB and elevated PDL fluid pressure (3.63 ± 0.80 kPa).^306^ Its elastic modulus varies significantly (0.01–1,750 MPa), owing to sampling difficulties and diverse root morphologies, complicating biomimetic PDL reconstruction.^307^ In periodontitis, the inflammatory process leads to the degradation of the PDL’s ECM, particularly the principal collagen fibers, resulting in a significant reduction in tissue tensile strength and viscoelasticity. This pathological softening of the PDL disrupts the normal transmission of occlusal forces, exacerbates AB resorption, and impairs the mechanosensing capabilities of periodontal cells (e.g., PDL stem cells [PDLSCs]), thereby hindering their regenerative potential.^308^ The balance between pathological damage and regenerative stimulation is a core concept in periodontal mechanobiology.

PDL cell membrane mechanosensors, including cilia, integrins, and calcium channels (TRPV, Piezo, and G-protein-coupled receptor [GPCRs]), respond to mechanical stimuli. Notably, functional tooth mobility generates spatially heterogeneous strain patterns (buccal compression > palatal compression), which may locally modulate mechanosensor activity. TRPV4 and Piezo1 are key players. Compression, tension (stretching), and shear strains (forces) increase TRPV4 expression, calcium influx, and receptor activator of NF-κB ligand (RANKL) production, with higher TRPV4 expression observed on the compression side of the tooth during orthodontic tooth movements.^309^ Piezo1, which responds to mechanical tension and compression, shows increased expression under orthodontic loading,^310^ potentially sharing pathways with TRPV4. The *in vivo* correlation between occlusal force magnitude and PDL pressure amplitude suggests that fluid-mediated mechanotransduction merits further study. Additional research is needed to clarify their distinct roles in bone formation and resorption. Recently, Bae et al. studied the impact of mechanical cues on the epigenetics of PDL cells. Their findings showed that mechanical stretching modulates gene sets involved in mechanotransduction, histone modification, reactive oxygen species metabolism, and differentiation. These results provide evidence that mechanical forces can drive mechanotransductive and epigenetic responses, ultimately shaping gene expression and diverse cellular behaviors.^311^

It has been revealed that PDLSCs can be activated on both the pressure side (compressive force) and the tension side (stretch force): compressive force promotes osteoclast differentiation by upregulating the RANKL/osteoprotegerin (OPG) ratio and activating TGF-β/Smad and Wnt/β-catenin pathways. Stretch (12% strain) induces osteogenic differentiation through the MAPK pathway, but excessive stretch (>12%) can promote the secretion of inflammatory factors.^312^ Meanwhile, matrix stiffness has recently been shown to be positively associated with global DNA methylation and influences osteogenic differentiation via such epigenetic mechanisms in PDLSCs, providing insights into the regulation of orthodontic tooth movement.^313^ Periodontal ligament fibroblasts (PDLFs) are subjected to cell-cycle arrest (G1 phase) under compressive force (2 g/cm^2^), and proliferation-related proteins such as MCM2 and PCNA are downregulated, while the apoptosis rate is increased.^314^ Heavy force (60 cN) induces Bax/caspase-3 expression more significantly than light force (10 cN).^315^ In addition, mechanical force regulates PDL cell autophagy, secretion of inflammatory factors such as IL-6 and IL-8, and macrophage polarization through Piezo1/TRPV4 ion channels and YAP signaling.^274^^,^^316^ Recent studies have demonstrated that mechano-growth factor (MGF) is upregulated in response to occlusal loading and promotes PDL regeneration by activating Fyn-FAK and Fyn-RhoA-YAP signaling pathways, thereby driving PDLSC differentiation toward a fibroblastic phenotype. This mechanochemical coupling underscores MGF as a key mediator of periodontal responses to biomechanical stimuli.^317^^,^^318^

#### Biomechanical and mechanobiological studies of maxillofacial bone

The jawbone comprises dense cortical bone (732-10,077 MPa)^319^ and porous cancellous bone (35.6–67.5 MPa),^320^ which differ significantly from the mechanical properties of the long bones. In contrast to the long bones located in other parts of the body, the AB is cancellous bone that surrounds the roots of the teeth. Due to its rich blood supply, the AB has a high capacity for remodeling.^321^ Teeth and jaws interact closely, with tooth development, arrangement, and occlusal force shaping jawbone growth, morphology, and function.^322^ Occlusal force stabilizes jawbone structure, and its reduction, such as that due to tooth loss, leads to alveolar ridge resorption and decreased bone density, impacting mastication efficiency and facial aesthetics.

Orthodontic success relies on the balance between bone formation and resorption, regulated by osteoblasts, osteoclasts, and signaling pathways such as EphrinB2/EphB4, MAPK, and Wnt.^274^ PGE2 plays a dual role: low levels promote osteoblast activity, while high levels stimulate osteoclast differentiation, leading to bone resorption.^323^ The Hippo-YAP pathway, involving YAP/TAZ, responds to cell stretching and cytoskeletal changes, regulating bone remodeling.^324^ Mechanical stimulation also activates MAPK cascades (ERK1/2, p38, and c-Jun N-terminal kinases [JNKs]), contributing to AB adaptation during orthodontic treatment.^325^ Understanding the dynamic interplay between PDL and AB under mechanical forces is essential for effective jaw remodeling.

Research in oral implantology focuses on improving osseointegration and reducing stress concentration through optimized implant designs and surface treatments. Biochemical and physical methods, such as calcium phosphate coatings, electrodeposition, and layer-by-layer assembly, enhance implant stability and promote long-term success.^326^ Finite element analysis aids in designing implants that mimic natural PDL structures, optimizing dimensions, thread morphology, and material gradients to match surrounding bone conditions.^327^

#### Biomechanical and mechanobiological studies of the TMJ

As the only joint in the craniomaxillofacial region, the TMJ, consisting of the condyle, articular disc, glenoid fossa, joint capsule, and ligaments, supports complex multi-directional movements for chewing, speaking, and swallowing.^328^ It distributes occlusal forces across its structures, with the condyle adapting to mechanical loads through cartilage with self-repair capacity and subchondral bone remodeling.^329^ The articular disc reduces friction and absorbs shock with high elasticity, while ligaments stabilize the joint and prevent excessive displacement. In contrast to joints in other parts of the body, TMJ cartilage is secondary cartilage, i.e. fibrocartilage, whereas other articular cartilage is primary cartilage, i.e. hyaline cartilage. There are significant differences between the two cartilages in terms of structural composition and mechanical properties. Under pathological conditions such as osteoarthritis, cartilage elasticity, viscoelasticity, compressive strength, and fatigue resistance decline, leading to impaired function.^330^^,^^331^ Mechanotransduction pathways, including RhoA/ROCK, MAPK/ERK, and TGF-β/Smad, regulate cartilage remodeling, proliferation, and matrix synthesis.^332^^,^^333^^,^^334^ However, abnormal stresses can cause calcification via extracellular vesicles, increasing cartilage hardness and brittleness. This loss of elasticity accelerates TMJ osteoarthritis progression, exacerbating dysfunction and pain.

#### Future remarks

The concept of “force” is widely permeated in the biological basis of various disciplines of stomatology. In the craniomaxillofacial system, the masticatory muscles, teeth, periodontal tissues, upper and lower jaw bones, and TMJ work together to generate, transmit, and adapt occlusal force. Occlusal force acts as the primary driving force for the development, reconstruction, and remodeling of each component of the craniomandibular system.^278^ Force transmission and regulation are essential for masticatory function, stability of facial structures, and health of teeth.^335^ Biomechanics helps us to understand how forces affect the shape and function of teeth and joints, thereby guiding treatments in fields such as orthodontics, prosthodontics, and oral surgery. Mechanobiology further reveals how these internal and external forces trigger cellular and molecular responses, such as cell proliferation, differentiation, and gene expression, which are crucial for understanding the self-repair, adaptation, and long-term maintenance capabilities of the craniomandibular system.^336^ Mechanobiological approaches are essential for achieving a deeper and more precise understanding of the anatomy and physiology of the craniomandibular system. However, the theoretical development and technical application of these approaches in the treatment of various oral and craniomandibular diseases remain relatively limited. Meanwhile, the current understanding of the biomechanical homeostasis within this system remains inadequate. If we could quantitatively regulate biomechanical homeostasis *in vivo*, it would open up entirely new therapeutic strategies for disease management. Moreover, investigating the force-dependent interactions of mechanosensitive proteins, such as integrins, cadherins, selectins, and cytoskeletal proteins, in the craniomandibular system will provide new insights into how mechanical cues regulate cellular behavior, orchestrate tissue remodeling, and influence disease progression. In addition, future research should focus on developing quantitative and theoretical models. Such models can not only predict the behavior of molecules, cells, and tissues, thereby enhancing our understanding of physiological and pathological processes, but also offer quantitative guidance for the intervention of pathological processes, thus opening new avenues for maintaining oral and craniomandibular health and treating their diseases.

### Cancer

**Figure 7:**
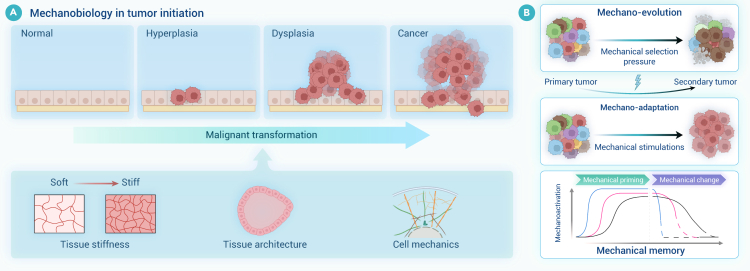
Biomechanics and mechanobiology in cancer

While genetic mutations and epigenetic alterations are essential in tumorigenesis, recent studies reveal that cell-extrinsic mechanical factors (tissue stiffness, solid stress, interstitial fluid pressure (IFP), shear force, geometric confinement, and architecture) within the tumor microenvironment (TME) and the mechanical properties of cells (cell stiffness, contractility, membrane tension, and nuclear mechanics) also play pivotal roles.^337^^,^^338^^,^^339^ These mechanical cues influence not only tumor initiation and growth but also multiple steps of the metastatic cascade, such as invasion, intravasation, dissemination via circulation, extravasation, and colonization, as well as the responses to chemotherapy and immunotherapy (Figure 7). Additionally, mechano-adaptation and mechano-evolution, the processes by which cancer cells adapt to mechanical stresses over time, provide a new mechanistic explanation for various metastatic outcomes. The integration of mechanobiology into cancer research has provided profound insights into how mechanical forces (both external and internal) regulate tumor initiation and progression.^340^^,^^341^^,^^342^^,^^343^ By focusing on how these mechanical factors contribute to key steps in cancer progression, previous studies redefine our understanding of cancer development by establishing its mechanical framework and offer the potential for novel therapeutic interventions aimed at targeting mechanical cues and signaling. Here, we summarize recent advances in biomechanics and mechanobiology in cancer research, which provide new insights into the roles of mechanics in cancer and shape the trajectory of future research and applications in this field.

#### Tumor initiation

In normal tissues, the ECM provides structural support and orchestrates multiscale cellular behavior. During tumor progression, the stiffness of ECM is increased mainly due to enhanced collagen deposition and cross-linking. Of note, even a large body of evidence demonstrates that tumor progression leads to increased ECM stiffness. As a feedback loop, ECM stiffness is not just a consequence of cancer initiation and progression but also actively facilitates these processes. Tissue stiffness has been associated with increased risk of various cancers, including but not limited to breast, colon, prostate, gastric, and liver cancer.^209^^,^^344^^,^^345^^,^^346^ Preceding the ECM remodeling driven by transformed cells, several pathological conditions, including chronic inflammation, aging, and tissue injury, can elevate the stiffness of the ECM, predisposing the tissue to a tumor-susceptible state.^347^ ECM stiffening plays a key role in promoting the susceptibility of healthy cells to oncogenic transformation and protects transformed cells against epithelial defense, leading to tumorigenesis.^345^^,^^346^^,^^348^^,^^349^

Tissue architecture constraint has been shown to arrest oncogenic growth, maintain homeostasis, and even eliminate transformed cells.^350^ On the other hand, tissue architecture is dramatically altered^351^ and can serve as a fundamental determinant of tumorigenesis and tumor morphogenesis.^352^^,^^353^^,^^354^ Local mechanical tension induced by cellular geometry enhances the stem cell marker expression and modulates cancer cell tumorigenicity.^355^ Nonetheless, it remains uncertain whether tissue architecture and subsequent local force imbalances lead to spatial vulnerability to oncogenic transformation.

Cancer cells are generally softer and more contractile than their benign counterparts, and cell stiffness is usually negatively correlated with malignancy.^340^^,^^356^ Interestingly, the change in cell stiffness is not monotonic but dynamic during oncogenic transformation. Following oncogene activation, cells initially exhibit increased stiffness and enforced actin cytoskeleton transiently and then gradually transition to a softer state compared to their non-transformed counterparts.^357^^,^^358^ Although the pathological relevance of this fluctuating stiffness remains ambiguous, it could potentially signify a phenotypic alteration due to modifications in the cytoskeleton or cell contractility during multiple transformation phases. Of note, targeting cell mechanics, including the cytoskeleton and contractility, could be a promising way to stop the transformation during oncogene activation.^345^^,^^357^^,^^359^ However, its clinical application remains challenging due to the difficulty in determining the optimal therapeutic window. The role of cell mechanics in the susceptibility to oncogenic transformation still needs to be further investigated.

#### Mechano-evolution, mechano-adaptation, and mechanical memory

The concepts of mechano-selection, mechano-adaptation, and mechanical memory have emerged as key frameworks for understanding how tumor cells fit into a dynamically evolving and even hostile mechanical microenvironment during tumor growth and metastasis.

Mechano-evolution refers to the process by which heterogeneous cancer cells undergo clonal selection based on their ability to withstand different mechanical cues in the TME. Cell mechanical competition for space within the TME could favor the outgrowth of more aggressive clonal subpopulations, thereby enhancing the overall fitness and sustainable growth of the tumor.^360^ Mechanical deformation empowers a subpopulation of tumor cells with the ability to resist cell death induced by mechanical squeezing, resulting in a more proliferative and chemotherapy-resistant phenotype.^361^ Matrix softness acts as a novel selection stress to enrich the fittest variants of cancer cells with specific mechanical and aggressive traits on soft matrix.^362^ Growing evidence suggests that mechanical forces act as another type of natural selection, contributing to tumor heterogeneity and the emergence of highly metastatic and mechanically resilient subpopulations.

Mechano-adaptation describes the resilient ability of cancer cells to undergo phenotypic changes in response to diverse mechanical stimuli in a time-dependent manner. This plasticity allows tumor cells to dynamically adjust their behavior to match the mechanical microenvironment of their surroundings. One of the key mechanisms underlying mechano-adaptation is the dynamic change of cell mechanics, which enables cancer cells to alter their shape, stiffness, and contractility in response to external mechanical stimuli—a topic that has been well reviewed elsewhere.^363^ In addition, mechano-adaptation also involves alterations in mechanotransduction signaling pathways and epigenetic modifications. Mechanosensitive pathways, such as YAP/TAZ, runt-related transcription factor 2 (RUNX2), and myocardin-related transcription factor-A (MRTF-A)-related signaling, are activated in response to mechanical stimuli, driving adaptive changes that confer cancer cell survival advantages at different metastatic stages, including enhancing metastatic potential and overcoming the hostile microenvironment-mediated cell death in distant organs.^364^^,^^365^^,^^366^ Emerging evidence indicates that epigenetic regulation could serve as one pivotal mechanism for mechano-adaptation.^367^ The soft microenvironment in the brain activates the pro-survival signals and induces the dormancy of tumor cells via DNA methyltransferase 1 (DNMT1) inhibition.^368^ A recent study showed that genetic variation can drive cancer cell adaptation to ECM stiffness, providing direct evidence of selection at the genetic level.^362^ These mechanisms enable cancer cells to sense and respond to multiplexed mechanical cues, allowing them to adapt to the evolving mechanical landscape of the TME.

Mechanical memory represents the ability of cells to “remember” past mechanical imprints, which influence their subsequent responses to similar or other types of mechanical stimuli. This phenomenon has been widely reported in fibroblasts, mesenchymal stem cells (MSCs), and epithelial cells.^369^ However, there are relatively limited studies showing cellular mechanical memory in the realm of cancer research.^369^ For example, priming cancer cells on fibrotic-like matrix with elevated stiffness induces a bone metastatic phenotype, which can be maintained for a long period even on soft matrix,^366^ while persistent priming in local soft niches within the primary tumor promotes the dissemination and colonization of breast cancer cells to soft brain tissue.^370^ These findings indicate that the traits emerging after mechanical stimulation in the primary tumor or during the metastatic journey could be retained to instruct the outcome or responses at later stages of metastasis. Emerging evidence indicates that cancer cell memory is governed by a network of mechanobiological pathways that shape diverse cellular behaviors. Mechanical cues within the tumor microenvironment, such as matrix stiffness gradients and tensile and compressive forces, can influence long-lasting transcriptional, epigenetic, and phenotypic changes in cancer cells.^371^ Understanding the mechanisms underlying mechanical memory could provide new therapeutic targets for metastasis prevention by disrupting the retention of pro-metastatic mechanical adaptations.

#### Future remarks

Considerable progress has been made in dissecting the dramatic changes in the mechanics of TME and tumor cells and in elucidating the significance of these mechanical cues in tumor progression. Nevertheless, the roles of biomechanics and mechanobiology in cancer remain far from being well understood, especially in the following aspects. First, how tissue and cell mechanics impact cancer initiation is yet to be deciphered. Conventionally, cancer has been believed to arise from an accumulation of multiple mutations. Advanced techniques, such as second-generation sequencing technologies, have been adopted to discover many mutated cells in healthy tissues and differential cancer initiation rates in different tissues, implicating the potential roles of tissue microenvironment and intrinsic cell properties. Second, the mechanical heterogeneity of the TME and its implication in tumor metastasis remain poorly understood. Tumor tissue has been believed to become uniformly stiffened as a whole, which significantly affects the malignancy of tumor cells. However, as a hallmark of cancer, genetic and phenotypic heterogeneity could lead to considerable spatial variations in the mechanics of the TME, which are expected to trigger critical effects on various malignant functions, such as the tropism of tumor cells to metastasize to specific organs and the immunogenic and metabolic responses of tumor cells. Third, the combinatorial effects of mechanotransduction and biochemical signaling on tumor progression remain unclear. The mechanism by which tumor cells respond to mechanical cues is known to crosstalk with well-investigated oncogenic signaling, both of which may function either synergistically or counteractively in promoting cancer progression. Fourth, a variety of mechanosensitive molecules, including integrins, cadherins, selectins, and cytoskeletal proteins, exhibit force-dependent interactions. Further in-depth investigation of molecular interaction and force-dependent mechanotransduction mechanisms will help clarify how cancer cells sense, respond to, and adapt within their mechanical microenvironment and reveal the mechanisms driving invasion and metastatic progression. Further, despite progress in the fundamental understanding of mechanical factors in cancer, the translation of this knowledge into clinical therapy remains in its early stages. Addressing these burning issues can provide new evidence to strengthen the importance of biomechanics in cancer and support a new notion that cancer may not only be a genetic but also a “mechanical” disease.

### Mechanoimmunology

**Figure 8:**
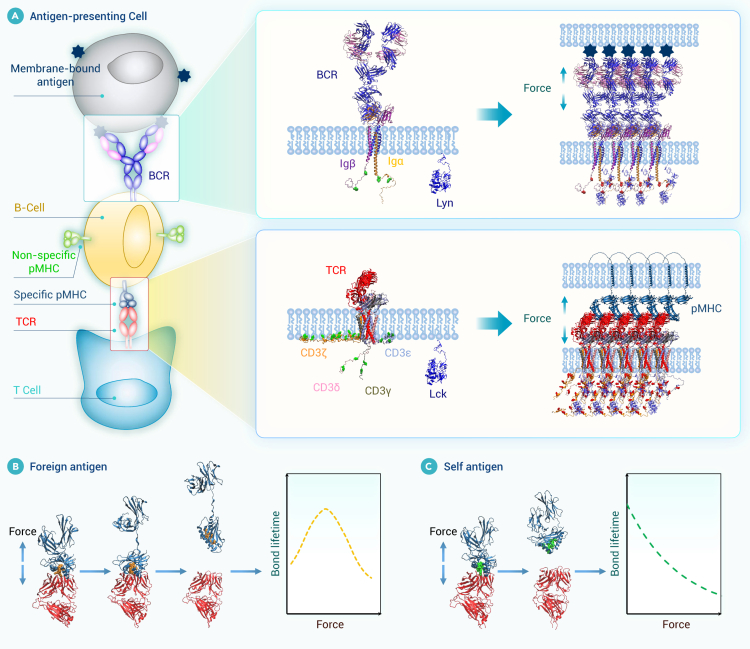
Biomechanics and mechanobiology in immunology

The immune system relies on precise molecular interactions to recognize and respond to foreign pathogens while avoiding overt responses to self-antigens. Traditionally, immune recognition has been studied as a purely biochemical process involving receptor-ligand binding, signal transduction, and activation cascades.^372^^,^^373^^,^^374^^,^^375^^,^^376^^,^^377^ However, a growing body of evidence demonstrates that mechanical forces also play a crucial role in regulating immune receptor signaling and function.^378^^,^^379^ This emerging field, known as mechanoimmunology, investigates how immune receptors such as T cell receptors (TCRs) and B cell receptors (BCRs) sense and respond to mechanical cues (Figure 8), which affect antigen recognition, signaling thresholds, and, ultimately, immune activation.^380^^,^^381^ The field of mechanoimmunology complements traditional immunology by adding a critical biomechanical axis to our understanding of immune regulation. It emphasizes the dynamic interplay between physical and biochemical cues in shaping immune responses.

#### Mechanosensitivity of immune receptors

TCRs (and probably also BCRs) are structurally suited to sense mechanical forces during interactions with antigen-presenting cells (APCs). Mechanical forces enhance the sensitivity and discriminatory power of antigen recognition by these receptors and play a key role in T/B cell responses.

TCRs are expressed on the surface of T cells and interact with peptide-major histocompatibility complex (pMHC) molecules presented by APCs (Figure 8A).^382^^,^^383^^,^^384^^,^^385^ TCR antigen recognition is remarkably sensitive, as T cells can be activated by a single pMHC molecule.^386^ It is also incredibly specific, being able to discriminate between two TCR epitopes with a single amino acid difference.^387^ For example, a single point mutation on the antigenic peptide can convert an agonist to an antagonist, leading to completely different T cell responses. However, TCRs are known to have low affinity for pMHC, with a narrow dynamic range. These early TCR-pMHC binding kinetics data are based on soluble recombinant TCR molecules and measured in a 3D fluid phase with surface plasmon resonance (SPR). Later studies have directly quantified TCR kinetics on live T cells interacting with pMHC on surrogate APCs,^388^^,^^389^^,^^390^^,^^391^ thus better mimicking *in vivo* T cell-APC interactions where both TCRs and pMHCs are anchored on their two-dimensional (2D) surfaces. The 2D TCR-pMHC interactions show accelerated kinetics, and 2D TCR affinity has drastically increased the dynamic range compared with 3D affinity, providing a better explanation for T cell sensitivity and antigen discrimination. T cells are highly mobile cells, and the TCR-pMHC bond will experience a pulling force upon bond formation. Accumulating evidence from recent publications has demonstrated that mechanical force is a crucial factor in regulating TCR-pMHC bond characteristics.^392^^,^^393^ Mechanical force can unfold receptor domains, expose cryptic binding sites, or promote conformational changes that enhance binding stability. Of note, force regulates the TCR-pMHC interaction in a peptide-dependent manner such that it amplifies differences in TCR bond properties for different pMHCs, adding an additional layer of molecular mechanism for T cell antigen discrimination.

Single-molecule force spectroscopy studies have shown that the TCR is a mechanosensor,^394^ and the TCR-pMHC bond is strengthened under force via a catch-bond mechanism,^388^ i.e., force prolongs the binding duration (lifetime) of the TCR-pMHC complex. The TCR forms catch bonds with agonist pMHCs but slip bonds—a typical molecular bond type in which increasing force only shortens the bond lifetime—with antagonist pMHCs. In addition, the activation potency of TCR agonists directly correlates with the strength of TCR-pMHC catch bonds. Therefore, force-elicited TCR-pMHC catch/slip bonds represent a new feature of the TCR bond kinetics on top of the traditional force-free binding properties (e.g., affinity). These catch/slip bond behaviors enable TCRs to amplify small differences in affinity between foreign and self-peptides, thus enhancing antigen discrimination. The recognition of specific pMHC by TCRs triggers intracellular signaling and initiates the assembly of the immunological synapse (IS).^395^^,^^396^^,^^397^ TCR-pMHC binding leads to phosphorylation of intracellular signaling motifs on the CD3 subunits associated with the TCR complex, recruiting various kinases and other signaling molecules that propagate the activation signal and ultimately determine T cell functional outcomes. Consistent with force being a critical parameter in T cell antigen recognition, mere pMHC binding to TCRs is not sufficient to trigger TCR signaling, and T cell activation requires T cells pulling against resistance via TCR-pMHC catch bonds. The TCR catch bond has been shown to dictate thymocyte selection,^398^ T cell activation,^388^ and anti-viral^389^ and anti-tumor responses.^390^

Unlike T cells, B cells directly bind native antigens with their BCRs, capturing them either in solution or on cell surfaces (Figure 8A). B cells generate internal forces through cytoskeletal rearrangements to physically pull on the bound antigen, a process that influences BCR signaling. This force-dependent activation is crucial, as it allows B cells to respond to a broad range of antigens with varying affinities.^399^ Additionally, BCR clustering and signaling are modulated by forces generated through interactions with the actin cytoskeleton and integrins, which stabilize the IS between B cells and APCs (Figure 8A).^400^^,^^401^ Mechanical force has also been shown to be important in antibody affinity maturation by physically testing the antibody-antigen binding strength.^399^ Furthermore, B cells can sense mechanical properties of antigen-presenting surfaces, which regulate their activation, proliferation, class switch, and T cell-independent antibody responses.^402^

#### Mechanisms of immunoreceptor catch bonds

Catch bonds represent a critical aspect of mechanosensitivity in immune receptors.^392^^,^^403^ Intuitively, receptor-ligand bonds weaken and dissociate under increasing force, known as slip bonds.^404^ However, catch bonds exhibit the opposite behavior: the application of a pulling force stabilizes the bond, prolonging receptor-ligand interactions.^405^^,^^406^ Catch bonds are especially beneficial for immune receptors that need to recognize and bind relatively low-affinity antigens to trigger downstream signaling effectively.^407^^,^^408^ Here, we focus primarily on TCR catch bonds. Note that catch bonds have also been identified in PD-1 binding to PD-L1/PD-L2, CD40 binding to CD40L, FcγRIIA binding to IgG Fc, and FcγRIII binding to an anti-CD16 nanobody (C28).^393^

The TCR-pMHC complex is one of the best-studied examples of catch bonds in immune receptors.^388^^,^^389^^,^^407^ In the lower force regime, the application of force enhances the stability of TCR binding to pMHCs, particularly for agonist (foreign) peptides, by inducing conformational changes in the TCR and associated signaling proteins (Figure 8B). This force-induced stabilization enables T cells to prolong interactions with pMHCs, leading to more robust signaling and immune activation. When the force becomes larger than the optimal force at which the bond lifetime reaches its peak, catch bonds will transition into slip bonds (Figure 8B). In contrast, for self- or antagonistic antigens, the TCR only forms a slip bond (Figure 8C). Thus, force helps TCRs to discriminate the specific antigens that correspond to different T cell functional outcomes.

Molecular dynamics simulations and structural studies have suggested that catch bonds in TCRs may result from allosteric changes within the TCR-pMHC binding interface, which optimize the orientation and binding affinity of TCRs to pMHCs under force.^407^ These changes are thought to enhance the specificity of TCR signaling, allowing T cells to respond preferentially to foreign peptides over self-peptides.

The CD8 coreceptor has been observed to regulate the T cell specificity by cooperating nonlinearly with TCR catch bonds.^398^^,^^409^ CD8 coreceptors bind to pMHC at a different interface from TCRs. In the presence of CD8, mechanical force induces sequential conformational changes in both pMHC and CD8 for naturally occurring TCRs with moderate affinity. In contrast, engineered high-affinity TCRs form rigid, tightly bound interfaces with the cognate pMHCs, impeding the force-induced conformational shifts essential for optimal catch-bond formation. Paradoxically, these high-affinity TCRs can still establish moderate catch bonds with non-stimulatory pMHCs from their parental lineages, leading to off-target cross-reactivity and diminished specificity. This nonlinear cooperation underscores the critical role of CD8 in targeting cognate antigens.

Although there is no conclusive evidence to show that BCRs exhibit catch bonds, several studies demonstrated that mechanical forces stabilize their interactions with antigens,^399^^,^^410^ facilitating effective antigen uptake and internalization. BCRs, like TCRs, probably undergo conformational changes under mechanical stress, which promotes signaling and antigen processing. Mechanical regulations in BCRs may play a significant role in affinity maturation, as they allow B cells to selectively interact with high-affinity antigens over low-affinity ones in the germinal centers.

#### Mechanotransduction pathways in T and B cells

In T and B cells, mechanotransduction is facilitated by complex signaling pathways that respond to mechanical cues at the IS. The high-resolution structures of TCR/CD3 complexes^411^^,^^412^^,^^413^^,^^414^^,^^415^ and BCR/Igαβ complexes^416^^,^^417^^,^^418^ provide an in-depth understanding of the cross-membrane signaling of T and B cells.

The TCR complex, including CD3ε, -γ, -δ, and -ζ chain signaling domains, is sensitive to mechanical forces. When force is applied to TCRs during antigen recognition, phosphorylation of the immunoreceptor tyrosine-based activation motifs (ITAMs) on the CD3 chains by Src family kinase Lck is initiated. The formation of phase-separated condensates by CD3ε and Lck amplifies the triggering signal and promotes stronger phosphorylation of CD3 ITAMs.^419^ This phosphorylation leads to recruitment of ZAP-70 and downstream signaling molecules such as LAT, SLP-76, and phospholipase C-γ1 (PLC-γ1), which promote phase-separated condensate formation organized by LAT^420^^,^^421^ and further amplify the T cell activation signal.^422^^,^^423^ The actin cytoskeleton and integrin molecules (such as LFA-1) also play crucial roles in TCR mechanotransduction. Actin polymerization and integrin-mediated adhesion stabilize the IS, allowing T cells to sustain forces on TCRs and enhance signal duration and intensity.

In B cells, mechanotransduction pathways involve the BCR’s interaction with actin filaments and integrins that mediate adhesion to APCs. Upon antigen binding, actin-driven forces are generated at the BCR-antigen complex, which not only enhance antigen binding but also promote BCR clustering and phosphorylation of ITAM motifs on the Igα/Igβ complex. This initiates a signaling cascade involving spleen tyrosine kinase (SYK), B cell linker protein (BLNK), and PLC-γ2, resulting in calcium mobilization and activation of the NF-κB and MAPK pathways.^424^ Mechanotransduction in B cells is essential for antigen processing and presentation, as mechanical forces enhance BCR endocytosis and antigen uptake.^425^ Additionally, force-dependent BCR signaling modulates the selection and affinity maturation of B cells within germinal centers.^426^

#### Implications of mechanoimmunology for immune tolerance

The mechanosensitivity of immune receptors may have profound implications for understanding immune tolerance. By modulating mechanical forces, it may be possible to control immune responses. Immune tolerance is the ability of the immune system to avoid attacking the body’s own cells and proteins, which is critical for preventing autoimmune diseases. T cells, in particular, face the challenge of distinguishing between self- and foreign antigens with high specificity. Mechanical forces may influence tolerance mechanisms, particularly in T cells, where the mechanical stability of TCR interactions with self-antigens could impact autoimmunity.

The ability of TCRs to form catch bonds enables T cells to probe and “test” the stability of pMHCs using force. For self-antigens, the TCR-pMHC interaction typically involves low-affinity and weak slip bonds under force and is not sufficient to trigger an immune response. This mechanism helps to ensure that T cells are selectively activated in response to high-affinity interactions that form catch bonds with foreign antigens, which often produce strong and long-lasting mechanical interactions, amplifying the signal for immune activation.

Additionally, mechanical forces at the IS modulate immune responses by qualitatively differentiating TCR catch bonds with foreign antigens from slip bonds with self-antigens, safeguarding the likelihood of autoimmune reactions. In autoimmune diseases, however, dysregulation of these mechanosensitive pathways could lead to inappropriate activation of T cells in response to self-antigens. Understanding how forces affect TCR signaling thresholds may provide insights into new strategies for restoring tolerance in autoimmune conditions.

In B cells, BCRs use the actin cytoskeleton to pull on antigens, stabilizing interactions that meet a certain affinity threshold. This process ensures that only high-affinity BCR-antigen interactions proceed to full activation, which is essential in preventing low-affinity, potentially autoreactive B cells from initiating an immune response. Mechanical forces generated during BCR engagement aid in the selection and elimination of self-reactive B cells during affinity maturation, particularly in the germinal centers, where B cells undergo selection based on antigen affinity.

#### Future remarks

Mechanical cues profoundly impact immune receptor signaling, fundamentally influencing both the initiation and modulation of immune responses. The application of mechanical forces can alter receptor conformation, change ligand-binding properties, and induce receptor clustering, all of which significantly influence cellular responses. Cells mount proper responses through the detection and application of mechanical force. Future research will be crucial in elucidating how diverse types of mechanical stimuli—such as shear stress, substrate stiffness, and topographical features—affect specific signaling pathways within immune cells. Understanding these dynamics is essential for developing therapies that fine-tune immune responses by targeting mechanosensitive elements, potentially leading to more effective treatments for diseases ranging from autoimmunity to cancer. Advancements in quantification methods are indispensable for gaining detailed insights into the mechanical regulation of immune receptor signaling. Technologies such as single-molecule force spectroscopy, DNA-based force sensors, and genetically encoded force sensors are advancing high-resolution, quantitative assessments of the mechanical forces at molecular and cellular levels. Leveraging these tools facilitates the development of comprehensive, multiscale models of mechanoimmunology, offering unprecedented insights into the interplay between mechanical forces and immune function. In particular, more recent developments of intracellular mechanical sensors enable quantitative measurements of force levels inside the cell,^427^^,^^428^ which may help to determine the complete mechanotransduction pathway from extracellular environments into the cell nucleus, and then the exact mechanisms of how mechanical cues shape immune response can be deciphered. However, there are still significant difficulties in studying how the direction of force affects immune receptors and their signal transduction, which is an important area for future research.

The complexity inherent in the mechanical regulation of immune receptor signaling demands robust mathematical models capable of integrating chemical and physical inputs. These models can simulate dynamic interactions between receptors and ligands, predict outcomes of various mechanical stimuli, and identify key nodes in mechanotransduction networks. By incorporating data from high-throughput experiments and advanced imaging techniques, mathematical models provide unparalleled predictive power. Future efforts should focus on refining these models to enhance accuracy and applicability, guiding experimental design, and optimizing therapeutic strategies. Such models will be instrumental in predicting patient responses to treatments, paving the way for personalized medicine approaches that account for individual variations in mechanoimmunology. Ultimately, these integrated approaches will redefine our understanding of immune system function and open new frontiers in biomedicine. Nevertheless, the field of mechanoimmunology is still in the development stage. Future research also needs to focus on developing better *in vivo* models to study mechanotransduction in immune cells and translating these findings into clinical applications.^429^ Additionally, understanding the interplay between mechanical cues and other environmental factors will be crucial.

## Application of biomechanics and mechanobiology

Developments in biomechanical and mechanobiological studies have fostered extensive information on how mechanical forces influence cellular behavior, tissue homeostasis, and disease progression. These advances have deepened our understanding of the fundamental principles underlying mechanosensation and mechanotransduction and have provided a foundation for translating biomechanical knowledge into diagnostic, therapeutic, and tissue engineering applications. For example, CXL to enhance cornea stiffness has been widely adopted for the clinical treatment of KC, and lowering IOP has always been the primary way of treating glaucoma.^133^^,^^430^ Meanwhile, clinical trials targeting mechanosensors (e.g., integrins) and mechanotransducers (e.g., YAP/TAZ) in multiple diseases are in process.^431^ In this section, we will discuss the application of biomechanics and mechanobiology, focusing on mechanomarker development; cancer, vaccine design, and autoimmune disease; tissue damage repair and functional reconstruction; and deformity correction and functional compensation.

### Mechanomarker development

**Figure 9:**
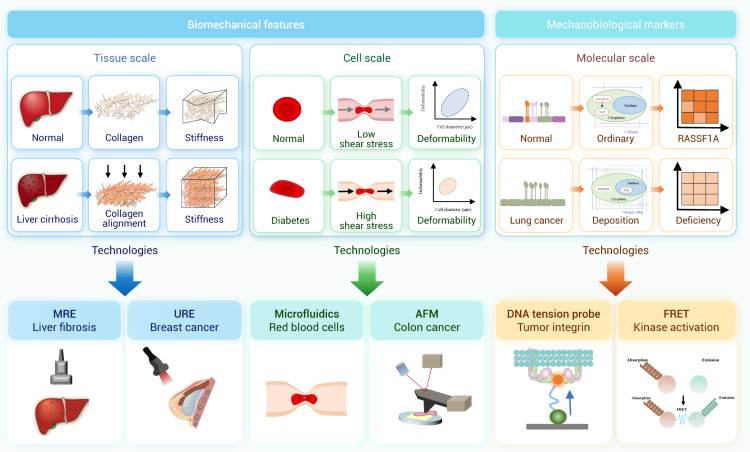
Biomechanical features and mechanobiological markers in disease progression across multiple scales and their corresponding detection techniques

Human beings have long used biomechanical information for disease diagnosis. In ancient times, palpation was employed to detect abdominal stiffness as a sign of appendicitis,^432^ and it remains common today, particularly in traditional Chinese medicine for breast cancer screening.^433^ Benefiting from achievements in biomechanics and mechanobiology, the underlying mechanism of mechanical forces in disease occurrence and progression is unveiled. These advances have led to the development of mechanodiagnosis, a field that relies on detecting mechanomarkers for diagnosis (Figure 9). Mechanomarkers, classified by biomechanical features and mechanobiological markers, reflect changes between healthy and pathological conditions at tissue, cellular, and molecular scales.

#### Biomechanical features at tissue and cellular scales

Biomechanical features are the intrinsic physical and mechanical properties of tissues and cells that differ between healthy and diseased states. These features can be detected using various techniques at both tissue and cellular scales to aid in diagnosis.

At the tissue scale, common biomechanical features include viscoelasticity, stiffness, and IFP. Viscoelasticity often undergoes notable changes in diseases such as fibrosis, inflammation, and cancers.^434^^,^^435^ For instance, glioblastoma’s reduced viscoelasticity compared to normal brain tissue aids in distinguishing malignant from benign tissues.^436^ Tissue stiffness, crucial for cellular function, varies by tissue type. For example, normal liver stiffness is under 2.5 kPa, while stiffness reaches 4.11 kPa in fibrosis and 4.71 kPa in cirrhosis.^437^ IFP, representing the hydrostatic pressure in tissues, aids in distinguishing malignant from benign tumors and serves as a prognostic marker in some cancers.^438^ Additionally, unique physiological features, such as the geometry of brain tissue or blood flow shear stress, provide insight into tissue health. For instance, brain tissue geometry correlates with neurodevelopmental changes,^439^ while elevated shear stress can lead to endothelial dysfunction, raising atherosclerosis risk.^440^ These tissue-scale biomechanical features are associated with disease progression, making them valuable mechanodiagnostic markers.

To detect tissue-scale biomechanical features, elastography techniques such as UE and magnetic resonance elastography (MRE) are widely used. UE employs ultrasound waves to measure tissue displacement under mechanical force, providing stiffness information based on wave speed and displacement.^441^^,^^442^ MRE generates shear waves within the tissue through external mechanical excitation, and MRI is then used to capture and analyze these waves to assess tissue elasticity and viscoelasticity.^443^ Recently, wearable bio-adhesive ultrasound shear wave elastography (SWE) has enabled real-time detection of liver stiffness.^444^ This wearable device continuously monitors the liver’s elastic modulus by generating an acoustic radiation force pulse to induce shear waves. Its hydrogel-elastomer hybrid coupler has high moisture content, strong interface adhesion, and low acoustic attenuation, allowing for continuous monitoring up to 48 h.

Clinical applications of tissue-based mechanodiagnosis have developed significantly. Changes in tissue stiffness are increasingly used to diagnose and monitor disease progression. For instance, to improve imaging specificity in diagnosing sinus obstruction syndrome (SOS) post-hematopoietic stem cell transplantation, researchers applied transient elastography to measure liver stiffness as a predictive tool.^445^ Results indicate that liver stiffness averaged 5.9 kPa in individuals without SOS, while those with SOS show stiffness of 10.7 kPa, demonstrating a strong positive correlation between liver stiffness and SOS. The obtained detection sensitivity of 80% and specificity of 86% demonstrate the feasibility of using liver stiffness for SOS diagnosis. Similarly, liver stiffness can be used to predict and distinguish between fibrosis and cirrhosis, with stiffness values of 9 kPa for fibrosis and 13 kPa for cirrhosis.^446^ In breast cancer, viscoelasticity is notably higher than in normal tissues or benign lesions.^447^ Studies using SWE for breast lumps demonstrate that adding an SWE stiffness assessment increased diagnostic specificity from 54.8% to 66.1%,^448^ enhancing accuracy without reducing sensitivity. These applications highlight the importance of mechanodiagnosis in enhancing diagnostic efficiency through a non-invasive manner.

At the cellular scale, common biomechanical features include cell viscoelasticity, stiffness, contraction, and traction forces, as well as cellular communication with the ECM. Viscoelasticity reflects a cell’s elastic and viscous responses to mechanical forces, allowing adaptation to microenvironmental changes. These adaptations influence morphology, movement, proliferation, and differentiation, making viscoelasticity a dynamic marker for physiological and pathological conditions.^449^ Abnormal cell stiffness, often resulting from cytoskeletal alterations and cytokine interactions, serves as a label-free marker for cancer detection,^450^ with stiffness levels reflecting variations in cellular health. Contraction force, generated by actin-myosin interactions and exerted on the ECM through adhesion structures, helps to maintain stable tissue forces. Deviations in these traction forces are associated with pathological states such as cancer, fibrosis, and atherosclerosis, with abnormal traction forces specifically serving as biomarkers of metastatic potential and malignancy.^451^ These biomechanical features provide clues for understanding the pathogenesis and tracking the progression of diseases at the cellular scale. Additionally, microfluidic chips designed for detecting RBC deformability have been validated with clinical samples in diabetes and aging. For example, RBC deformability has been shown to differentiate T2DM with 90% sensitivity, as T2DM RBCs exhibit approximately 27% larger size and 29% lower stretch factor compared to healthy controls.^452^ Recently, hypo-osmotic treatment of RBCs has revealed significant differences between young and elderly individuals, with an area under the curve (AUC) of 0.84, 78.3% sensitivity, and 73.9% specificity, underscoring its potential for aging monitoring.^453^

Techniques for detecting cellular-scale biomechanical features include AFM, optical trapping, and traction force microscopy (TFM). AFM measures cellular biomechanical features, including stiffness, elasticity, and adhesion forces, by monitoring the deflection of a cantilever contacted with cell surfaces. Optical trapping uses highly focused laser beams to manipulate cells, enabling measurements of cell stiffness, viscoelasticity, and adhesion forces by analyzing applied forces and resulting cell deformation. TFM measures cell traction force by tracking the displacement of embedded fluorescent microspheres caused by cell contractions. These techniques show excellent performance in measuring cellular biomechanical features but are limited by expensive equipment and complex operations, making them unsuitable for clinical applications. Recently, microfluidic chips have emerged as a new approach for evaluating cellular biomechanical properties.^454^^,^^455^ This strategy primarily applies controlled fluid forces to cells within microchannels, allowing observation of cell deformation or movement in response to these forces to assess properties such as stiffness, elasticity, and viscoelasticity. By leveraging the distinct biomechanical differences between diseased and normal cells, microfluidic chips can be designed with specialized microstructures to separate different cell types. For instance, a diagonal ridge-shaped microfluidic channel, based on sheath flow technology and flow cytometry, has been designed to detect differences in stiffness and deformability of leukemia cells treated with the anticancer drug daunorubicin.^456^ This setup has successfully sorted drug-sensitive leukemia cells from drug-resistant ones, enabling the isolation of resistant cells post-chemotherapy. Additionally, the technology has also been used to sort Jurkat cells and white blood cells (WBCs), revealing that Jurkat cells are less stiff than WBCs.^457^ This allows high-accuracy enrichment of leukemia cells from a normal WBC population, further validating the feasibility of using microfluidic chips to detect cell stiffness.

Although cellular-scale mechanodiagnosis is not yet widely used in clinical practice, rapid advancements indicate its potential. For example, cellular biomechanical features can serve as “checkpoints” that influence immune responses.^458^ By linking cellular biomechanical changes with immune cell activation and evasion, these checkpoints can impact the progression of diseases such as tumors and fibrosis by modifying immune cell responsiveness. In liver fibrosis, increased ECM deposition and stiffness restrict macrophage and T cell migration and activation within cirrhotic tissue, thereby weakening immune clearance.

#### Mechanobiological markers at the molecular scale

Mechanobiological markers, including integrins and Piezo ion channels, reveal disease states by responding to mechanical forces within cells and the ECM. For example, alterations of integrins in tumors or atherosclerotic plaques offer insight into disease progression, making them potential indicators for diagnostic purposes.^459^ Piezo1, important in bone and cardiovascular health, plays a role in cellular behaviors under conditions such as osteoporosis and vascular diseases.^460^ The Ras association domain family 1A (RASSF1A) gene functions as a typical mechanobiological biomarker, with evidence showing that tumor tissue stiffness in the RASSF1A-deficient group (H1299 control cells) reached 16 kPa, significantly higher than that in the RASSF1A-expressing group.^461^ RASSF1A gene methylation has now become an important clinical biomarker for cancer diagnosis, detectable in body fluids such as blood and alveolar lavage fluid, making it a valuable non-invasive marker for early cancer detection. Detecting these markers often involves molecular imaging tools such as fluorescence resonance energy transfer (FRET) sensors^462^ and DNA tension probes,^463^ which provide high accuracy and enable extended observation of force transmission in cell membrane proteins. However, limitations in the continuous tracking of dynamic changes with FRET and other probes reduce their broad clinical applicability. As point-of-care (POC) techniques evolve, portable, low-cost, integrated detection techniques for mechanobiological markers are expected to improve the accuracy and efficiency of mechanodiagnosis, enhancing disease diagnosis and treatment. Molecular-scale mechanodiagnosis has the potential to facilitate the early disease diagnosis and the monitoring of therapeutic efficacy. For instance, fibronectin-targeted fluorescent aptamer DNA probes enable precise identification of fibrotic tissue in the early stages of liver fibrosis by specifically binding to fibronectin.^464^ ECM components, such as fibronectin and collagen type I, have been shown to be linked to treatment responses in patients with breast and gastric cancer.^465^ Although molecular-scale mechanodiagnosis is still in its early stage, with the revolutionary application of AI in mechanomedicine,^466^ the discovery of disease-specific mechanobiological markers and the development of highly efficient detection techniques would be accelerated.

#### Future remarks

Despite significant progress in mechanodiagnosis, several challenges persist before it can be fully integrated into routine clinical practice. Current detection methods for tissue- and cellular-scale biomechanical features often require expensive, complex instrumentation and highly specialized expertise. This limitation hinders widespread adoption, especially in resource-limited settings. Moreover, the identification and standardization of mechanomarkers remain a key bottleneck, where reliable reference values and comprehensive databases that differentiate healthy from pathological states are still lacking.

Emerging technologies and interdisciplinary approaches are poised to address these challenges. Advanced metamaterials, designed with engineered architectures that exhibit unique mechanical responses, can enable more sensitive, precise, and scalable biomechanical measurements. These novel materials, integrated with microfluidic chips or wearable devices, could facilitate continuous and non-invasive monitoring of biomechanical changes, especially at the POC.

AI holds great promise for enhancing mechanodiagnosis. By integrating large, multimodal datasets (e.g., spanning biomechanical signals, imaging, and clinical parameters), AI can identify subtle mechanomarker patterns and improve diagnostic accuracy. Predictive models can correlate specific biomechanical signatures with disease progression, enabling earlier and more personalized interventions. For example, several machine learning models have been utilized to provide patient-specific predictions of metastatic risk based on the innovative mechanobiology assay, facilitating accurate and early prognosis and classification.^467^ Moreover, AI-driven algorithms can accelerate the validation of novel mechanomarkers and guide the development of portable, low-cost POC devices, making mechanodiagnosis more accessible.

In the coming years, sustained collaboration among engineers, clinicians, materials scientists, and data scientists will be essential. By combining innovative metamaterial-based platforms, user-friendly POC testing technologies, and AI-driven analytic tools, the field can advance toward robust, affordable, and clinically relevant mechanodiagnostic solutions that improve patient outcomes worldwide.

### Cancer, vaccine design, and autoimmune disease

Mechanomedicine is an exciting frontier that takes advantage of the promising findings in biomechanics and mechanobiology to develop innovative therapies by targeting mechanical cues or mechanotransduction signaling, including the mechanical properties of cells and their microenvironments, as well as force-induced pathways.^340^ Mechanomedicine has opened new therapeutic avenues, particularly in cancer immunotherapy,^390^^,^^468^^,^^469^^,^^470^ vaccine design, and autoimmune disease treatment.^471^

#### Anticancer therapy and therapeutic implications of mechanoimmunology

Strategies that reduce tumor tissue stiffness have shown potential in impeding tumor progression.^472^ For example, decreasing tumor stiffness using lipid nanoparticles to deliver small interfering RNAs (siRNAs) targeting FAK and the CRISPR system can enhance drug delivery.^473^ Tetrathiomolybdate, a drug that inhibits lysyl oxidase activity and thus decreases tumor tissue stiffness, has shown promising results (median follow-up of 6.3 years) in treating patients with breast cancer in phase 2 clinical trials.^474^ A recent study, utilizing the machine learning algorithms, revealed tumor stiffness and hypoperfusion as biomarkers predictive of cancer treatment efficacy. Additionally, insights from mechanogenomic and mechanoepigenetic studies provide a foundation for engineering mechanically informed therapeutic strategies.^371^^,^^475^^,^^476^ Targeting mechanotransduction pathways, such as the integrin-FAK axis and YAP/TAZ signaling, can impede tumor growth and metastasis.^475^^,^^477^ For instance, in patients with Kras-mutant non-small cell lung cancer, FAK inhibitor defactinib (VS-6063) has demonstrated modest clinical effectiveness (median progression-free survival of 45 days) in phase 2 clinical trials.^478^ More recently, phase 1/2 trials of the Hippo-YAP-TEAD signaling inhibitor VT-3989 have exhibited a favorable safety profile (grade 1–2 toxicities) and desirable overall response rate (26%) in mesothelioma.^479^ Mechanomedicine also exploits the mechanical properties of tumor cells as new targets for therapy. Drugs such as 4-hydroxyacetophenone (4-HAP) increase cortical tension in tumor cells, which further reduces their invasiveness.^480^ Targeting cell softness can achieve the specific elimination of breast cancer stem cells.^481^ The inhibition of intercellular force propagation overcomes the resistance of tumor cells to chemotherapy.^482^

The interactions between cancer cells and the immune system in the context of mechanical cues have emerged as a critical area of research for cancer immunotherapies recently.^483^^,^^484^ The mechanics of TME plays critical roles in regulating anticancer immune responses. For example, although immune cells can be activated to greater extents when seeded on stiff matrix, increased ECM stiffness in tumor tissues leads to reduced infiltration of cytotoxic T cells and enhanced immune suppression via the induction of T cell exhaustion,^485^ thereby promoting tumor progression.^486^^,^^487^ On the other hand, recent studies have delved into the intricate interplay between cancer cell mechanics and immune responses, emphasizing how the physical properties of cancer cells influence immune surveillance, immune cell interactions, and therapeutic outcomes in cancer treatments. Upon formation of the IS, natural killer (NK) cells and cytotoxic T lymphocytes (CTLs) generate forces directed toward target cells, leading to lymphocyte activation and the establishment of a mature IS. Moreover, forces exerted at the IS directly impact lymphocyte-mediated cytotoxicity by increasing membrane tension and enhancing the lysis of target cells.^488^^,^^489^ It is essential to acknowledge that the biophysical properties of cancer cells play a significant role in regulating immunogenicity-mediated killing. Previous studies indicate that the softness of cancer cells, irrespective of other biochemical features, acts as a “mechanical immune checkpoint.” Soft cancer cells are more resistant to lymphocyte-mediated cytotoxicity.^490^^,^^491^^,^^492^ The integration of cancer cell mechanics into the realm of anticancer immune responses presents new opportunities for improving cancer treatment outcomes. Stiffening cancer cells can effectively enhance T cell- and NK cell-mediated cytotoxicity.^493^ These insights highlight two potential strategies for enhancing T cell cytotoxicity: engineering T cells to improve their adaptability to the mechanical properties of the TME and regulating the mechanical attributes of the TME to create favorable conditions for the normal activation of T cells.

By modulating mechanical forces and force-regulated immune receptor bond characteristics, mechanoimmunology offers a potential means to control immune activation, improving the specificity and efficacy of therapeutic interventions. In cancer immunotherapy, TCR catch-bond engineering has shown promising results. In contrast to previous attempts to engineer high-affinity TCRs to tumor antigens that led to fatal consequences in clinical trials, it is feasible to engineer TCRs to form catch bonds with tumor antigens but maintain relatively low affinity, resulting in improved anti-tumor potency and, at the same time, diminished on-target, off-tumor cross-reactivity to host tissues.^390^ A similar strategy exploiting mechanoimmunology may also work in chimeric antigen receptor (CAR) T cell therapy.^494^^,^^495^^,^^496^ It has been shown that the clustering of CAR molecules and IS formation are not as robust as those of TCRs.^497^^,^^498^ The difference in mechanical response between CAR and TCR may contribute to the altered IS structure in CAR T cells. By engineering TCRs or CARs to be more sensitive to mechanical forces or to exhibit catch-bond behavior, it may be possible to enhance T cell responses to tumor cells.^390^ Mechanical tuning of CARs can potentially increase binding stability under force with tumor antigens, allowing T cells to maintain long and strong interactions with cancer cells, leading to improved therapeutic efficacy and safety profiles.

The application of mechanoimmunology in vaccine design holds promise as well. Vaccines could be formulated to engage immune cells with optimal force profiles, enhancing the efficacy of antigen presentation and T cell priming. Particles or scaffolds that mimic the mechanical properties of APCs could improve T cell activation by stabilizing interactions at the IS, thus increasing the likelihood of a robust immune response. Furthermore, force-modulating adjuvants may be developed to enhance BCR or TCR engagement with specific antigens. Such approaches could potentiate immune responses against pathogens that normally evade detection by presenting low-affinity antigens. By mechanically amplifying these interactions, vaccines might elicit stronger and longer-lasting immunity, especially in cases where high-affinity immune responses are challenging to achieve.

In autoimmune diseases, restoring tolerance is a key therapeutic goal. By modulating mechanical forces, mechanoimmunology could help to recalibrate immune responses and reinstate tolerance to self-antigens. For example, therapies could be developed to attenuate the forces at the TCR-pMHC interface in a self-antigen-specific manner, thereby lowering TCR signaling below the activation threshold for self-reactive T cells. This approach could help prevent inappropriate activation of autoreactive T cells without compromising the immune system’s ability to respond to foreign antigens. Targeted mechanomodulation of the IS might also prevent or mitigate B cell-driven autoimmunity. By altering mechanical forces at the BCR-antigen interface, therapies could downregulate B cell activation in response to low-affinity self-antigens, effectively reducing autoimmune reactions. Therapeutic interventions that limit actin-driven pulling forces in B cells could also decrease antigen uptake and processing, which is crucial in cases where B cells present self-antigens to T cells inappropriately.

#### Further remarks

Despite the important progress in therapeutic attempts for cancer, vaccine design, and autoimmune disease informed by advances in biomechanics and mechanobiology, numerous challenges remain. These include the heterogeneity of cancer cell mechanical properties, the adaptive nature of the TME, and the complex, evolving interactions between cancer cells and immune cells. Future efforts should focus on unraveling the mechanisms underlying how cancer cell mechanics dynamically influences immune responses and developing targeted therapeutic strategies that exploit these mechanical cues to enhance anticancer immune responses. Meanwhile, the therapeutic applications of mechanoimmunology are still in the early stages. Characterizing the mechanical properties of TCRs and BCRs in different immune contexts and understanding how force thresholds vary between self- and foreign antigens are essential for designing effective therapies. Moreover, developing precise tools for modulating mechanical forces within tissues and the IS will be crucial for the success of mechanosensitive immune therapies. By harnessing and modulating the mechanical forces that govern immune receptor activation, it may be possible to unlock new possibilities for treating cancer, enhancing vaccine efficacy, and restoring immune tolerance in autoimmune diseases. To address these challenges, future efforts should leverage emerging mechanical probing technologies, multiscale biophysical modeling, and AI-driven predictive frameworks. These approaches will enable more precise characterization of mechanical cues, deeper mechanistic insights into immune regulation, and the rational design of next-generation mechanotherapeutic interventions.

In addition to these cases discussed above, some innovative applications of mechanobiology in areas such as anti-aging, stem cell therapeutics, and the management of platelet-related diseases have emerged. For example, interventions targeting aging-dependent ECM remodeling provide promising strategies for counteracting cellular deterioration and tissue degeneration.^499^^,^^500^ Moreover, recent advances highlight mechanobiological conditioning of MSCs, an appealing therapeutic cell type for many diseases, can reduce their senescence while enhancing their proliferation, multipotency, and regenerative capacity, offering potential mechanobiology-based therapeutic strategies.^501^^,^^502^^,^^503^ In addition, growing evidence from platelet mechanobiology has demonstrated that mechanical cues act as important regulators of platelet reactivity and identified mechanosensitive signaling pathways that offer promising targets for treating platelet-related disease, such as arterial thrombosis.^504^ Further investigation in these areas will help translate mechanistic insights into impactful clinical applications.

### Tissue damage repair and functional reconstruction

**Figure 10:**
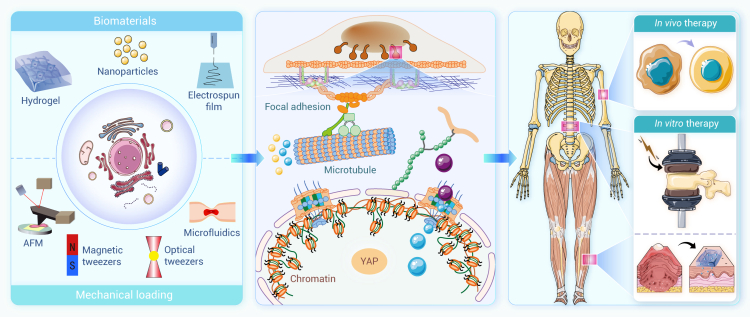
A summary of the approach to and therapeutic mechanism of tissue damage repair and functional reconstruction based on mechanobiology

Tissue damage or tissue dysfunction due to aging and disease is one of the most important factors affecting people’s daily lives. Currently, conventional surgical treatments cannot completely repair the damaged tissue and restore its function and may lead to fibrosis.^505^^,^^506^ Therefore, there is an urgent need to develop new therapeutic strategies. Recently, mechanomodulation technologies have been developed rapidly and gradually applied to the treatment of clinical diseases.^507^^,^^508^ On this basis, an emerging research direction—mechanotherapy—has emerged. It begins with the development of mechanomodulation approaches, followed by the elucidation of how mechanical cues regulate cell behavior and fate through underlying mechanobiological mechanisms, and ultimately advances toward the construction of functional microtissues *in vitro* or the direct implementation of therapeutic interventions *in vivo* (Figure 10). In this section, typical soft tissues (e.g., skeletal muscles) and soft-hard interfacial tissues (e.g., PDL-AB enthesis and bone-cartilage enthesis) have been chosen to systematically elaborate the approach, application, and therapeutic mechanism of mechanotherapy.

#### Repair of skeletal muscles

Skeletal muscles, as important soft tissues that support the body’s vital activities, play an important role in body movement, maintenance of body temperature, and postural support. Skeletal muscle functional impairment triggered by aging, chronic degenerative diseases (e.g., muscular dystrophy),^509^ metabolic defects (e.g., Pompe disease),^510^ and volumetric muscle loss^511^ due to trauma or surgical resection is a serious threat to human health and needs to be addressed urgently. In traditional clinical practice, muscle dysfunction is primarily managed through ultrasound therapy, functional electrical stimulation, and isometric-isotonic exercises, which aim to enhance mechanical properties and restore function.^512^^,^^513^ Recently, accumulating evidence has indicated that improving the biophysical microenvironment of myocytes has a regulatory effect on the physiological structure and function of skeletal muscle.^514^^,^^515^^,^^516^ Therefore, many mechanotherapeutic strategies have been developed for *in vitro* engineering of functional muscle microtissues through mimicking the mechanical microenvironment of skeletal muscle and regulating cell behavior of myoblasts, showing great promise in providing potential clinical solutions for the treatment of skeletal muscle-related diseases and defect repair. It has been discovered that the skeletal muscle of healthy mice has nonlinear viscoelastic properties and exhibits strain-enhanced stress relaxation behavior. However, this feature is virtually absent in the muscle tissue of Duchenne muscular dystrophy mice.^517^ Subsequently, a 3D nonlinear viscoelastic cell microenvironment was constructed using collagen hydrogels, recapitulating the strain-enhanced stress relaxation phenomenon observed in native skeletal muscle. Such nonlinear viscoelasticity in healthy muscle tissues enhances myogenic differentiation of myoblasts through actin-polymerization-triggered nuclear localization of MRTF and nuclear mechanotransduction.^517^ This finding would provide a mechanobiology consideration for the development of a new mechanotherapy that targets the mechanical properties of skeletal muscles for the clinical treatment of muscle injuries. In addition, skeletal muscles are chronically stimulated by mechanical stretching from tendon pulling. To mimic such a mechanical cue, Bian et al. fabricated cell-laden hydrogel micropillar arrays using photomasking and guided cell alignment via passive tension arising from cell contraction, leading to the formation of muscle fiber bundles with contraction characteristics resembling those of native muscles.^518^ As a mechanically sensitive tissue, skeletal muscle generates mechanical stretch due to muscle movement that is transmitted to myocytes through ECM structures. In order to simultaneously simulate the above two microenvironmental factors, including mechanical stretch and muscle fiber orientation, Shi et al. developed a 3D, magnetically actuated, aligned collagen fiber hydrogel platform that could recapitulate aligned ECM architecture and mechanical stretch.^519^ This dual-mechanical cue synergistically promotes myogenesis through modulating YAP subcellular localization and the structure of cellular microtubule networks, providing new therapeutics for *in vivo* muscle injury from the perspective of mechanobiology.

#### Soft-hard interfacial tissue regeneration

The soft-hard interfacial tissue is capable of effectively transferring mechanical stress, relieving stress concentrations at the tissue interface, which plays an important role in many motor functions of the human body.^520^ For example, the PDL-AB enthesis serves to anchor the roots of the teeth and relieve the pressure generated during chewing. Biophysical and biochemical microenvironmental cues at the soft-hard interfacial tissue are characterized by typical gradient distributions.^521^^,^^522^ The matrix stiffness gradient from soft to hard tissue is one of the important biophysical cues. A previous study indicated that the matrix stiffness gradient at the PDL-AB enthesis is closely related to its physiologic or pathologic state and plays an important role in modulating the immune phenotype of MSCs at the interface.^523^ Compared with the PDL-AB enthesis in healthy rats, the stiffness gradient significantly decreases at the PDL-AB enthesis in periodontitis. By designing and fabricating two-layered polyacrylamide gel hybrids for recapitulating the matrix stiffness gradients *in vivo*, it was revealed that the low stiffness gradient at the PDL-AB enthesis in periodontitis induces the anti-inflammatory phenotype (i.e., MSC2) of MSCs by reducing cell polarization. This process involves a reduction in polarization of integrin β1 clustering and myosin IIB, leading to H4K16ac localization-dependent chromatin remodeling that contributes to the expression of anti-inflammatory genes (e.g., Toll-like receptor 3 [TLR3]).^523^ This study not only provides mechanically biomimetic strategies for the rational design of soft-hard tissue interfaces in interfacial tissue engineering but also offers novel mechanotherapeutic strategies for periodontal-related diseases. Beyond traditional scaffold design, novel bio-hybrid approaches are emerging. Advanced materials such as electrospun scaffolds and piezoelectric systems enhance stress distribution and promote the functional restoration of both hard and soft tissues within the cementum-PDL-AB complex.^524^^,^^525^^,^^526^ For instance, clinical studies are exploring mechano-responsive implants, such as piezoelectric membranes or scaffolds that generate electrical potentials in response to mechanical loading (e.g., mastication), to stimulate osteogenesis and promote PDL regeneration.^526^ Additionally, force-sensing biosensors integrated into temporary prosthetic devices or orthodontic appliances are being developed to enable real-time monitoring of occlusal forces, providing valuable data for optimizing load distribution in periodontal therapy and preventing overload-induced tissue damage.^527^^,^^528^

Another typical soft-hard interfacial tissue is the bone-cartilage enthesis, which not only achieves fatigue-resistant adhesion between cartilage and bone with completely different compositions, structures, and mechanical properties but also allows for effective mechanotransduction without stress concentration during millions of cyclic loadings per year.^529^^,^^530^ Ouyang’s team investigated and delineated the bone-cartilage enthesis in the human knee joint from the ultrastructural, compositional, and histomechanical perspectives and elucidated the potential mechanisms underlying its high-strength mechanotransduction and anti-fatigue adhesion.^531^ Mechanomodulation technologies developed based on the above mechanisms can utilize a limited number of components, precise structural assemblies, highly ordered spatial gradients, and integrated designs to endow engineered tissues with optimal mechanical performance within a minimal spatial footprint. The clinical application of this mechanotherapeutic strategy would greatly reduce the incidence of diseases such as osteoarthritis caused by bone-cartilage interface damage and demonstrates great potential in the regeneration of interfacial tissues and the restoration of mechanotransduction properties.

#### Future remarks

Despite notable progress in mechanotherapy, there remain several challenges that limit its widespread clinical application. Achieving precise control over the complex mechanical cues (e.g., nonlinear viscoelasticity, spatial stiffness gradients, and dynamic mechanical loading) remains challenging. Conventional scaffolds and devices often lack the ability to fine-tune multiple mechanical parameters simultaneously, limiting their ability to fully recapitulate native tissue environments. Furthermore, current approaches sometimes overlook the interplay between mechanical cues and other factors, including biochemical gradients, immune responses, and patient-specific variability, all of which can significantly influence therapeutic outcomes.

Emerging technologies offer exciting prospects for overcoming these challenges. Metamaterials have the potential to deliver highly customizable mechanical cues that closely mimic complex, tissue-specific conditions. By integrating metamaterials into 3D scaffolds, researchers can create microenvironments with tunable stiffness, anisotropy, and viscoelastic profiles, enabling more refined mechanomodulation strategies. Additionally, the advent of soft micro-/nano-robotics provides dynamic mechanical inputs that can adjust to real-time feedback from regenerating tissues. These adaptable systems, powered by advanced sensors and actuators, can apply controlled stretching, compression, or bending forces, promoting optimal cellular responses and facilitating functional tissue regeneration.

In parallel, advances in automation, AI, and computational modeling are improving our ability to rationally design, predict, and optimize mechanotherapeutic interventions. Such tools can integrate patient-specific data, enabling precision mechanotherapy tailored to individual needs. With advances in these approaches, the convergence of metamaterials, soft robotics, and AI-based rational design promises to accelerate the translation of mechanotherapy from bench to bedside. Ultimately, these integrative strategies will pave the way for more effective, reliable, and personalized regenerative therapies that restore tissue function and improve patient quality of life.

### Deformity correction and functional compensation

**Figure 11:**
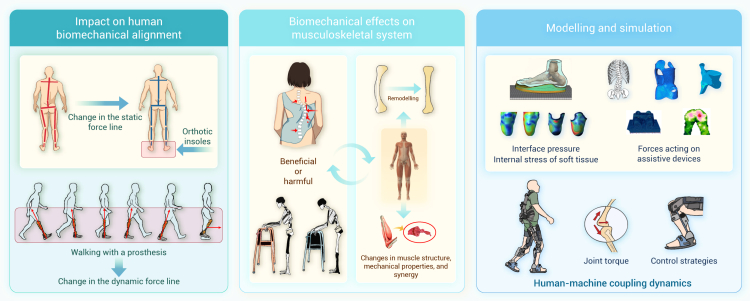
Typical biomechanical issues and simulation analysis of assistive technology in deformity correction and functional compensation

Rehabilitation engineering is one of the key application areas of biomechanics and mechanobiology. It refers to the systematic application of mechanics and engineering principles, technologies, and methods to overcome human functional impairments or disabilities. The ultimate goal is to restore or compensate for lost functions as fully as possible, alleviating limitations in mobility or participation and facilitating maximum independence in daily life and reintegration into society.^532^ Rehabilitation assistive technology is a vital practical embodiment of biomechanics and mechanobiology in rehabilitation engineering. It primarily involves the use of assistive devices to protect, support, train, measure, and substitute for body function (structure) and to reduce activity limitations and minimize participation restrictions.^533^ This section will focus on rehabilitation assistive technology, delving into its applications, challenges, and emerging trends in biomechanics and mechanobiology research aimed at correcting musculoskeletal deformities and compensating for motor function impairments (Figure 11).

#### Correction and functional compensation of musculoskeletal deformities

Musculoskeletal deformities in the upper limbs, lower limbs, and spine caused by neurological, muscular, and skeletal diseases require assistive devices such as orthoses to provide deformity correction, joint stabilization, and pressure relief.^534^^,^^535^^,^^536^^,^^537^ The biomechanical optimization design, along with the evaluation of its short-term and long-term biomechanical effects and rapid manufacturing technologies, is currently the key research focus in this field. Lower limb orthoses provide weight-bearing support and assist with walking.^538^ Key design considerations include the static and dynamic alignment of the hip-knee-ankle joints, its biomechanical effects on gait, and the analysis of the contact surface force between soft tissue and orthoses.^539^ Upper limb orthoses are designed to support, stabilize, and correct the functionality of the upper limb, with specifications tailored to meet the user’s functional activity needs and mechanical support requirements.^540^ For spinal orthoses, prominent research topics include the mechanisms of load transmission across different spinal segments under various activity states and the resulting biomechanical effects. Current research typically investigates these effects through gait analysis, physical measurements, medical imaging, and assessments of the mechanical properties of soft tissues and their interface loading.^541^ Computational biomechanics methods are essential for analyzing the mechanical properties of orthotic devices and the internal stress distribution within soft tissues.^542^ In addition to orthoses, distraction osteogenesis has also been widely applied in the management of musculoskeletal deformities. This clinically applied technique utilizes controlled mechanical forces generated by fixation devices to stimulate new bone formation and guided regeneration. Distraction osteogenesis is underpinned by the proliferation of osteoblasts and bone marrow-derived MSCs (BMSCs), orchestrated through signaling pathways including Wnt/β-catenin and bone morphogenetic protein 4 (BMP4).^543^ Periodic distraction forces enhance osteogenesis-related gene expression (e.g., RUNX2 and alkaline phosphatase [ALP]) and regulate actin cytoskeleton remodeling through cofilin.^544^ Meanwhile, integrin αVβ3 is also involved in enhancing bone formation within distraction regions by promoting vascularization and improving nutrient supply.^545^ Despite its clinical success, challenges, such as poor callus formation, infection, and long treatment duration, remain. Currently, traditional passive-correction orthoses remain the most widely used devices in clinical practice. However, intensive research and development efforts are driving their transition toward intelligent, active-correction systems. Smart orthoses equipped with integrated sensing, monitoring, and stimulation functions represent a major frontier of innovation, with numerous prototypes already emerging.^546^ Translating these research advancements into routine clinical care still requires larger-scale clinical applications to further refine and validate their performance. With the rapid development of additive manufacturing technologies and advanced materials, orthoses are expected to achieve fast and personalized customization, balancing comfort and functionality while offering new possibilities for biomechanical optimization.^547^

Temporomandibular disorders (TMDs) are a class of musculoskeletal degenerative conditions that lead to functional and morphological deformities of the TMJ.^548^^,^^549^ Occlusal splints, commonly used in clinical practice, alter TMJ neuromuscular reflexes and reduce intra-articular pressure, serving as an effective treatment for TMDs.^328^ Integrating biomechanical principles, occlusal splints are designed to adjust joint load distribution according to the patient’s mechanical characteristics, thereby alleviating excessive friction and pressure on the TMJ.^550^ Meanwhile, physical therapy, such as functional training, low-frequency electrical stimulation, and laser therapy, can improve the mechanical adaptability of the TMJ and promote rehabilitation by modulating joint movement patterns.^551^^,^^552^ In addition, technologies such as computer-aided design (CAD) and 3D printing enable customized solutions, allowing for more precise and efficient restoration of biomechanical function.^553^ With advances in technology, AI-enabled wearable devices for real-time monitoring are emerging as promising tools in musculoskeletal rehabilitation. For instance, recent clinical trials have explored wearable electromyography sensors integrated with AI algorithms to deliver real-time biofeedback on masticatory muscle activity, enabling personalized rehabilitation protocols for patients with TMD and improving treatment outcomes.^554^

#### Replacement and functional compensation of residual limbs

For individuals who have undergone amputations, prosthetic devices are essential for providing functional replacement and weight-bearing support,^555^ aiming to facilitate amputees’ daily activities and enhance their quality of life. Key research areas in prosthetic biomechanics include the assessment of residual limb mechanical properties, the analysis of stress distribution at the prosthetic socket interface, and the static and dynamic alignment of the prosthesis.^556^^,^^557^ As early as 1987, finite element simulation models were developed specifically for lower limb prostheses to examine the mechanical interactions between the residual limb and the prosthetic socket.^558^ Over the past few decades, researchers have consistently explored the complex simulations of the residual limb-padding-socket model,^559^ interface pressure and shear stress under both dynamic and static loading conditions,^560^ internal stress within residual soft tissues,^561^ and personalized rapid modeling and simulation methods. Effective prosthetic design requires prosthetic structures, actuators, and control systems to closely replicate the biomechanics of natural limb movement, with careful consideration of motion coordination and functional range of motion.^562^ The prosthetics field has undergone transformative development since the 20th century, with the global market dominated by several long-established manufacturers while being continually invigorated by a growing number of innovative companies. Upper limb prostheses are typically controlled by electromyographic signals from the residual limb, with an emphasis on achieving a wide range of motion and precise mechanical control of individual joints.^563^ In contrast, lower limb prostheses generally employ mechanical control or electric actuation, focusing on ensuring gait stability and enabling adaptive gait control strategies.^564^ These advanced lower limb devices are now widely applied in clinical practice, significantly improving mobility and quality of life for amputees. Improving the robustness of prostheses to variations in environmental conditions and human motion, as well as developing personalized adaptive control mechanisms,^565^^,^^566^ are currently prominent research hotspots with significant potential to enhance user experience.

#### Compensation and training for motor dysfunction

In rehabilitation medicine, assistive technologies such as mobility aids and rehabilitation robots are widely utilized to provide rehabilitation training and functional compensation for individuals with motor impairments. This process entails addressing a series of scientific questions related to musculoskeletal biomechanics and movement biomechanics.

Mobility aids, including devices such as walkers, crutches, and wheelchairs, are commonly utilized assistive technologies designed for individuals with motor functional impairments.^567^ These devices have become integral components of clinical rehabilitation and long-term care worldwide. Most research focuses on exploring the characteristics of mechanical loads, muscle coordination, and kinematic performance of users’ upper and lower limbs during the use of walkers and wheelchairs.^568^ Understanding these factors is crucial for optimizing the functionality and ergonomic design of assistive devices^569^ and avoiding damage caused by abnormal stress on muscles and bones.^570^ In addition, stress analysis at the human-wheelchair support interface is a critical research focus for elucidating the mechanisms of soft tissue injury,^571^ offering valuable guidance for optimizing device design to minimize adverse effects.^572^

Rehabilitation robots exemplify the application of advanced robotic technology in the field of rehabilitation assistive devices. This field originated in the 1980s and has undergone substantial development since the 1990s, marked by significant advancements in both technological innovation and methodological approaches.^573^ At present, various robotic systems for upper and lower limb rehabilitation are being increasingly adopted in clinical settings, providing standardized and intensive training for patients with neurological or musculoskeletal disorders. Biomechanical research associated with rehabilitation robot technology encompasses several key areas, including robot design,^574^^,^^575^ human-robot interaction,^576^^,^^577^ and biomechanical assessments of rehabilitation processes.^578^ Researchers have employed various modeling and simulation methods to investigate human-robot coupling dynamics and elucidate how forces and movements transfer between the mechanical devices and the human body.^579^ These studies help to guide the design of the robot’s structure, motors, and other components, ensuring that they meet the biomechanical needs of users. However, accurately modeling and measuring the interaction forces between humans and robotic systems remains one of the major challenges in this field.^580^ Addressing these challenges is critical to advancing rehabilitation robotics and ultimately enhancing therapeutic outcomes for individuals undergoing rehabilitation.

#### Future remarks

The widespread application of rehabilitation engineering in assistive technology underscores the inherent complexity of its biomechanical challenges. Over the past few decades, research in this field has primarily centered on two fundamental issues: (1) how the design of rehabilitation devices can be optimized to more effectively compensate for human functional impairments and (2) whether the long-term use of such devices entails any potential risks. Notably, the biomechanical properties of muscles, bones, and soft tissues vary significantly across different regions of the human body. Moreover, the kinematic and kinetic characteristics and the biomechanical responses of the human body during daily activities are highly variable. These complexities necessitate careful consideration of both the immediate and long-term biomechanical effects in the design of rehabilitation assistive devices. However, several limitations still persist. For instance, it remains unclear whether active orthoses offer superior long-term therapeutic benefits compared to their passive counterparts. In addition, the control strategies used in current prostheses and rehabilitation robots often show limited adaptability to complex, real-world environments. Advancing these rehabilitation engineering technologies will require deeper investigation into the underlying biomechanical mechanisms, alongside rigorous clinical validation of their efficacy. At the core of these design considerations lies the challenge of optimizing the mechanical interaction between the device and the human body. Most assistive devices interact with the human body through soft tissues and function by providing corrective forces and motion control via mechanical interfaces. The fundamental design problem is to ensure that these forces are sufficient to achieve therapeutic goals—such as alignment correction or movement assistance—while avoiding excessive stress or tissue damage. In response to these challenges, intelligent sensing and control have become key research priorities in the field. Advanced sensing technologies enable the continuous acquisition of users’ physiological signals and motion states, which form the basis for enhancing human-machine interaction. By integrating real-time data from embedded sensors and analyzing them through sophisticated control algorithms, assistive devices can assess functional impairments, infer movement intentions, and identify usage scenarios. This capability supports adaptive control strategies, allowing the device to dynamically adjust its mechanical outputs in response to the user’s needs. The advancement of such technologies is expected to render rehabilitation devices that are increasingly intelligent, personalized, and precise, thereby significantly enhancing therapeutic outcomes and improving users’ quality of life.

## Conclusion and future perspectives

In this review, we have provided a comprehensive overview of the history, development, and current research directions in biomechanics and mechanobiology, highlighting significant advancements in areas such as cardiovascular systems, bones and joints, ocular tissues, liver, lung, the craniomandibular system, cancer, and immunology. Meanwhile, we have also explored the pivotal applications of biomechanics and mechanobiology in disease diagnosis, treatment, and rehabilitation, demonstrating their critical roles in advancing medical science and improving clinical outcomes.

With today’s rapid evolution of the scientific landscape, technological innovation and interdisciplinary convergence are reshaping research paradigms. Scientific exploration is continually pushing the boundaries of the macroscopic scale, delving deeper into the microscopic realm, and pursuing knowledge under extreme conditions. The advent of the digital era and the integration of AI are accelerating breakthroughs in fundamental scientific problems and fostering the emergence of new disciplines. These developments present both opportunities and challenges for the field of biomechanics, necessitating adaptive and forward-thinking approaches to harness their transformative potential.

To advance this field, we propose several key directions for future research.

(1) Fostering interdisciplinary integration: biomechanics must continue to strengthen its connections with clinical practice and other disciplines such as engineering, chemistry, and physics. Moreover, much more effort should be made to fully leverage advancements in materials science and technology. Such integration will help to drive methodological breakthroughs and contribute to addressing complex health challenges.
(2) Leveraging AI: currently, AI is being widely applied across numerous fields, establishing new paradigms and offering innovative insight for scientific research. The integration of AI into biomechanics holds great promise for enhancing biomechanical analysis, improving diagnostic accuracy, and advancing personalized therapeutic strategies. For example, AI-driven biomechanical analysis enables more accurate insights into the mechanical behaviors of cells and tissues, contributing to a deeper understanding of disease progression. In diagnostics, the integration of AI with imaging techniques and biomechanical features significantly enhances the sensitivity and accuracy of early disease detection. In therapeutics, AI-optimized mechanical treatment strategies offer personalized therapeutic plans tailored to individual patients, significantly improving clinical outcomes. Meanwhile, by integrating large-scale mechanobiology datasets, including mechanotransduction pathways, cellular response profiles, and clinical outcome data, AI algorithms can help screen and predict mechanotherapeutic drug candidates. Fully leveraging AI is expected to revolutionize disease prevention, diagnosis, and treatment by providing a deeper understanding and more precise interventions.
(3) Advancing biomechanical modeling and simulation: biomechanical modeling and simulations, as indispensable tools, have been widely applied in, e.g., cardiovascular, musculoskeletal, and ocular systems. Given the multi-level structural complexity of biological systems, the evolution of biomechanical modeling from simple, static, 1D idealized mathematical models to complex, dynamic, 3D models based on realistic anatomical structures is essential and inevitable. Developing a cross-scale coupling model will help to elucidate the mechanical properties and responses across different structural levels and uncover the mechanisms linking these structural hierarchies. Such knowledge will contribute to a more comprehensive understanding of physiological states and pathological processes, as well as provide a robust scientific foundation for optimizing therapeutic strategies and innovative medical solutions, thereby supporting precision medicine and personalized treatment.
(4) Exploring biomechanics in extreme environments: with the continuous expansion of human activity into extreme environments, such as microgravity, hypergravity, and deep-sea conditions, investigating their physiological and pathological implications has become increasingly important and urgent. Understanding how organisms respond to these conditions and developing new concepts, technologies, and methodologies based on biomechanical principles will inform the development of effective protective measures and safeguard strategies.
(5) Expanding research on nuclear biomechanics and emerging subfields: the nucleus, serving as the central hub for the storage, replication, and transcription of genetic material within cells, plays a pivotal role in regulating life activities. Emerging areas such as mechanogenomics and mechanoepigenetics are shedding light on the molecular mechanisms by which mechanical forces influence nuclear structure, chromatin conformation, and gene expression. These insights are critical for understanding physiological functions and identifying novel therapeutic targets.
(6) Developing traditional biomechanics: despite significant advancements in biomechanics that have fueled the cultivation and development of emerging fields, many unresolved issues remain in traditional biomechanics, and further in-depth exploration is indispensable. Addressing challenges such as *in vivo* characterization of biofluid dynamics, constitutive relations of living material, and the properties of forces and their biological implications will provide fundamental support for further understanding life phenomena and uncovering disease mechanisms.

In summary, biomechanics has made significant contributions to understanding fundamental principles-of-life activities, uncovering the pathogenesis of diseases, and developing novel therapeutic strategies. As this field encounters new opportunities and challenges, it must continue to expand across spatial and temporal scales, foster interdisciplinary integration, and pursue methodological innovation. By doing so, biomechanics and mechanobiology will remain at the forefront of addressing complex biomedical challenges, ultimately benefiting human health and well-being.

## Funding and acknowledgments

This work was funded by the Strategic Priority Research Program of the Chinese Academy of Sciences (CAS) (grant no. XDB0620101), the 10.13039/501100001809National Natural Science Foundation of China (grant nos. 12232019, 12272388, 11902327, 12472316, 12032003, 12072235, 32271371, U23A6009, and 12272029), the Youth Innovation Promotion Association of the CAS, the Hong Kong Research Grant Council (PolyU 15227523 and C5016-23G), the Health and Medical Research Fund (HMRF18191421), the Nankai University Institute of Ophthalmology (NKYKD202205), and the 10.13039/501100012226Fundamental Research Funds for the Central Universities.

## Author contributions

Chunqiu Zhang, Min Zhang, J.L., Y.T., Mian Long, Y.Q., W.C., F.X., Y.F. and F.S. conceived and designed the research. H.R., Q.Y., K.H., D.K., Y.Q. wrote the cardiovascular system section. L.G., H.G., Y.S., Xianglong Lin, X.W., Q.C., Chunqiu Zhang wrote the bone and joints section. Xiaona Li, X.Q., J.S., R.D., J.J., A.E., W.C. wrote the eye section. N.L., D.L., Y.D., Mian Long wrote the liver section. B.C., L.D. wrote the lung section. S.Z., F.F., Y-L.L., Yanli Liu, Z-J.L., Min Zhang wrote the craniomandibular system section. K.T., Meiying Luo, Cunyu Zhang, G.H., Y.T. wrote the cancer section. Y.J., H.C., Baoyu Liu, J.L. wrote the mechanoimmunology section. Zedong Li, Y.M., S.W., R.Y., F.X. wrote the mechanomarkers development and tissue damage repair and functional reconstruction sections. W.R., F.P., B.X., Ming Zhang, Y.F. wrote the deformity correction and functional compensation section. Yan Li, Chuanrong Zhao, X.S., L.W., Bo Li, Yonggang Lü, Z.Z., G.W., Zhiyong Li, Yiyao Liu, W.T. participated in the discussion of the paper outline and provided valuable suggestions. L.L. was involved in the writing and revision of all the content of the article. All authors contributed to the manuscript and approved the final version.

## Declaration of interests

The authors declare no competing interests.

## Supplemental information

Document S1. Tables S1 and S2

Document S2. Article plus supplemental information

## References

[1] Dance A. (2021). Lifeforce. Nature.

[2] Wang Q., He S., Ji B. (2024). How do multiple active cellular forces co-regulate wound shape evolution?. J. Mech. Phys. Solid..

[3] Xu J., Wang Q., Li X. (2023). Cellular mechanisms of wound closure under cyclic stretching. Biophys. J..

[4] Xu J., Xu X., Li X. (2022). Cellular mechanics of wound formation in single cell layer under cyclic stretching. Biophys. J..

[5] Lian L., Xie M., Luo Z. (2024). Rapid volumetric bioprinting of decellularized extracellular matrix bioinks. Adv. Mater..

[6] Du R., Wu Y., Zou M. (2025). Intracellular pressure: Regulation, characterization and implication. Sci. China Phys. Mech. Astron..

[7] Jiang Z.L. (2023). Mechanobiology research in china. Mechan. Med.

[8] Fung Y.C. (1990).

[9] Peña O.A., Martin P. (2024). Cellular and molecular mechanisms of skin wound healing. Nat. Rev. Mol. Cell Biol..

[10] Ajalloueian F., Lemon G., Hilborn J. (2018). Bladder biomechanics and the use of scaffolds for regenerative medicine in the urinary bladder. Nat. Rev. Urol..

[11] Boldt J. (2001). Cardiovascular system. Curr. Opin. Crit. Care.

[12] Hu Q., Fang Z., Ge J. (2022). Nanotechnology for cardiovascular diseases. Innovation.

[13] Wang D., Maharjan S., Kuang X. (2022). Microfluidic bioprinting of tough hydrogel-based vascular conduits for functional blood vessels. Sci. Adv..

[14] Jia M., Miao W., Li Y. (2025). A polymerized probucol nanoformulation with neutrophil extracellular vesicle camouflage for cerebral ischemia-reperfusion injury therapy. Innovation.

[15] Ren H., Hu W., Jiang T. (2024). Mechanical stress induced mitochondrial dysfunction in cardiovascular diseases: Novel mechanisms and therapeutic targets. Biomed. Pharmacother..

[16] Zhang W., Sommer G., Niestrawska J.A. (2022). The effects of viscoelasticity on residual strain in aortic soft tissues. Acta Biomater..

[17] Zhang W., Liu Y., Kassab G.S. (2007). Viscoelasticity reduces the dynamic stresses and strains in the vessel wall: Implications for vessel fatigue. Am. J. Physiol. Heart Circ. Physiol..

[18] Garoffolo G., Pesce M. (2019). Mechanotransduction in the cardiovascular system: From developmental origins to homeostasis and pathology. Cells.

[19] Wang Y., Zhang K., Qin X. (2019). Biomimetic nanotherapies: Red blood cell based core-shell structured nanocomplexes for atherosclerosis management. Adv. Sci..

[20] Wang Y., Zhang K., Li T. (2021). Macrophage membrane functionalized biomimetic nanoparticles for targeted anti-atherosclerosis applications. Theranostics.

[21] Pesce M., Duda G.N., Forte G. (2023). Cardiac fibroblasts and mechanosensation in heart development, health and disease. Nat. Rev. Cardiol..

[22] Davis M.J., Earley S., Li Y.S. (2023). Vascular mechanotransduction. Physiol. Rev..

[23] Lim X.R., Harraz O.F. (2024). Mechanosensing by vascular endothelium. Annu. Rev. Physiol..

[24] Long Y., Niu Y., Liang K. (2022). Mechanical communication in fibrosis progression. Trends Cell Biol..

[25] Corral-Acero J., Margara F., Marciniak M. (2020). The 'digital twin' to enable the vision of precision cardiology. Eur. Heart J..

[26] Kamel Boulos M.N., Zhang P. (2021). Digital twins: From personalised medicine to precision public health. J. Personalized Med..

[27] Chen Y.C., Zheng G., Donner D.G. (2024). Cardiovascular magnetic resonance imaging for sequential assessment of cardiac fibrosis in mice: Technical advancements and reverse translation. Am. J. Physiol. Heart Circ. Physiol..

[28] Reiter G., Reiter C., Ovcina I. (2025). Four-dimensional flow MRI for a dynamic perspective on the heart and adjacent great vessels. Radiology.

[29] Benech J.C., Romanelli G. (2022). Atomic force microscopy indentation for nanomechanical characterization of live pathological cardiovascular/heart tissue and cells. Micron.

[30] Owen B., Bojdo N., Jivkov A. (2018). Structural modelling of the cardiovascular system. Biomech. Model. Mechanobiol..

[31] Guan D., Gao H., Cai L. (2022). A new active contraction model for the myocardium using a modified hill model. Comput. Biol. Med..

[32] Li D.S., Mendiola E.A., Avazmohammadi R. (2023). A multi-scale computational model for the passive mechanical behavior of right ventricular myocardium. J. Mech. Behav. Biomed. Mater..

[33] Kummer T., Rossi S., Vandenberghe S. (2022). Embedded computational heart model for external ventricular assist device investigations. Cardiovasc. Eng. Technol..

[34] Sigaeva T., Zhang Y. (2023). A novel constitutive model considering the role of elastic lamellae' structural heterogeneity in homogenizing transmural stress distribution in arteries. J. R. Soc. Interface.

[35] Grande Gutiérrez N., Sinno T., Diamond S.L. (2022). A 1D-3D hybrid model of patient-specific coronary hemodynamics. Cardiovasc. Eng. Technol..

[36] Nagueh S.F., Smiseth O.A., Appleton C.P. (2016). Recommendations for the evaluation of left ventricular diastolic function by echocardiography: An update from the American society of echocardiography and the European association of cardiovascular imaging. J. Am. Soc. Echocardiogr..

[37] Koo B.K., Erglis A., Doh J.H. (2011). Diagnosis of ischemia-causing coronary stenoses by noninvasive fractional flow reserve computed from coronary computed tomographic angiograms. Results from the prospective multicenter discover-flow (diagnosis of ischemia-causing stenoses obtained via noninvasive fractional flow reserve) study. J. Am. Coll. Cardiol..

[38] Huang X., Liu D., Yin X. (2018). Morphometry and hemodynamics of posterior communicating artery aneurysms: Ruptured versus unruptured. J. Biomech..

[39] Chen H.Y., Moussa I.D., Davidson C. (2012). Impact of main branch stenting on endothelial shear stress: Role of side branch diameter, angle and lesion. J. R. Soc. Interface.

[40] Mendieta J.B., Fontanarosa D., Wang J. (2023). MRI-based mechanical analysis of carotid atherosclerotic plaque using a material-property-mapping approach: A material-property-mapping method for plaque stress analysis. Comput. Methods Progr. Biomed..

[41] Hussain M., Lin D., Waqas H. (2025). An artificial intelligence and machine learning-driven CFD simulation for optimizing thermal performance of blood-integrated ternary nano-fluid. Eng. Appl. Comp. Fluid.

[42] Ozturk C., Pak D.H., Rosalia L. (2025). AI-powered multimodal modeling of personalized hemodynamics in aortic stenosis. Adv. Sci..

[43] Du P., An D., Wang C. (2025). AI-powered automated model construction for patient-specific CFD simulations of aortic flows. Sci. Adv..

[44] Majkut S., Dingal P.C.D.P., Discher D.E. (2014). Stress sensitivity and mechanotransduction during heart development. Curr. Biol..

[45] Wang M., Lin B.Y., Sun S. (2023). Shear and hydrostatic stress regulate fetal heart valve remodeling through YAP-mediated mechanotransduction. eLife.

[46] Gentile A., Albu M., Xu Y. (2024). Mechanical forces remodel the cardiac extracellular matrix during zebrafish development. Development.

[47] Lityagina O., Dobreva G. (2021). The linc between mechanical forces and chromatin. Front. Physiol..

[48] Ross J.A., Arcos-Villacis N., Battey E. (2023). Lem2 is essential for cardiac development by maintaining nuclear integrity. Cardiovasc. Res..

[49] Baillie J.S., Gendernalik A., Garrity D.M. (2023). The in vivo study of cardiac mechano-electric and mechano-mechanical coupling during heart development in zebrafish. Front. Physiol..

[50] Quinn T.A., Kohl P. (2021). Cardiac mechano-electric coupling: Acute effects of mechanical stimulation on heart rate and rhythm. Physiol. Rev..

[51] Chow R.W.Y., Fukui H., Chan W.X. (2022). Cardiac forces regulate zebrafish heart valve delamination by modulating nfat signaling. PLoS Biol..

[52] Zhong G., Su S., Li J. (2023). Activation of Piezo1 promotes osteogenic differentiation of aortic valve interstitial cell through YAP-dependent glutaminolysis. Sci. Adv..

[53] Adapala R.K., Katari V., Kanugula A.K. (2023). Deletion of endothelial TRPV4 protects heart from pressure overload-induced hypertrophy. Hypertension.

[54] Tachibana S., Yu N.K., Li R. (2023). Perm1 protects the heart from pressure overload-induced dysfunction by promoting oxidative metabolism. Circulation.

[55] Wang D., Yu X., Gao K. (2024). Sweroside alleviates pressure overload-induced heart failure through targeting CaMKIIδ to inhibit ROS-mediated NF-κB/NLRP3 in cardiomyocytes. Redox Biol..

[56] Liu X., Li B., Wang S. (2024). Stromal cell-SLIT3/cardiomyocyte-ROBO1 axis regulates pressure overload-induced cardiac hypertrophy. Circ. Res..

[57] Dayawansa N.H., Baratchi S., Peter K. (2022). Uncoupling the vicious cycle of mechanical stress and inflammation in calcific aortic valve disease. Front. Cardiovasc. Med..

[58] Bekedam F.T., Smal R., Smit M.C. (2024). Mechanical stimulation of induced pluripotent stem derived cardiac fibroblasts. Sci. Rep..

[59] Horii Y., Matsuda S., Toyota C. (2023). VGLL3 is a mechanosensitive protein that promotes cardiac fibrosis through liquid-liquid phase separation. Nat. Commun..

[60] Hu S., Chapski D.J., Gehred N.D. (2024). Histone H1.0 couples cellular mechanical behaviors to chromatin structure. Nat. Cardiovasc. Res..

[61] Whisler J., Shahreza S., Schlegelmilch K. (2023). Emergent mechanical control of vascular morphogenesis. Sci. Adv..

[62] Majesky M.W. (2018). Vascular development. Arterioscler. Thromb. Vasc. Biol..

[63] Yamamoto K., Nogimori Y., Imamura H. (2020). Shear stress activates mitochondrial oxidative phosphorylation by reducing plasma membrane cholesterol in vascular endothelial cells. Proc. Natl. Acad. Sci. USA.

[64] Davis M.J., Hill M.A. (1999). Signaling mechanisms underlying the vascular myogenic response. Physiol. Rev..

[65] Dillon P.F., Aksoy M.O., Driska S.P. (1981). Myosin phosphorylation and the cross-bridge cycle in arterial smooth-muscle. Science.

[66] Chiu J.J., Chien S. (2011). Effects of disturbed flow on vascular endothelium: Pathophysiological basis and clinical perspectives. Physiol. Rev..

[67] Costopoulos C., Timmins L.H., Huang Y. (2019). Impact of combined plaque structural stress and wall shear stress on coronary plaque progression, regression, and changes in composition. Eur. Heart J..

[68] Tziotziou A., Hartman E., Korteland S.A. (2023). Mechanical wall stress and wall shear stress are associated with atherosclerosis development in non-calcified coronary segments. Atherosclerosis.

[69] Wang X., Shen Y., Shang M. (2023). Endothelial mechanobiology in atherosclerosis. Cardiovasc. Res..

[70] Luo J.Y., Cheng C.K., He L. (2022). Endothelial UCP2 is a mechanosensitive suppressor of atherosclerosis. Circ. Res..

[71] Quan M., Lv H., Liu Z. (2022). MST1 suppresses disturbed flow induced atherosclerosis. Circ. Res..

[72] Li J., Zhu J., Gray O. (2024). Mechanosensitive super-enhancers regulate genes linked to atherosclerosis in endothelial cells. J. Cell Biol..

[73] Li J., Li X., Song S. (2023). Mitochondria spatially and temporally modulate VSMC phenotypes via interacting with cytoskeleton in cardiovascular diseases. Redox Biol..

[74] Pan H., Ho S.E., Xue C. (2024). Atherosclerosis is a smooth muscle cell-driven tumor-like disease. Circulation.

[75] Qian W., Hadi T., Silvestro M. (2022). Microskeletal stiffness promotes aortic aneurysm by sustaining pathological vascular smooth muscle cell mechanosensation via Piezo1. Nat. Commun..

[76] Herrera J.A., Schwartz M.A. (2022). MicroRNAs in mechanical homeostasis. Cold Spring Harb. Perspect. Med..

[77] Bao H., Li H.P., Shi Q. (2020). Lamin A/C negatively regulated by miR-124-3p modulates apoptosis of vascular smooth muscle cells during cyclic stretch application in rats. Acta Physiol..

[78] Huang K., Bao H., Yan Z.Q. (2017). Microrna-33 protects against neointimal hyperplasia induced by arterial mechanical stretch in the grafted vein. Cardiovasc. Res..

[79] Skalak T.C., Price R.J. (1996). The role of mechanical stresses in microvascular remodeling. Microcirculation.

[80] Harraz O.F., Klug N.R., Senatore A.J. (2022). Piezo1 is a mechanosensor channel in central nervous system capillaries. Circ. Res..

[81] Gouveia B., Kim Y., Shaevitz J.W. (2022). Capillary forces generated by biomolecular condensates. Nature.

[82] Beitler J.R., Malhotra A., Thompson B.T. (2016). Ventilator-induced lung injury. Clin. Chest Med..

[83] Cash A., Theus M.H. (2020). Mechanisms of blood-brain barrier dysfunction in traumatic brain injury. Int. J. Mol. Sci..

[84] Srivastava T., Thiagarajan G., Alon U.S. (2017). Role of biomechanical forces in hyperfiltration-mediated glomerular injury in congenital anomalies of the kidney and urinary tract. Nephrol. Dial. Transplant..

[85] Fung Y.-C. (1993).

[86] Chien S. (2007). Mechanotransduction and endothelial cell homeostasis: The wisdom of the cell. Am. J. Physiol. Heart Circ. Physiol..

[87] Mehta P.D., Narayanan R.O., Kulkarni V. (2025). Hybridcollab: Unifying in-person and remote collaboration for cardiovascular surgical planning in mobile augmented reality. arXiv.

[88] Ru Q., Li Y., Xie W. (2023). Fighting age-related orthopedic diseases: Focusing on ferroptosis. Bone Res..

[89] Xiao Z., Quarles L.D. (2015). Physiological mechanisms and therapeutic potential of bone mechanosensing. Rev. Endocr. Metab. Disord..

[90] Brown G.N., Sattler R.L., Guo X.E. (2016). Experimental studies of bone mechanoadaptation: Bridging in vitro and in vivo studies with multiscale systems. Interface Focus.

[91] Hodgkinson T., Kelly D.C., Curtin C.M. (2022). Mechanosignalling in cartilage: An emerging target for the treatment of osteoarthritis. Nat. Rev. Rheumatol..

[92] Gao L., Liu D., Gao H. (2019). Effects of creep and creep-recovery on ratcheting strain of articular cartilage under cyclic compression. Mater. Sci. Eng., C.

[93] Gao L., Feng L., Tan Y. (2023). Effect of biaxial cyclic loading path on the mechanical and microstructure properties of articular cartilage. Mech. Mater..

[94] Si Y., Tan Y., Gao L. (2022). Mechanical properties of cracked articular cartilage under uniaxial creep and cyclic tensile loading. J. Biomech..

[95] Yang X., Li S., Ren Y. (2022). 3D printed hydrogel for articular cartilage regeneration. Compos. Pt. B-Eng..

[96] Starodubtseva M.N. (2011). Mechanical properties of cells and ageing. Ageing Res. Rev..

[97] Trickey W.R., Vail T.P., Guilak F. (2004). The role of the cytoskeleton in the viscoelastic properties of human articular chondrocytes. J. Orthop. Res..

[98] Liu C., Zhao Y., Cheung W.Y. (2010). Effects of cyclic hydraulic pressure on osteocytes. Bone.

[99] Qin L., Liu W., Cao H. (2020). Molecular mechanosensors in osteocytes. Bone Res..

[100] Wang L., You X., Zhang L. (2022). Mechanical regulation of bone remodeling. Bone Res..

[101] Kopperdahl D.L., Aspelund T., Hoffmann P.F. (2014). Assessment of incident spine and hip fractures in women and men using finite element analysis of CT scans. J. Bone Miner. Res..

[102] Rajapakse C.S., Hotca A., Newman B.T. (2017). Patient-specific hip fracture strength assessment with microstructural MR imaging-based finite element modeling. Radiology.

[103] Gao X., Din R.U., Cheng X. (2023). Biomechanical MRI detects reduced bone strength in subjects with vertebral fractures. Bone.

[104] Zhang M., Gong H., Zhang K. (2019). Prediction of lumbar vertebral strength of elderly men based on quantitative computed tomography images using machine learning. Osteoporos. Int..

[105] Zhang M., Gong H., Zhang M. (2023). Prediction of femoral strength of elderly men based on quantitative computed tomography images using machine learning. J. Orthop. Res..

[106] Liu S., Zhang M., Gong H. (2025). Explainable machine-learning-based prediction of QCT/FEA-calculated femoral strength under stance loading configuration using radiomics features. J. Orthop. Res..

[107] Klein-Nulend J., Bakker A.D., Bacabac R.G. (2013). Mechanosensation and transduction in osteocytes. Bone.

[108] Verbruggen S.W., Vaughan T.J., McNamara L.M. (2012). Strain amplification in bone mechanobiology: A computational investigation of the in vivo mechanics of osteocytes. J. R. Soc. Interface.

[109] Joukar A., Niroomand-Oscuii H., Ghalichi F. (2016). Numerical simulation of osteocyte cell in response to directional mechanical loadings and mechanotransduction analysis: Considering lacunar-canalicular interstitial fluid flow. Comput. Methods Progr. Biomed..

[110] Cen H., Yao Y., Liu H. (2021). Multiscale mechanical responses of young and elderly human femurs: A finite element investigation. Bone.

[111] Gupta A., Saha S., Das A. (2024). Evaluating the influence on osteocyte mechanobiology within the lacunar-canalicular system for varying lacunar equancy and perilacunar elasticity: A multiscale fluid-structure interaction analysis. J. Mech. Behav. Biomed. Mater..

[112] Awal R., Naznin M., Faisal T.R. (2025). Machine learning based finite element analysis (FEA) surrogate for hip fracture risk assessment and visualization. Expert Syst. Appl..

[113] Synek A., Benca E., Licandro R. (2025). Predicting strength of femora with metastatic lesions from single 2D radiographic projections using convolutional neural networks. Comput. Methods Progr. Biomed..

[114] Pais A., Alves J.L., Belinha J. (2023). Predicting trabecular arrangement in the proximal femur: An artificial neural network approach for varied geometries and load cases. J. Biomech..

[115] Liu X., Miramini S., Patel M. (2023). Development of numerical model-based machine learning algorithms for different healing stages of distal radius fracture healing. Comput. Methods Progr. Biomed..

[116] Jin J., Bakker A.D., Wu G. (2019). Physicochemical niche conditions and mechanosensing by osteocytes and myocytes. Curr. Osteoporos. Rep..

[117] Stefaan W., Verbruggen L.M.M., Verbruggen S.W. (2018). Mechanobiology in Health and Disease.

[118] Wang L., You X., Lotinun S. (2020). Mechanical sensing protein Piezo1 regulates bone homeostasis via osteoblast-osteoclast crosstalk. Nat. Commun..

[119] Li X., Han L., Nookaew I. (2019). Stimulation of Piezo1 by mechanical signals promotes bone anabolism. eLife.

[120] Li Y., Yang Y., Wang X. (2025). Extracellular osmolarity regulates osteoblast migration through the TRPV4-Rho/ROCK signaling. Commun. Biol..

[121] Verbruggen S.W., Vaughan T.J., McNamara L.M. (2016). Mechanisms of osteocyte stimulation in osteoporosis. J. Mech. Behav. Biomed. Mater..

[122] Verbruggen S.W., Mc Garrigle M.J., Haugh M.G. (2015). Altered mechanical environment of bone cells in an animal model of short- and long-term osteoporosis. Biophys. J..

[123] Yeh C.R., Chiu J.J., Lee C.I. (2010). Estrogen augments shear stress-induced signaling and gene expression in osteoblast-like cells via estrogen receptor-mediated expression of β_1_-integrin. J. Bone Miner. Res..

[124] Deepak V., Kayastha P., McNamara L.M. (2017). Estrogen deficiency attenuates fluid flow-induced [Ca^2+^]_i_ oscillations and mechanoresponsiveness of MLO-Y4 osteocytes. FASEB J..

[125] Damien E., Price J.S., Lanyon L.E. (1998). The estrogen receptor's involvement in osteoblasts' adaptive response to mechanical strain. J. Bone Miner. Res..

[126] Lee W., Nims R.J., Savadipour A. (2021). Inflammatory signaling sensitizes Piezo1 mechanotransduction in articular chondrocytes as a pathogenic feed-forward mechanism in osteoarthritis. Proc. Natl. Acad. Sci. USA.

[127] Luu A.K., Viloria-Petit A.M. (2020). Targeting mechanotransduction in osteosarcoma: A comparative oncology perspective. Int. J. Mol. Sci..

[128] Shoaib Z., Fan T.M., Irudayaraj J.M.K. (2022). Osteosarcoma mechanobiology and therapeutic targets. Br. J. Pharmacol..

[129] Urciuoli E., Peruzzi B. (2020). Involvement of the FAK network in pathologies related to altered mechanotransduction. Int. J. Mol. Sci..

[130] Mathavan N., Singh A., Marques F.C. (2025). Spatial transcriptomics in bone mechanomics: Exploring the mechanoregulation of fracture healing in the era of spatial omics. Sci. Adv..

[131] Coveney C.R., Capellini T.D. (2025). Mechanoepigenetics in musculoskeletal disease. Osteoarthr. Cartil..

[132] Chen L., Huang Y., Zhang X. (2023). Corneal biomechanical properties demonstrate anisotropy and correlate with axial length in myopic eyes. Investig. Ophthalmol. Vis. Sci..

[133] Cehelyk E.K., Syed Z.A. (2024). Long-term outcomes of corneal crosslinking. Curr. Opin. Ophthalmol..

[134] Yang M., Pan H., Chen T. (2024). Customized corneal cross-linking with microneedle-mediated riboflavin delivery for keratoconus treatment. Adv. Mater..

[135] Alkharashi M.S., Al-Essa R.S., Abusayf M.M. (2023). Three-years outcomes of simultaneous photorefractive surgery and customized corneal cross-linking for keratoconus. Int. Ophthalmol..

[136] Boote C., Sigal I.A., Grytz R. (2020). Scleral structure and biomechanics. Prog. Retin. Eye Res..

[137] Zhang F., Lai L. (2021). Advanced research in scleral cross-linking to prevent from progressive myopia. Asia. Pac. J. Ophthalmol..

[138] Yasir Z.H., Sharma R., Zakir S.M. (2024). Scleral collagen cross linkage in progressive myopia. Indian J. Ophthalmol..

[139] Li Y., Qi Y., Sun M. (2023). Clinical feasibility and safety of scleral collagen cross-linking by riboflavin and ultraviolet a in pathological myopia blindness: A pilot study. Ophthalmol. Ther..

[140] Li Y., Yang Z., Yan Z. (2025). Revealing and predicting the long-term biomechanical response of orthokeratology by developing a patient-specific computational model. Sci. China Phys. Mech. Astron..

[141] Li F., Wang K., Liu Z. (2023). In vivo biomechanical measurements of the cornea. Bioengineering-Basel.

[142] Zhang H., Eliasy A., Lopes B. (2021). Stress-strain index map: A new way to represent corneal material stiffness. Front. Bioeng. Biotechnol..

[143] Lopes B.T., Elsheikh A. (2023). In vivo corneal stiffness mapping by the stress-strain index maps and brillouin microscopy. Curr. Eye Res..

[144] Li R., Qian X., Gong C. (2023). Simultaneous assessment of the whole eye biomechanics using ultrasonic elastography. IEEE Trans. Biomed. Eng..

[145] Desouky N.A., Saafan M.M., Mansour M.H. (2023). Patient-specific air puff-induced loading using machine learning. Front. Bioeng. Biotechnol..

[146] Thomasy S.M., Leonard B.C., Greiner M.A. (2024). Squishy matters - corneal mechanobiology in health and disease. Prog. Retin. Eye Res..

[147] Chen J., Mo Q., Sheng R. (2023). Stiffness-dependent dynamic effect of inflammation on keratocyte phenotype and differentiation. Biomed. Mater..

[148] Mozzer A., Pitha I. (2024). Cyclic strain alters the transcriptional and migratory response of scleral fibroblasts to TGFβ. Exp. Eye Res..

[149] Sun Y., Sha Y., Yang J. (2024). Collagen is crucial target protein for scleral remodeling and biomechanical change in myopia progression and control. Heliyon.

[150] Xie Y., Ouyang X., Wang G. (2020). Mechanical strain affects collagen metabolism-related gene expression in scleral fibroblasts. Biomed. Pharmacother..

[151] Liu X., Yuan Y., Wu Y. (2025). Extracellular matrix stiffness modulates myopia scleral remodeling through integrin/F-actin/YAP axis. Investig. Ophthalmol. Vis. Sci..

[152] Kedar S., Tong J., Bader J. (2022). Effects of acute intracranial pressure changes on optic nerve head morphology in humans and pig model. Curr. Eye Res..

[153] Li L., Song F. (2020). Biomechanical research into lamina cribrosa in glaucoma. Natl. Sci. Rev..

[154] Grytz R., Girkin C.A., Libertiaux V. (2012). Perspectives on biomechanical growth and remodeling mechanisms in glaucoma. Mech. Res. Commun..

[155] Tan N.Y., Koh V., Girard M.J. (2018). Imaging of the lamina cribrosa and its role in glaucoma: A review. Clin. Exp. Ophthalmol..

[156] Tian H., Li L., Song F. (2017). Study on the deformations of the lamina cribrosa during glaucoma. Acta Biomater..

[157] Causin P., Guidoboni G., Harris A. (2014). A poroelastic model for the perfusion of the lamina cribrosa in the optic nerve head. Math. Biosci..

[158] Guidoboni G., Harris A., Cassani S. (2014). Intraocular pressure, blood pressure, and retinal blood flow autoregulation: A mathematical model to clarify their relationship and clinical relevance. Investig. Ophthalmol. Vis. Sci..

[159] Karimi A., Rahmati S.M., Grytz R.G. (2021). Modeling the biomechanics of the lamina cribrosa microstructure in the human eye. Acta Biomater..

[160] Voorhees A.P., Jan N.J., Austin M.E. (2017). Lamina cribrosa pore shape and size as predictors of neural tissue mechanical insult. Investig. Ophthalmol. Vis. Sci..

[161] Roberts M.D., Grau V., Grimm J. (2009). Remodeling of the connective tissue microarchitecture of the lamina cribrosa in early experimental glaucoma. Investig. Ophthalmol. Vis. Sci..

[162] Wallace D.M., O'Brien C.J. (2016). The role of lamina cribrosa cells in optic nerve head fibrosis in glaucoma. Exp. Eye Res..

[163] Tovar-Vidales T., Wordinger R.J., Clark A.F. (2016). Identification and localization of lamina cribrosa cells in the human optic nerve head. Exp. Eye Res..

[164] Schneider M., Fuchshofer R. (2016). The role of astrocytes in optic nerve head fibrosis in glaucoma. Exp. Eye Res..

[165] Irnaten M., Duff A., Clark A. (2020). Intra-cellular calcium signaling pathways (PKC, RAS/RAF/MAPK, PI3K) in lamina cribrosa cells in glaucoma. J. Clin. Med..

[166] Murphy R., Irnaten M., Hopkins A. (2022). Matrix mechanotransduction via yes-associated protein in human lamina cribrosa cells in glaucoma. Investig. Ophthalmol. Vis. Sci..

[167] Irnaten M., Gaynor E., O'Brien C. (2024). The role of αvβ3 integrin in lamina cribrosa cell mechanotransduction in glaucoma. Cells.

[168] Irnaten M., O'Brien C.J. (2023). Calcium-signalling in human glaucoma lamina cribrosa myofibroblasts. Int. J. Mol. Sci..

[169] Quill B., Irnaten M., Docherty N.G. (2015). Calcium channel blockade reduces mechanical strain-induced extracellular matrix gene response in lamina cribrosa cells. Br. J. Ophthalmol..

[170] Kirwan R.P., Fenerty C.H., Crean J. (2005). Influence of cyclical mechanical strain on extracellular matrix gene expression in human lamina cribrosa cells in vitro. Mol. Vis..

[171] Hopkins A.A., Murphy R., Irnaten M. (2020). The role of lamina cribrosa tissue stiffness and fibrosis as fundamental biomechanical drivers of pathological glaucoma cupping. Am. J. Physiol. Cell Physiol..

[172] Kapuganti R.S., Alone D.P. (2023). Current understanding of genetics and epigenetics in pseudoexfoliation syndrome and glaucoma. Mol. Aspect. Med..

[173] Li G., Lee C., Agrahari V. (2019). In vivo measurement of trabecular meshwork stiffness in a corticosteroid-induced ocular hypertensive mouse model. Proc. Natl. Acad. Sci. USA.

[174] Last J.A., Pan T., Ding Y. (2011). Elastic modulus determination of normal and glaucomatous human trabecular meshwork. Investig. Ophthalmol. Vis. Sci..

[175] Karimi A., Rahmati S.M., Razaghi R. (2022). Biomechanics of human trabecular meshwork in healthy and glaucoma eyes via dynamic schlemm's canal pressurization. Comput. Methods Progr. Biomed..

[176] Camras L.J., Stamer W.D., Epstein D. (2014). Differential effects of trabecular meshwork stiffness on outflow facility in normal human and porcine eyes. Investig. Ophthalmol. Vis. Sci..

[177] Wang K., Li G., Read A.T. (2018). The relationship between outflow resistance and trabecular meshwork stiffness in mice. Sci. Rep..

[178] Karimi A., Aga M., Khan T. (2024). Dynamic traction force in trabecular meshwork cells: A 2D culture model for normal and glaucomatous states. Acta Biomater..

[179] Carreon T., van der Merwe E., Fellman R.L. (2017). Aqueous outflow - a continuum from trabecular meshwork to episcleral veins. Prog. Retin. Eye Res..

[180] Yarishkin O., Phuong T.T.T., Baumann J.M. (2021). Piezo1 channels mediate trabecular meshwork mechanotransduction and promote aqueous fluid outflow. J. Physiol..

[181] Zhu W., Hou F., Fang J. (2021). The role of Piezo1 in conventional aqueous humor outflow dynamics. iScience.

[182] Morozumi W., Aoshima K., Inagaki S. (2021). Piezo 1 is involved in intraocular pressure regulation. J. Pharmacol. Sci..

[183] Patel P.D., Chen Y.L., Kasetti R.B. (2021). Impaired TRPV4-eNOS signaling in trabecular meshwork elevates intraocular pressure in glaucoma. Proc. Natl. Acad. Sci. USA.

[184] Du R., Li D., Zhu M. (2022). Cell senescence alters responses of porcine trabecular meshwork cells to shear stress. Front. Cell Dev. Biol..

[185] Acott T.S., Vranka J.A., Keller K.E. (2021). Normal and glaucomatous outflow regulation. Prog. Retin. Eye Res..

[186] Yemanyi F., Vranka J., Raghunathan V.K. (2020). Crosslinked extracellular matrix stiffens human trabecular meshwork cells via dysregulating β-catenin and YAP/TAZ signaling pathways. Investig. Ophthalmol. Vis. Sci..

[187] Li H., Raghunathan V., Stamer W.D. (2022). Extracellular matrix stiffness and TGFβ2 regulate YAP/TAZ activity in human trabecular meshwork cells. Front. Cell Dev. Biol..

[188] Zhang R., Li B., Li H. (2023). Extracellular-matrix mechanics regulate the ocular physiological and pathological activities. J. Ophthalmol..

[189] Ghosh R., Herberg S. (2024). The role of YAP/TAZ mechanosignaling in trabecular meshwork and schlemm's canal cell dysfunction. Vis. Res..

[190] Xu J., Liu K., Wang F. (2025). The TRPV4-YAP axis mediates cytoskeletal and extracellular matrix remodeling in trabecular meshwork cells as a novel glaucoma mechanism. Sci. Rep..

[191] Li N., Zhang X., Zhou J. (2022). Multiscale biomechanics and mechanotransduction from liver fibrosis to cancer. Adv. Drug Deliv. Rev..

[192] Du Y., Li N., Yang H. (2017). Mimicking liver sinusoidal structures and functions using a 3D-configured microfluidic chip. Lab Chip.

[193] Chen S., Zhu J., Xue J. (2022). Numerical simulation of flow characteristics in a permeable liver sinusoid with leukocytes. Biophys. J..

[194] Wu Y., Li N., Shu X. (2023). Biomechanics in liver regeneration after partial hepatectomy. Front. Bioeng. Biotechnol..

[195] Zhang X., Li P., Zhou J. (2024). FAK-p38 signaling serves as a potential target for reverting matrix stiffness-modulated liver sinusoidal endothelial cell defenestration. Biomaterials.

[196] Li P., Zhou J., Li W. (2020). Characterizing liver sinusoidal endothelial cell fenestrae on soft substrates upon AFM imaging and deep learning. Biochim. Biophys. Acta Gen. Subj..

[197] Gola A., Dorrington M.G., Speranza E. (2021). Commensal-driven immune zonation of the liver promotes host defence. Nature.

[198] Yang H., Li N., Du Y. (2017). Neutrophil adhesion and crawling dynamics on liver sinusoidal endothelial cells under shear flow. Exp. Cell Res..

[199] Ye J., Li F., Hua T. (2024). Liver mechanosignaling as a natural anti-hepatitis B virus mechanism. Nat. Commun..

[200] Mitten E.K., Baffy G. (2022). Mechanotransduction in the pathogenesis of non-alcoholic fatty liver disease. J. Hepatol..

[201] Shu X., Li N., Wu Y. (2021). Mechanotransduction of liver sinusoidal endothelial cells under varied mechanical stimuli. Acta Mech. Sin..

[202] Tong C.F., Zhang Y., Lü S.Q. (2018). Binding of intercellular adhesion molecule 1 to β_2_-integrin regulates distinct cell adhesion processes on hepatic and cerebral endothelium. Am. J. Physiol. Cell Physiol..

[203] Mattei G., Ahluwalia A. (2016). Sample, testing and analysis variables affecting liver mechanical properties: A review. Acta Biomater..

[204] Kemper A.R., Santago A.C., Stitzel J.D. (2010). Biomechanical response of human liver in tensile loading. Ann. Adv. Automot. Med..

[205] Untaroiu C.D., Lu Y.C., Siripurapu S.K. (2015). Modeling the biomechanical and injury response of human liver parenchyma under tensile loading. J. Mech. Behav. Biomed. Mater..

[206] Yang Z., Simon R., Merrell K. (2025). Boundary constraint-free biomechanical model-based surface matching for intraoperative liver deformation correction. IEEE Trans. Med. Imag..

[207] Zhu J., Su Y., Liu Z. (2022). Real-time biomechanical modelling of the liver using LightGBM model. Int. J. Med. Robot..

[208] Hu Z., Liao S., Kui X. (2025). Real-time integrated modeling of soft tissue deformation and stress based on deep learning. Phys. Med. Biol..

[209] Mueller S., Sandrin L. (2010). Liver stiffness: A novel parameter for the diagnosis of liver disease. Hepat. Med..

[210] Georges P.C., Hui J.J., Gombos Z. (2007). Increased stiffness of the rat liver precedes matrix deposition: Implications for fibrosis. Am. J. Physiol. Gastrointest. Liver Physiol..

[211] Guixé-Muntet S., Ortega-Ribera M., Wang C. (2020). Nuclear deformation mediates liver cell mechanosensing in cirrhosis. JHEP Rep..

[212] Desai S.S., Tung J.C., Zhou V.X. (2016). Physiological ranges of matrix rigidity modulate primary mouse hepatocyte function in part through hepatocyte nuclear factor 4 alpha. Hepatology.

[213] Olsen A.L., Bloomer S.A., Chan E.P. (2011). Hepatic stellate cells require a stiff environment for myofibroblastic differentiation. Am. J. Physiol. Gastrointest. Liver Physiol..

[214] Jain I., Brougham-Cook A., Underhill G.H. (2023). Effect of distinct ECM microenvironments on the genome-wide chromatin accessibility and gene expression responses of hepatic stellate cells. Acta Biomater..

[215] Zhao W., Yuan W., Dong T. (2025). Increased matrix stiffness promotes fibrogenesis of hepatic stellate cells through AP-1-induced chromatin priming. Commun. Biol..

[216] Fan W., Adebowale K., Váncza L. (2024). Matrix viscoelasticity promotes liver cancer progression in the pre-cirrhotic liver. Nature.

[217] Loneker A.E., Alisafaei F., Kant A. (2023). Lipid droplets are intracellular mechanical stressors that impair hepatocyte function. Proc. Natl. Acad. Sci. USA.

[218] Ivanovska I.L., Tobin M.P., Bai T. (2023). Small lipid droplets are rigid enough to indent a nucleus, dilute the lamina, and cause rupture. J. Cell Biol..

[219] Greuter T., Yaqoob U., Gan C. (2022). Mechanotransduction-induced glycolysis epigenetically regulates a CXCL1-dominant angiocrine signaling program in liver sinusoidal endothelial cells in vitro and in vivo. J. Hepatol..

[220] Liu L., You Z., Yu H. (2017). Mechanotransduction-modulated fibrotic microniches reveal the contribution of angiogenesis in liver fibrosis. Nat. Mater..

[221] Gracia-Sancho J., Caparrós E., Fernández-Iglesias A. (2021). Role of liver sinusoidal endothelial cells in liver diseases. Nat. Rev. Gastroenterol. Hepatol..

[222] Abshagen K., Eipel C., Kalff J.C. (2008). Kupffer cells are mandatory for adequate liver regeneration by mediating hyperperfusion via modulation of vasoactive proteins. Microcirculation.

[223] Ishikawa J., Takeo M., Iwadate A. (2021). Mechanical homeostasis of liver sinusoid is involved in the initiation and termination of liver regeneration. Commun. Biol..

[224] Duan J.L., Ruan B., Song P. (2022). Shear stress-induced cellular senescence blunts liver regeneration through Notch-sirtuin 1-P21/P16 axis. Hepatology.

[225] Wu Y., Li L., Li W. (2025). Stretch-induced hepatic endothelial mechanocrine promotes hepatocyte proliferation. Hepatology.

[226] Lorenz L., Axnick J., Buschmann T. (2018). Mechanosensing by β1 integrin induces angiocrine signals for liver growth and survival. Nature.

[227] Hilscher M.B., Sehrawat T., Arab J.P. (2019). Mechanical stretch increases expression of CXCL1 in liver sinusoidal endothelial cells to recruit neutrophils, generate sinusoidal microthombi, and promote portal hypertension. Gastroenterology.

[228] Li W., Wu Y., Hu W. (2023). Direct mechanical exposure initiates hepatocyte proliferation. JHEP Rep..

[229] Li W., Li P., Li N. (2021). Matrix stiffness and shear stresses modulate hepatocyte functions in a fibrotic liver sinusoidal model. Am. J. Physiol. Gastrointest. Liver Physiol..

[230] Du Y., de Jong I.E.M., Gupta K. (2023). Human vascularized bile duct-on-a chip: A multi-cellular micro-physiological system for studying cholestatic liver disease. Biofabrication.

[231] Du Y., Khandekar G., Llewellyn J. (2020). A bile duct-on-a-chip with organ-level functions. Hepatology.

[232] Rizwan M., Ling C., Guo C. (2022). Viscoelastic Notch signaling hydrogel induces liver bile duct organoid growth and morphogenesis. Adv. Healthcare Mater..

[233] Sorrentino G., Rezakhani S., Yildiz E. (2020). Mechano-modulatory synthetic niches for liver organoid derivation. Nat. Commun..

[234] Basil M.C., Katzen J., Engler A.E. (2020). The cellular and physiological basis for lung repair and regeneration: Past, present, and future. Cell Stem Cell.

[235] Kim H., Yi S., Liyanage P. (2025). Development of a 3D bioengineered human lung submucosal gland ductal airway model to study mucociliary clearance in vitro. Cell Biomater..

[236] Liu L., Stephens B., Bergman M. (2021). Role of collagen in airway mechanics. Bioengineering-Basel.

[237] Vindin H.J., Oliver B.G., Weiss A.S. (2022). Elastin in healthy and diseased lung. Curr. Opin. Biotechnol..

[238] Souza-Fernandes A.B., Pelosi P., Rocco P.R.M. (2006). Bench-to-bedside review: The role of glycosaminoglycans in respiratory disease. Crit. Care.

[239] Zhou X., Wu Y., Zhu Z. (2025). Mucosal immune response in biology, disease prevention and treatment. Signal Transduct. Targeted Ther..

[240] Lin L., Pou Casellas C., Dost A.F.M. (2025). Human airway submucosal gland organoids to study respiratory inflammation and infection. Cell Stem Cell.

[241] Stenmark K.R., Nozik-Grayck E., Gerasimovskaya E. (2011). The adventitia: Essential role in pulmonary vascular remodeling. Compr. Physiol..

[242] Li R., Li J., Zhou X. (2024). Lung microbiome: New insights into the pathogenesis of respiratory diseases. Signal Transduct. Targeted Ther..

[243] Abdo M., Trinkmann F., Kirsten A.M. (2021). Small airway dysfunction links asthma severity with physical activity and symptom control. J. Allergy Clin. Immunol. Pract..

[244] Lan B., Norris B.A., Liu J.C.Y. (2015). Development and maintenance of force and stiffness in airway smooth muscle. Can. J. Physiol. Pharmacol..

[245] Yang C., Guo J., Ni K. (2023). Mechanical ventilation-related high stretch mainly induces endoplasmic reticulum stress and thus mediates inflammation response in cultured human primary airway smooth muscle cells. Int. J. Mol. Sci..

[246] Frerichs I., Lasarow L., Strodthoff C. (2021). Spatial ventilation inhomogeneity determined by electrical impedance tomography in patients with chronic obstructive lung disease. Front. Physiol..

[247] He A., He L., Chen T. (2024). Biomechanical properties and cellular responses in pulmonary fibrosis. Bioengineering-Basel.

[248] Nho R.S., Ballinger M.N., Rojas M.M. (2022). Biomechanical force and cellular stiffness in lung fibrosis. Am. J. Pathol..

[249] Wang J., Li K., Hao D. (2024). Pulmonary fibrosis: Pathogenesis and therapeutic strategies. MedComm.

[250] Lancmanová A., Bodnár T. (2025). Numerical simulations of human respiratory flows: A review. Discov. Appl. Sci..

[251] Ou X., Meng J., Ma C. (2025). Numerical simulation of voluntary respiration in a model of the whole human lower airway. Biomech. Model. Mechanobiol..

[252] Emmerling J., Vahaji S., Morton D.A.V. (2024). Scale resolving simulations of the effect of glottis motion and the laryngeal jet on flow dynamics during respiration. Comput. Methods Progr. Biomed..

[253] Jin Y., Liu Z., Hu C. (2024). Study on the flow mechanism and frequency characteristics of rales in lower respiratory tract. Biomech. Model. Mechanobiol..

[254] Tsega E.G., Katiyar V.K. (2019). Numerical simulations of inspiratory airflow in healthy and asthmatic human airways. Am. J. Biomed. Eng..

[255] Johnsen S.G. (2024). Computational rhinology: Unraveling discrepancies between in silico and in vivo nasal airflow assessments for enhanced clinical decision support. Bioengineering-Basel.

[256] Moshksayan K., Bahmanzadeh H., Faramarzi M. (2022). In-silico investigation of airflow and micro-particle deposition in human nasal airway pre- and post-virtual transnasal sphenoidotomy surgery. Comput. Methods Biomech. Biomed. Eng..

[257] Hu S., Jin Q., Gao C. (2025). The new paradigm of computational fluid dynamics: Empowering computational fluid dynamics with machine learning. Phys. Fluids.

[258] Talaat M., Si X., Dong H. (2025). Physics-informed neural networks simulation and validation of airflows in three-dimensional upper respiratory tracts. Fluid.

[259] Bagley D.C., Russell T., Ortiz-Zapater E. (2024). Bronchoconstriction damages airway epithelia by crowding-induced excess cell extrusion. Science.

[260] Drazen J.M., Fredberg J.J. (2024). Epithelial cells crowded out in asthma. Science.

[261] Jairaman A., Prakriya M. (2024). Calcium signaling in airway epithelial cells: Current understanding and implications for inflammatory airway disease. Arterioscler. Thromb. Vasc. Biol..

[262] Luo M., Ni K., Jin Y. (2019). Toward the identification of extra-oral TAS2R agonists as drug agents for muscle relaxation therapies via bioinformatics-aided screening of bitter compounds in traditional chinese medicine. Front. Physiol..

[263] Luo M., Yu P., Ni K. (2020). Sanguinarine rapidly relaxes rat airway smooth muscle cells dependent on TAS2R signaling. Biol. Pharm. Bull..

[264] Ni K., Guo J., Bu B. (2021). Naringin as a plant-derived bitter tastant promotes proliferation of cultured human airway epithelial cells via activation of TAS2R signaling. Phytomedicine.

[265] Lüchtefeld I., Pivkin I.V., Gardini L. (2024). Dissecting cell membrane tension dynamics and its effect on Piezo1-mediated cellular mechanosensitivity using force-controlled nanopipettes. Nat. Methods.

[266] Luo M., Ni K., Gu R. (2023). Chemical activation of Piezo1 alters biomechanical behaviors toward relaxation of cultured airway smooth muscle cells. Biol. Pharm. Bull..

[267] Luo M., Zhang X., Guo J. (2025). Piezo1 agonist Yoda1 induces rapid relaxation in cultured airway smooth muscle cells and bronchodilation in mouse models. Am. J. Respir. Cell Mol. Biol..

[268] Diem K., Fauler M., Fois G. (2020). Mechanical stretch activates piezo1 in caveolae of alveolar type I cells to trigger ATP release and paracrine stimulation of surfactant secretion from alveolar type II cells. FASEB J..

[269] Liu H., Hu J., Zheng Q. (2022). Piezo1 channels as force sensors in mechanical force-related chronic inflammation. Front. Immunol..

[270] Xu L.R., Li T., Cao Y.P. (2025). Piezo1 mediates periostin myofibroblast activation and pulmonary fibrosis in mice. J. Clin. Investig..

[271] Cosgrove B.D., Bounds L.R., Taylor C.K. (2024). Mechanosensitive genomic enhancers potentiate the cellular response to matrix stiffness. bioRxiv.

[272] Chen J. (2009). Food oral processing - a review. Food Hydrocoll..

[273] Schwartz M.A. (2009). The force is with us. Science.

[274] Li Y., Zhan Q., Bao M. (2021). Biomechanical and biological responses of periodontium in orthodontic tooth movement: Up-date in a new decade. Int. J. Oral Sci..

[275] Nam N.E., Hwangbo N.K., Kim J.E. (2024). Effects of surface glazing on the mechanical and biological properties of 3D printed permanent dental resin materials. J. Prosthodont. Res..

[276] Ho S.P., Kurylo M.P., Fong T.K. (2010). The biomechanical characteristics of the bone-periodontal ligament-cementum complex. Biomaterials.

[277] Gasik M., Lambert F., Bacevic M. (2021). Biomechanical properties of bone and mucosa for design and application of dental implants. Materials.

[278] Katona T.R., Eckert G.J. (2017). The mechanics of dental occlusion and disclusion. Clin. Biomech..

[279] Ramalingam S., Sundar C., Jansen J.A., Alghamdi H., Jansen J. (2022). Dental Implants and Bone Grafts.

[280] Alibrahim A. (2015).

[281] Heckmann J.G., Urban P.P., Pitz S. (2019). The diagnosis and treatment of idiopathic facial paresis (Bell's palsy). Dtsch. Arztebl. Int..

[282] Buschang P.H., Jacob H., Carrillo R. (2013). The morphological characteristics, growth, and etiology of the hyperdivergent phenotype. Semin. Orthod..

[283] Costa Y.M., Ariji Y., Ferreira D.M.A.O. (2018). Muscle hardness and masticatory myofascial pain: Assessment and clinical relevance. J. Oral Rehabil..

[284] Chen Y.J., Lin H.Y., Chu C.A. (2023). Assessing thickness and stiffness of superficial/deep masticatory muscles in orofacial pain: An ultrasound and shear wave elastography study. Ann. Med..

[285] Khan A., Khan S., Kim Y.S. (2019). Insight into pain modulation: Nociceptors sensitization and therapeutic targets. Curr. Drug Targets.

[286] Penasso H., Petersen F., Peternell G. (2023). Vascular and neural response to focal vibration, sensory feedback, and piezo ion channel signaling. J. Vasc. Dis..

[287] Di C., Jia W. (2024). Food-derived bioactive peptides as momentous food components: Can functional peptides passed through the PI3K/Akt/mTOR pathway and NF-κB pathway to repair and protect the skeletal muscle injury?. Crit. Rev. Food Sci. Nutr..

[288] Liu M., Huang X., Tian Y. (2020). Phosphorylated GSK-3β protects stress-induced apoptosis of myoblasts via the PI3K/Akt signaling pathway. Mol. Med. Rep..

[289] Yeom B., Sain T., Lacevic N. (2017). Abiotic tooth enamel. Nature.

[290] Zhao H., Liu S., Wei Y. (2022). Multiscale engineered artificial tooth enamel. Science.

[291] Lei C., Wang K.Y., Ma Y.X. (2024). Biomimetic self-maturation mineralization system for enamel repair. Adv. Mater..

[292] Li Y., Kong Y., Xue B. (2022). Mechanically reinforced artificial enamel by Mg^2+^-induced amorphous intergranular phases. ACS Nano.

[293] Wei Y., Liu S., Xiao Z. (2020). Enamel repair with amorphous ceramics. Adv. Mater..

[294] Liu N., Zhou M., Zhang Q. (2018). Stiffness regulates the proliferation and osteogenic/odontogenic differentiation of human dental pulp stem cells via the WNT signalling pathway. Cell Prolif..

[295] Bryniarska-Kubiak N., Basta-Kaim A., Kubiak A. (2024). Mechanobiology of dental pulp cells. Cells.

[296] Zhang Z., Li C., Guo J. (2024). "Young-mechanical niche" biomimetic hydrogel promotes dental pulp regeneration through YAP-dependent mechanotransduction. Chem. Eng. J..

[297] Stanton A.E., Tong X., Yang F. (2019). Extracellular matrix type modulates mechanotransduction of stem cells. Acta Biomater..

[298] Fu X., Kim H.S. (2024). Dentin mechanobiology: Bridging the gap between architecture and function. Int. J. Mol. Sci..

[299] Li H., Zhang D., Bao P. (2024). Recent advances in functional hydrogels for treating dental hard tissue and endodontic diseases. ACS Nano.

[300] Song Y., Soto J., Chen B. (2020). Cell engineering: Biophysical regulation of the nucleus. Biomaterials.

[301] Guo S., Debbi L., Zohar B. (2021). Stimulating extracellular vesicles production from engineered tissues by mechanical forces. Nano Lett..

[302] Sun X.F., Qiao W.W., Meng L.Y. (2022). Piezo1 ion channels mediate mechanotransduction in odontoblasts. J. Endod..

[303] Xu X., Guo Y., Liu P. (2024). Piezo mediates the mechanosensation and injury-repair of pulpo-dentinal complex. Int. Dent. J..

[304] Matsunaga M., Kimura M., Ouchi T. (2021). Mechanical stimulation-induced calcium signaling by Piezo1 channel activation in human odontoblast reduces dentin mineralization. Front. Physiol..

[305] Fill T.S., Toogood R.W., Major P.W. (2012). Analytically determined mechanical properties of, and models for the periodontal ligament: Critical review of literature. J. Biomech..

[306] Salamati A., Chen J., Herring S.W. (2020). Functional tooth mobility in young pigs. J. Biomech..

[307] Fill T.S., Carey J.P., Toogood R.W. (2011). Experimentally determined mechanical properties of, and models for, the periodontal ligament: Critical review of current literature. J. Dent. Biomech..

[308] Komatsu K. (2010). Mechanical strength and viscoelastic response of the periodontal ligament in relation to structure. J. Dent. Biomech..

[309] Jin S.S., He D.Q., Wang Y. (2020). Mechanical force modulates periodontal ligament stem cell characteristics during bone remodelling via TRPV4. Cell Prolif..

[310] Schröder A., Neher K., Krenmayr B. (2023). Impact of Piezo1-channel on inflammation and osteoclastogenesis mediated via periodontal ligament fibroblasts during mechanical loading. Eur. J. Oral Sci..

[311] Bae H.J., Shin S.J., Jo S.B. (2024). Cyclic stretch induced epigenetic activation of periodontal ligament cells. Mater. Today Bio.

[312] Zhang J., Dong S., Sivak W.N. (2018). Stem cells in cartilage diseases and repair 2018. Stem Cell. Int..

[313] Ding R., Chen C., Wang L. (2025). Matrix stiffness regulates the osteogenic differentiation of hPDLSCs via DNA methylation. Int. Dent. J..

[314] Brockhaus J., Craveiro R.B., Azraq I. (2021). In vitro compression model for orthodontic tooth movement modulates human periodontal ligament fibroblast proliferation, apoptosis and cell cycle. Biomolecules.

[315] Kaya S., Çifter M., Çekici A. (2020). Effects of orthodontic force magnitude on cell apoptosis and rankl-induced osteoclastogenesis studies in a rat model. J. Orofac. Orthop..

[316] Wen X., Pei F., Jin Y. (2025). Exploring the mechanical and biological interplay in the periodontal ligament. Int. J. Oral Sci..

[317] Zhao Y., Zhang S., Cheng B. (2024). Mechanochemical coupling of MGF mediates periodontal regeneration. Bioeng. Transl. Med..

[318] Feng F., Tu T., Wang H. (2024). Mechano-growth factor regulates periodontal ligament stem cell proliferation and differentiation through Fyn-RhoA-YAP signaling. Biochem. Biophys. Res. Commun..

[319] Lettry S., Seedhom B.B., Berry E. (2003). Quality assessment of the cortical bone of the human mandible. Bone.

[320] Misch C.E., Qu Z., Bidez M.W. (1999). Mechanical properties of trabecular bone in the human mandible: Implications for dental implant treatment planning and surgical placement. J. Oral Maxillofac. Surg..

[321] Lin W., Li Q., Zhang D. (2021). Mapping the immune microenvironment for mandibular alveolar bone homeostasis at single-cell resolution. Bone Res..

[322] Rücklin M., Donoghue P.C.J., Johanson Z. (2012). Development of teeth and jaws in the earliest jawed vertebrates. Nature.

[323] Uchida T., Shimizu S., Yamagishi R. (2021). Mechanical stretch induces Ca^2+^ influx and extracellular release of PGE_2_ through Piezo1 activation in trabecular meshwork cells. Sci. Rep..

[324] Ni D. (2024). The hippo pathway in oral diseases and treatments: A review. Medicine (Baltim.).

[325] Mao C.Y., Wang Y.G., Zhang X. (2016). Double-edged-sword effect of IL-1β on the osteogenesis of periodontal ligament stem cells via crosstalk between the NF-κB, MAPK and BMP/Smad signaling pathways. Cell Death Dis..

[326] Sun X.D., Liu T.T., Wang Q.Q. (2023). Surface modification and functionalities for titanium dental implants. ACS Biomater. Sci. Eng..

[327] Hosseini-Faradonbeh S.A., Katoozian H.R. (2022). Biomechanical evaluations of the long-term stability of dental implant using finite element modeling method: A systematic review. J. Adv. Prosthodont..

[328] Ingawalé S., Goswami T. (2009). Temporomandibular joint: Disorders, treatments, and biomechanics. Ann. Biomed. Eng..

[329] Mosaddad S.A., Hussain A., Tebyaniyan H. (2024). Exploring the use of animal models in craniofacial regenerative medicine: A narrative review. Tissue Eng., Part B.

[330] Ananthan S., Pertes R.A., Bender S.D. (2023). Biomechanics and derangements of the temporomandibular joint. Dent. Clin..

[331] Singh M., Detamore M.S. (2009). Biomechanical properties of the mandibular condylar cartilage and their relevance to the tmj disc. J. Biomech..

[332] Liu L., Chen L., Mai Z. (2016). Cyclical compressive stress induces differentiation of rat primary mandibular condylar chondrocytes through phosphorylated myosin light chain II. Mol. Med. Rep..

[333] Chen G., Zhao H., Ma S. (2020). Circadian rhythm protein Bmal1 modulates cartilage gene expression in temporomandibular joint osteoarthritis via the MAPK/ERK pathway. Front. Pharmacol..

[334] Xiao D., Wang R., Hu J. (2017). Spatial and temporal expression of smad signaling members during the development of mandibular condylar cartilage. Exp. Ther. Med..

[335] Le Révérend B.J.D., Edelson L.R., Loret C. (2014). Anatomical, functional, physiological and behavioural aspects of the development of mastication in early childhood. Br. J. Nutr..

[336] Nelson C.M., Xiao B., Wickström S.A. (2024). Mechanobiology: Shaping the future of cellular form and function. Cell.

[337] Feng Y., Liao Z., Zhang H. (2023). Emerging nanomedicines strategies focused on tumor microenvironment against cancer recurrence and metastasis. Chem. Eng. J..

[338] Zhou H., Wang M., Zhang Y. (2022). Functions and clinical significance of mechanical tumor microenvironment: Cancer cell sensing, mechanobiology and metastasis. Cancer Commun..

[339] Liang L., Song X., Zhao H. (2024). Insights into the mechanobiology of cancer metastasis via microfluidic technologies. APL Bioeng..

[340] Xin Y., Li K., Huang M. (2023). Biophysics in tumor growth and progression: From single mechano-sensitive molecules to mechanomedicine. Oncogene.

[341] Sleeboom J.J.F., van Tienderen G.S., Schenke-Layland K. (2024). The extracellular matrix as hallmark of cancer and metastasis: From biomechanics to therapeutic targets. Sci. Transl. Med..

[342] Tang M., Qu Y., He P. (2024). Heat-inducible CAR-T overcomes adverse mechanical tumor microenvironment in a 3D bioprinted glioblastoma model. Mater. Today Bio.

[343] Chaudhuri P.K., Low B.C., Lim C.T. (2018). Mechanobiology of tumor growth. Chem. Rev..

[344] Tarchi S.M., Pernia Marin M., Hossain M.M. (2023). Breast stiffness, a risk factor for cancer and the role of radiology for diagnosis. J. Transl. Med..

[345] Panciera T., Citron A., Di Biagio D. (2020). Reprogramming normal cells into tumour precursors requires ECM stiffness and oncogene-mediated changes of cell mechanical properties. Nat. Mater..

[346] Jang M., An J., Oh S.W. (2021). Matrix stiffness epigenetically regulates the oncogenic activation of the yes-associated protein in gastric cancer. Nat. Biomed. Eng..

[347] Lampi M.C., Reinhart-King C.A. (2018). Targeting extracellular matrix stiffness to attenuate disease: From molecular mechanisms to clinical trials. Sci. Transl. Med..

[348] Pothapragada S.P., Gupta P., Mukherjee S. (2022). Matrix mechanics regulates epithelial defence against cancer by tuning dynamic localization of filamin. Nat. Commun..

[349] Bansaccal N., Vieugue P., Sarate R. (2023). The extracellular matrix dictates regional competence for tumour initiation. Nature.

[350] Zhang S., Xiao X., Yi Y. (2024). Tumor initiation and early tumorigenesis: Molecular mechanisms and interventional targets. Signal Transduct. Targeted Ther..

[351] Almagro J., Messal H.A., Elosegui-Artola A. (2022). Tissue architecture in tumor initiation and progression. Trends Cancer.

[352] Fiore V.F., Krajnc M., Quiroz F.G. (2020). Mechanics of a multilayer epithelium instruct tumour architecture and function. Nature.

[353] Messal H.A., Alt S., Ferreira R.M.M. (2019). Tissue curvature and apicobasal mechanical tension imbalance instruct cancer morphogenesis. Nature.

[354] Nyga A., Muñoz J.J., Dercksen S. (2021). Oncogenic ras instructs morphological transformation of human epithelia via differential tissue mechanics. Sci. Adv..

[355] Lee J., Abdeen A.A., Wycislo K.L. (2016). Interfacial geometry dictates cancer cell tumorigenicity. Nat. Mater..

[356] Chen X., Tang K., Li X. (2022). Biomechanics of cancer stem cells. Essays Biochem..

[357] Tavares S., Vieira A.F., Taubenberger A.V. (2017). Actin stress fiber organization promotes cell stiffening and proliferation of pre-invasive breast cancer cells. Nat. Commun..

[358] Faria L., Canato S., Jesus T.T. (2023). Activation of an actin signaling pathway in pre-malignant mammary epithelial cells by P-cadherin is essential for transformation. Dis. Model. Mech..

[359] Boyle S.T., Poltavets V., Kular J. (2020). Rock-mediated selective activation of PERK signalling causes fibroblast reprogramming and tumour progression through a CRELD2-dependent mechanism. Nat. Cell Biol..

[360] Lee S.W., Morishita Y. (2017). Possible roles of mechanical cell elimination intrinsic to growing tissues from the perspective of tissue growth efficiency and homeostasis. PLoS Comput. Biol..

[361] Jiang K., Lim S.B., Xiao J. (2023). Deleterious mechanical deformation selects mechanoresilient cancer cells with enhanced proliferation and chemoresistance. Adv. Sci..

[362] Wang T.-C., Sawhney S., Morgan D. (2024). Genetic variation drives cancer cell adaptation to ECM stiffness. Proc. Natl. Acad. Sci. USA.

[363] Gensbittel V., Kräter M., Harlepp S. (2021). Mechanical adaptability of tumor cells in metastasis. Dev. Cell.

[364] Lee C.K., Jeong S.H., Jang C. (2019). Tumor metastasis to lymph nodes requires YAP-dependent metabolic adaptation. Science.

[365] Medjkane S., Perez-Sanchez C., Gaggioli C. (2009). Myocardin-related transcription factors and SRF are required for cytoskeletal dynamics and experimental metastasis. Nat. Cell Biol..

[366] Watson A.W., Grant A.D., Parker S.S. (2021). Breast tumor stiffness instructs bone metastasis via maintenance of mechanical conditioning. Cell Rep..

[367] Nava M.M., Miroshnikova Y.A., Biggs L.C. (2020). Heterochromatin-driven nuclear softening protects the genome against mechanical stress-induced damage. Cell.

[368] Hirata E., Ishibashi K., Kohsaka S. (2020). The brain microenvironment induces DNMT1 suppression and indolence of metastatic cancer cells. iScience.

[369] Cambria E., Coughlin M.F., Floryan M.A. (2024). Linking cell mechanical memory and cancer metastasis. Nat. Rev. Cancer.

[370] Tang K., Zheng Y., Hu G. (2025). Local soft niches in mechanically heterogeneous primary tumors promote brain metastasis via mechanotransduction-mediated HDAC3 activity. Sci. Adv..

[371] Qu Y., Cui J., Wu Z. (2026). Mechano-regulation of cancer cell memory in tumor progression and therapy. Mechan. Med..

[372] Li L., Hu J., Shi X. (2021). Interplay between cooperativity of intercellular receptor-ligand binding and coalescence of nanoscale lipid clusters in adhering membranes. Soft Matter.

[373] Li L., Hu J., Różycki B. (2020). Intercellular receptor-ligand binding and thermal fluctuations facilitate receptor aggregation in adhering membranes. Nano Lett..

[374] Li L., Hu J., Li L. (2019). Binding constant of membrane-anchored receptors and ligands that induce membrane curvatures. Soft Matter.

[375] Li L., Hu J., Shi X. (2017). Lipid rafts enhance the binding constant of membrane-anchored receptors and ligands. Soft Matter.

[376] Li L., Hu J., Wu H. (2021). Cis-interaction of ligands on a supported lipid bilayer affects their binding to cell adhesion receptors. Sci. China Phys. Mech. Astron..

[377] Li L., Hu J., Xu G. (2018). Binding constant of cell adhesion receptors and substrate-immobilized ligands depends on the distribution of ligands. Phys. Rev. E.

[378] Jeffreys N., Brockman J.M., Zhai Y. (2024). Mechanical forces amplify TCR mechanotransduction in T cell activation and function. Appl. Phys. Rev..

[379] Rogers J., Bajur A.T., Salaita K. (2024). Mechanical control of antigen detection and discrimination by T and B cell receptors. Biophys. J..

[380] Rushdi M., Li K., Yuan Z. (2020). Mechanotransduction in T cell development, differentiation and function. Cells.

[381] Pageon S.V., Govendir M.A., Kempe D. (2018). Mechanoimmunology: Molecular-scale forces govern immune cell functions. Mol. Biol. Cell.

[382] Chakraborty A.K., Weiss A. (2014). Insights into the initiation of TCR signaling. Nat. Immunol..

[383] van der Merwe P.A., Dushek O. (2011). Mechanisms for T cell receptor triggering. Nat. Rev. Immunol..

[384] Li L., Xu G.K., Song F. (2017). Impact of lipid rafts on the T-cell-receptor and peptide-major-histocompatibility-complex interactions under different measurement conditions. Phys. Rev. E.

[385] Li L., Wang X., Wu H. (2021). Interplay between receptor-ligand binding and lipid domain formation depends on the mobility of ligands in cell-substrate adhesion. Front. Mol. Biosci..

[386] Huang J., Brameshuber M., Zeng X. (2013). A single peptide-major histocompatibility complex ligand triggers digital cytokine secretion in CD4^+^ T cells. Immunity.

[387] Gascoigne N.R., Zal T., Alam S.M. (2001). T-cell receptor binding kinetics in T-cell development and activation. Expet Rev. Mol. Med..

[388] Liu B., Chen W., Evavold B.D. (2014). Accumulation of dynamic catch bonds between TCR and agonist peptide-MHC triggers T cell signaling. Cell.

[389] Sibener L.V., Fernandes R.A., Kolawole E.M. (2018). Isolation of a structural mechanism for uncoupling t cell receptor signaling from peptide-MHC binding. Cell.

[390] Zhao X., Kolawole E.M., Chan W. (2022). Tuning T cell receptor sensitivity through catch bond engineering. Science.

[391] Hong J., Persaud S.P., Horvath S. (2015). Force-regulated in situ TCR-peptide-bound MHC class II kinetics determine functions of CD4^+^ T cells. J. Immunol..

[392] Liu B., Kolawole E.M., Evavold B.D. (2021). Mechanobiology of t cell activation: To catch a bond. Annu. Rev. Cell Dev. Biol..

[393] Choi H.K., Zhu C. (2025). Catch bonds in immunology. Annu. Rev. Immunol..

[394] Kim S.T., Takeuchi K., Sun Z.Y.J. (2009). The αβ T cell receptor is an anisotropic mechanosensor. J. Biol. Chem..

[395] Grakoui A., Bromley S.K., Sumen C. (1999). The immunological synapse: A molecular machine controlling T cell activation. Science.

[396] Chaplin D.D. (2010). Overview of the immune response. J. Allergy Clin. Immunol..

[397] Li L., Hu J., Różycki B. (2021). Influence of lipid rafts on pattern formation during T-cell adhesion. New J. Phys..

[398] Hong J., Ge C., Jothikumar P. (2018). A TCR mechanotransduction signaling loop induces negative selection in the thymus. Nat. Immunol..

[399] Natkanski E., Lee W.Y., Mistry B. (2013). B cells use mechanical energy to discriminate antigen affinities. Science.

[400] Spillane K.M., Tolar P. (2018). Mechanics of antigen extraction in the B cell synapse. Mol. Immunol..

[401] Wang J.C., Yim Y.I., Wu X. (2022). A B-cell actomyosin arc network couples integrin co-stimulation to mechanical force-dependent immune synapse formation. eLife.

[402] Zeng Y., Yi J., Wan Z. (2015). Substrate stiffness regulates B-cell activation, proliferation, class switch, and T-cell-independent antibody responses in vivo. Eur. J. Immunol..

[403] Zhu C., Chen W., Lou J. (2019). Mechanosensing through immunoreceptors. Nat. Immunol..

[404] Bell G.I. (1978). Models for specific adhesion of cells to cells. Science.

[405] Dembo M., Torney D.C., Saxman K. (1988). The reaction-limited kinetics of membrane-to-surface adhesion and detachment. P. Roy. Soc. B-Biol. Sci..

[406] Marshall B.T., Long M., Piper J.W. (2003). Direct observation of catch bonds involving cell-adhesion molecules. Nature.

[407] Wu P., Zhang T., Liu B. (2019). Mechano-regulation of peptide-MHC Class I conformations determines TCR antigen recognition. Mol. Cell.

[408] Fan J., Shi J., Zhang Y. (2022). NKG2D discriminates diverse ligands through selectively mechano-regulated ligand conformational changes. EMBO J..

[409] Qin R., Zhang Y., Shi J. (2025). TCR catch bonds nonlinearly control CD8 cooperation to shape T cell specificity. Cell Res..

[410] Wan Z., Chen X., Chen H. (2015). The activation of IgM- or isotype-switched IgG- and IgE-BCR exhibits distinct mechanical force sensitivity and threshold. eLife.

[411] Wang N., Tytell J.D., Ingber D.E. (2009). Mechanotransduction at a distance: Mechanically coupling the extracellular matrix with the nucleus. Nat. Rev. Mol. Cell Biol..

[412] Dong D., Zheng L., Lin J. (2019). Structural basis of assembly of the human T cell receptor-CD3 complex. Nature.

[413] Chen Y., Zhu Y., Li X. (2022). Cholesterol inhibits TCR signaling by directly restricting TCR-CD3 core tunnel motility. Mol. Cell.

[414] Susac L., Vuong M.T., Thomas C. (2022). Structure of a fully assembled tumor-specific T cell receptor ligated by pMHC. Cell.

[415] Saotome K., Dudgeon D., Colotti K. (2023). Structural analysis of cancer-relevant TCR-CD3 and peptide-MHC complexes by cryoem. Nat. Commun..

[416] Su Q., Chen M., Shi Y. (2022). Cryo-EM structure of the human IgM B cell receptor. Science.

[417] Ma X., Zhu Y., Dong D. (2022). Cryo-EM structures of two human B cell receptor isotypes. Science.

[418] Dong Y., Pi X., Bartels-Burgahn F. (2022). Structural principles of B cell antigen receptor assembly. Nature.

[419] Chen H., Xu X., Hu W. (2023). Self- programmed dynamics of T cell receptor condensation. Proc. Natl. Acad. Sci. USA.

[420] Su X., Ditlev J.A., Hui E. (2016). Phase separation of signaling molecules promotes T cell receptor signal transduction. Science.

[421] Liu L., Yoon C.W., Yuan Z. (2023). Cellular and molecular imaging of CAR-T cell-based immunotherapy. Adv. Drug Deliv. Rev..

[422] Xiao Q., McAtee C.K., Su X. (2022). Phase separation in immune signalling. Nat. Rev. Immunol..

[423] Smith-Garvin J.E., Koretzky G.A., Jordan M.S. (2009). T cell activation. Annu. Rev. Immunol..

[424] Wen Y., Jing Y., Yang L. (2019). The regulators of BCR signaling during B cell activation. Blood Sci..

[425] Bhanja A., Rey-Suarez I., Song W. (2022). Bidirectional feedback between BCR signaling and actin cytoskeletal dynamics. FASEB J..

[426] Nowosad C.R., Spillane K.M., Tolar P. (2016). Germinal center B cells recognize antigen through a specialized immune synapse architecture. Nat. Immunol..

[427] Fardian-Melamed N., Skripka A., Ursprung B. (2025). Infrared nanosensors of piconewton to micronewton forces. Nature.

[428] Casar J.R., McLellan C.A., Shi C. (2025). Upconverting microgauges reveal intraluminal force dynamics in vivo. Nature.

[429] Du H., Bartleson J.M., Butenko S. (2023). Tuning immunity through tissue mechanotransduction. Nat. Rev. Immunol..

[430] Bilal A., Constantin F., Chirila S. (2025). New trends in the treatment of open-angle glaucoma: A critical review. Int. Ophthalmol..

[431] Di X., Gao X., Peng L. (2023). Cellular mechanotransduction in health and diseases: From molecular mechanism to therapeutic targets. Signal Transduct. Targeted Ther..

[432] Walker H.K., Hall W.D., Hurst J.W. (1990).

[433] Yernault J.C., Bohadana A.B. (1995). Chest percussion. Eur. Respir. J..

[434] Wuerfel J., Paul F., Beierbach B. (2010). MR-elastography reveals degradation of tissue integrity in multiple sclerosis. Neuroimage.

[435] Huwart L., Sempoux C., Vicaut E. (2008). Magnetic resonance elastography for the noninvasive staging of liver fibrosis. Gastroenterology.

[436] Bunevicius A., Schregel K., Sinkus R. (2020). Review: MR elastography of brain tumors. Neuroimage, Clin..

[437] Akkaya H.E., Erden A., Kuru Öz D. (2018). Magnetic resonance elastography: Basic principles, technique, and clinical applications in the liver. Diagn. Interv. Radiol..

[438] Nathanson S.D., Nelson L. (1994). Interstitial fluid pressure in breast-cancer, benign breast conditions, and breast parenchyma. Ann. Surg Oncol..

[439] Pang J.C., Aquino K.M., Oldehinkel M. (2023). Geometric constraints on human brain function. Nature.

[440] Jia M., Li Q., Guo J. (2022). Deletion of BACH1 attenuates atherosclerosis by reducing endothelial inflammation. Circ. Res..

[441] Zhang Z., Li G.Y., Jiang Y. (2023). Noninvasive measurement of local stress inside soft materials with programmed shear waves. Sci. Adv..

[442] Kerensky M.J., Paul A., Routkevitch D. (2024). Tethered spinal cord tension assessed via ultrasound elastography in computational and intraoperative human studies. Commun. Med..

[443] Hiscox L.V., Johnson C.L., Barnhill E. (2016). Magnetic resonance elastography (MRE) of the human brain: Technique, findings and clinical applications. Phys. Med. Biol..

[444] Liu H.C., Zeng Y., Gong C. (2024). Wearable bioadhesive ultrasound shear wave elastography. Sci. Adv..

[445] Davidov Y., Shem-Tov N., Yerushalmi R. (2024). Liver stiffness measurements predict sinusoidal obstructive syndrome after hematopoietic stem cell transplantation. Bone Marrow Transplant..

[446] Chang P.E., Hartono J.L., Ngai Y.L. (2019). Optimal liver stiffness measurement values for the diagnosis of significant fibrosis and cirrhosis in chronic liver disease in singapore. Singap. Med. J..

[447] Balleyguier C., Lakhdar A.B., Dunant A. (2018). Value of whole breast magnetic resonance elastography added to MRI for lesion characterization. NMR Biomed..

[448] Lin X., Chang C., Wu C. (2018). Confirmed value of shear wave elastography for ultrasound characterization of breast masses using a conservative approach in chinese women: A large-size prospective multicenter trial. Cancer Manag. Res..

[449] Fedosov D.A., Gompper G. (2024). Cells on a string: Characterizing cellular structure and dynamics through viscoelastic phenotyping. Biophys. J..

[450] Xu W., Mezencev R., Kim B. (2012). Cell stiffness is a biomarker of the metastatic potential of ovarian cancer cells. PLoS One.

[451] Kraning-Rush C.M., Califano J.P., Reinhart-King C.A. (2012). Cellular traction stresses increase with increasing metastatic potential. PLoS One.

[452] Ali D.S., Sofela S.O., Deliorman M. (2024). OMEF biochip for evaluating red blood cell deformability using dielectrophoresis as a diagnostic tool for type 2 diabetes mellitus. Lab Chip.

[453] You J., Park C.A., Kim A.K. (2025). Ultrasensitive microfluidic detection of red blood cell deformability: Age-related decline in deformability. Phys. Fluids.

[454] Urbanska M., Muñoz H.E., Shaw Bagnall J. (2020). A comparison of microfluidic methods for high-throughput cell deformability measurements. Nat. Methods.

[455] Chen Y., Guo K., Jiang L. (2023). Microfluidic deformability cytometry: A review. Talanta.

[456] Islam M., Mezencev R., McFarland B. (2018). Microfluidic cell sorting by stiffness to examine heterogenic responses of cancer cells to chemotherapy. Cell Death Dis..

[457] Islam M., Raj A., McFarland B. (2020). Stiffness based enrichment of leukemia cells using microfluidics. APL Bioeng..

[458] Hu W., Wu C., Long J. (2024). Mechano-immunological checkpoints: An emerging strategy for investigation and evaluation of disease and therapeutics. Smart Mater. Med..

[459] Desgrosellier J.S., Cheresh D.A. (2010). Integrins in cancer: Biological implications and therapeutic opportunities. Nat. Rev. Cancer.

[460] Sun W., Chi S., Li Y. (2019). The mechanosensitive Piezo1 channel is required for bone formation. eLife.

[461] Pankova D., Jiang Y., Chatzifrangkeskou M. (2019). RASSF1A controls tissue stiffness and cancer stem-like cells in lung adenocarcinoma. EMBO J..

[462] Hu Y., Li H., Zhang C. (2024). DNA-based forcechrono probes for deciphering single-molecule force dynamics in living cells. Cell.

[463] Baig M.M.F.A., Lai W.-F., Akhtar M.F. (2020). DNA nanotechnology as a tool to develop molecular tension probes for bio-sensing and bio-imaging applications: An up-to-date review. Nano-Struct. Nano-Objects.

[464] Ge M., Zou H., Chen J. (2024). Cellular fibronectin-targeted fluorescent aptamer probes for early detection and staging of liver fibrosis. Acta Biomater..

[465] Kalli M., Poskus M.D., Stylianopoulos T. (2023). Beyond matrix stiffness: Targeting force-induced cancer drug resistance. Trends Cancer.

[466] Xie N., Tian J., Li Z. (2024). Invited review for 20th anniversary special issue of plrev "AI for mechanomedicine". Phys. Life Rev..

[467] Rozen R., Weihs D. (2021). Machine-learning provides patient-specific prediction of metastatic risk based on innovative, mechanobiology assay. Ann. Biomed. Eng..

[468] Reinherz E.L., Hwang W., Lang M.J. (2023). Harnessing αβ T cell receptor mechanobiology to achieve the promise of immuno-oncology. Proc. Natl. Acad. Sci. USA.

[469] Reinherz E.L. (2015). Αβ TCR-mediated recognition: Relevance to tumor-antigen discovery and cancer immunotherapy. Cancer Immunol. Res..

[470] Zhao L., Zhao G., Feng J. (2023). T cell engineering for cancer immunotherapy by manipulating mechanosensitive force-bearing receptors. Front. Bioeng. Biotechnol..

[471] Lei K., Kurum A., Tang L. (2020). Mechanical immunoengineering of T cells for therapeutic applications. Acc. Chem. Res..

[472] Madsen C.D., Pedersen J.T., Venning F.A. (2015). Hypoxia and loss of PHD2 inactivate stromal fibroblasts to decrease tumour stiffness andmetastasis. EMBO Rep..

[473] Zhang D., Wang G., Yu X. (2022). Enhancing CRISPR/Cas gene editing through modulating cellular mechanical properties for cancer therapy. Nat. Nanotechnol..

[474] Chan N., Willis A., Kornhauser N. (2017). Influencing the tumor microenvironment: A phase II study of copper depletion using tetrathiomolybdate in patients with breast cancer at high risk for recurrence and in preclinical models of lung metastases. Clin. Cancer Res..

[475] Franklin J.M., Wu Z., Guan K.L. (2023). Insights into recent findings and clinical application of YAP and TAZ in cancer. Nat. Rev. Cancer.

[476] Linke J.A., Munn L.L., Jain R.K. (2024). Compressive stresses in cancer: Characterization and implications for tumour progression and treatment. Nat. Rev. Cancer.

[477] Zanconato F., Cordenonsi M., Piccolo S. (2016). YAP/TAZ at the roots of cancer. Cancer Cell.

[478] Gerber D.E., Camidge D.R., Morgensztern D. (2020). Phase 2 study of the focal adhesion kinase inhibitor defactinib (VS-6063) in previously treated advanced KRAS mutant non-small cell lung cancer. Lung Cancer.

[479] Yap T.A., Kwiatkowski D.J., Dagogo-Jack I. (2025). YAP/tead inhibitor VT3989 in solid tumors: A phase 1/2 trial. Nat. Med..

[480] Bryan D.S., Stack M., Krysztofiak K. (2020). 4-hydroxyacetophenone modulates the actomyosin cytoskeleton to reduce metastasis. Proc. Natl. Acad. Sci. USA.

[481] Chen X., Fan Y., Sun J. (2021). Nanoparticle-mediated specific elimination of soft cancer stem cells by targeting low cell stiffness. Acta Biomater..

[482] Du P., Tang K., Chen X. (2025). Intercellular contractile force attenuates chemosensitivity through Notch- MVP- mediated nuclear drug export. Proc. Natl. Acad. Sci. USA.

[483] Zhang P., Meng J., Li Y. (2021). Nanotechnology-enhanced immunotherapy for metastatic cancer. Innovation.

[484] Kong Y., Yu J., Ge S. (2023). Novel insight into RNA modifications in tumor immunity: Promising targets to prevent tumor immune escape. Innovation.

[485] Zhang J., Li J., Hou Y. (2024). Osr2 functions as a biomechanical checkpoint to aggravate CD8^+^ T cell exhaustion in tumor. Cell.

[486] Blumenthal D., Chandra V., Avery L. (2020). Mouse T cell priming is enhanced by maturation-dependent stiffening of the dendritic cell cortex. eLife.

[487] Salmon H., Franciszkiewicz K., Damotte D. (2012). Matrix architecture defines the preferential localization and migration of T cells into the stroma of human lung tumors. J. Clin. Investig..

[488] Basu R., Whitlock B.M., Husson J. (2016). Cytotoxic T cells use mechanical force to potentiate target cell killing. Cell.

[489] Huse M. (2017). Mechanical forces in the immune system. Nat. Rev. Immunol..

[490] Liu Y., Zhang T., Zhang H. (2021). Cell softness prevents cytolytic T-cell killing of tumor-repopulating cells. Cancer Res..

[491] Tello-Lafoz M., Srpan K., Sanchez E.E. (2021). Cytotoxic lymphocytes target characteristic biophysical vulnerabilities in cancer. Immunity.

[492] Zhou Y., Wang D., Zhou L. (2024). Cell softness renders cytotoxic T lymphocytes and T leukemic cells resistant to perforin-mediated killing. Nat. Commun..

[493] Lei K., Kurum A., Kaynak M. (2021). Cancer-cell stiffening via cholesterol depletion enhances adoptive T-cell immunotherapy. Nat. Biomed. Eng..

[494] Li R., Ma C., Cai H. (2020). The car T-cell mechanoimmunology at a glance. Adv. Sci..

[495] Zhang Z., Gao Q., Ren X. (2023). Characterization of intratumor microbiome in cancer immunotherapy. Innovation.

[496] Lu Z., Peng Z., Liu C. (2020). Current status and future perspective of immunotherapy in gastrointestinal cancers. Innovation.

[497] Salter A.I., Rajan A., Kennedy J.J. (2021). Comparative analysis of TCR and CAR signaling informs CAR designs with superior antigen sensitivity and in vivo function. Sci. Signal..

[498] Gudipati V., Rydzek J., Doel-Perez I. (2020). Inefficient CAR-proximal signaling blunts antigen sensitivity. Nat. Immunol..

[499] Han H.M., Kim S.Y., Kim D.H. (2025). Mechanotransduction for therapeutic approaches: Cellular aging and rejuvenation. APL Bioeng..

[500] Kalukula Y., Ciccone G., Mohammed D. (2025). Unlocking the therapeutic potential of cellular mechanobiology. Sci. Adv..

[501] Lee J., Henderson K., Massidda M.W. (2021). Mechanobiological conditioning of mesenchymal stem cells for enhanced vascular regeneration. Nat. Biomed. Eng..

[502] Ichijo R., Maki K., Kabata M. (2022). Vasculature atrophy causes a stiffened microenvironment that augments epidermal stem cell differentiation in aged skin. Nat. Aging.

[503] Zhang X., Cao D., Xu L. (2023). Harnessing matrix stiffness to engineer a bone marrow niche for hematopoietic stem cell rejuvenation. Cell Stem Cell.

[504] Gupta R., Alkhalfan F., Wheeler J. (2025). Biomechanical platelet activation: Diseases that require a new class of antiplatelet therapeutics. Am. J. Physiol. Cell Physiol..

[505] Muñoz-Espín D., Serrano M. (2014). Cellular senescence: From physiology to pathology. Nat. Rev. Mol. Cell Biol..

[506] Rossiello F., Jurk D., Passos J.F. (2022). Telomere dysfunction in ageing and age-related diseases. Nat. Cell Biol..

[507] Chang L., Du H., Xu F. (2024). Hydrogel-enabled mechanically active wound dressings. Trends Biotechnol..

[508] Chang L., Li Y., Li M. (2021). An injectable, biodegradable magnetic hydrogel system for exogenous promotion of muscle mass and regeneration. Chem. Eng. J..

[509] Wallace G.Q., McNally E.M. (2009). Mechanisms of muscle degeneration, regeneration, and repair in the muscular dystrophies. Annu. Rev. Physiol..

[510] Saha S., Panigrahi D.P., Patil S. (2018). Autophagy in health and disease: A comprehensive review. Biomed. Pharmacother..

[511] Sicari B.M., Rubin J.P., Dearth C.L. (2014). An acellular biologic scaffold promotes skeletal muscle formation in mice and humans with volumetric muscle loss. Sci. Transl. Med..

[512] Sayegh S.A. (2021).

[513] Cole H.A., Carlson C.R. (2018). Mind-body considerations in orofacial pain. Dent. Clin..

[514] Ma Y., Lin M., Huang G. (2018). 3D spatiotemporal mechanical microenvironment: A hydrogel-based platform for guiding stem cell fate. Adv. Mater..

[515] Madl C.M., Flaig I.A., Holbrook C.A. (2021). Biophysical matrix cues from the regenerating niche direct muscle stem cell fate in engineered microenvironments. Biomaterials.

[516] Rao N., Agmon G., Tierney M.T. (2017). Engineering an injectable muscle-specific microenvironment for improved cell delivery using a nanofibrous extracellular matrix hydrogel. ACS Nano.

[517] Shi N., Wang J., Tang S. (2024). Matrix nonlinear viscoelasticity regulates skeletal myogenesis through MRTF nuclear localization and nuclear mechanotransduction. Small.

[518] Bian W., Liau B., Badie N. (2009). Mesoscopic hydrogel molding to control the 3D geometry of bioartificial muscle tissues. Nat. Protoc..

[519] Shi N., Li Y., Chang L. (2021). A 3D, magnetically actuated, aligned collagen fiber hydrogel platform recapitulates physical microenvironment of myoblasts for enhancing myogenesis. Small Methods.

[520] Fang Y., Yang X., Lin Y. (2022). Dissecting biological and synthetic soft-hard interfaces for tissue-like systems. Chem. Rev..

[521] Zhang H., Ma Y., Wang Y. (2023). Rational design of soft-hard interfaces through bioinspired engineering. Small.

[522] Saldivar M.C., Tay E., Isaakidou A. (2023). Bioinspired rational design of bi-material 3D printed soft-hard interfaces. Nat. Commun..

[523] Zhang H., Ma Y., Shu W. (2024). Cellular-scale matrix stiffness gradient at soft-hard tissue interfaces regulates immunophenotype of mesenchymal stem cells. Adv. Funct. Mater..

[524] Lin J., He Y., He Y. (2023). Janus functional electrospun polyurethane fibrous membranes for periodontal tissue regeneration. J. Mater. Chem. B.

[525] Liu X., Wan X., Sui B. (2024). Piezoelectric hydrogel for treatment of periodontitis through bioenergetic activation. Bioact. Mater..

[526] Roldan L., Montoya C., Solanki V. (2023). A novel injectable piezoelectric hydrogel for periodontal disease treatment. ACS Appl. Mater. Interfaces.

[527] Gu Y., Bai Y., Xie X. (2021). Bite force transducers and measurement devices. Front. Bioeng. Biotechnol..

[528] Cheng Z., Li P., Teng J. (2023). A dual-layer orthogonal-layout thin-film force sensor for digital orthodontic functional appliances. IEEE Sens. J..

[529] Zhang H., Wu S., Chen W. (2023). Bone/cartilage targeted hydrogel: Strategies and applications. Bioact. Mater..

[530] Zhang Y., Liu X., Zeng L. (2019). Polymer fiber scaffolds for bone and cartilage tissue engineering. Adv. Funct. Mater..

[531] Wang X., Lin J., Li Z. (2022). Identification of an ultrathin osteochondral interface tissue with specific nanostructure at the human knee joint. Nano Lett..

[532] Cooper R.A., Ohnabe H., Cooper R. (2006).

[533] Smith E.M. (2022). The global report on assistive technology: A new era in assistive technology. Assist. Technol..

[534] Rahman T., Sample W., Seliktar R. (2007). Design and testing of a functional arm orthosis in patients with neuromuscular diseases. IEEE Trans. Neural Syst. Rehabil. Eng..

[535] Stauffer Y., Allemand Y., Bouri M. (2009). The walktrainer-a new generation of walking reeducation device combining orthoses and muscle stimulation. IEEE Trans. Neural Syst. Rehabil. Eng..

[536] Maly M.R., Culham E.G., Costigan P.A. (2002). Static and dynamic biomechanics of foot orthoses in people with medial compartment knee osteoarthritis. Clin. Biomech..

[537] d'Hemecourt P.A., Gerbino P.G., Micheli L.J. (2000). Back injuries in the young athlete. Clin. Sports Med..

[538] Sawicki G.S., Gordon K.E., Ferris D.P. (2005). IEEE 9th Int. Conf. Rehabil. Robot..

[539] Razeghi M., Batt M.E. (2000). Biomechanical analysis of the effect of orthotic shoe inserts - a review of the literature. Sports Med..

[540] du Plessis T., Djouani K., Oosthuizen C. (2021). A review of active hand exoskeletons for rehabilitation and assistance. Robotics.

[541] Tyson S.F., Sadeghi-Demneh E., Nester C.J. (2013). A systematic review and meta-analysis of the effect of an ankle-foot orthosis on gait biomechanics after stroke. Clin. Rehabil..

[542] Shepherd M.K., Rouse E.J. (2017). Design and validation of a torque-controllable knee exoskeleton for sit-to-stand assistance. IEEE ASME Trans. Mechatron..

[543] Zhang Y., Feng X., Zheng B. (2024). Regulation and mechanistic insights into tensile strain in mesenchymal stem cell osteogenic differentiation. Bone.

[544] Hayakawa K., Tatsumi H., Sokabe M. (2011). Actin filaments function as a tension sensor by tension-dependent binding of cofilin to the filament. J. Cell Biol..

[545] Peng Y., Qu R., Feng Y. (2021). Regulation of the integrin αvβ3-actin filaments axis in early osteogenesis of human fibroblasts under cyclic tensile stress. Stem Cell Res. Ther..

[546] Kalita B., Narayan J., Dwivedy S.K. (2021). Development of active lower limb robotic-based orthosis and exoskeleton devices: A systematic review. Int. J. Soc. Robot..

[547] Moltedo M., Baček T., Serrien B. (2020). Walking with a powered ankle-foot orthosis: The effects of actuation timing and stiffness level on healthy users. J. NeuroEng. Rehabil..

[548] Poveda Roda R., Bagan J.V., Díaz Fernández J.M. (2007). Review of temporomandibular joint pathology. Part I: Classification, epidemiology and risk factors. Med. Oral Patol. Oral Cir. Bucal.

[549] Poveda Roda R., Díaz Fernández J.M., Hernández Bazán S. (2008). A review of temporomandibular joint disease (TMJD). Part II: Clinical and radiological semiology. Morbidity processes. Med. Oral Patol. Oral Cir. Bucal.

[550] Zhu Y., Zheng F., Gong Y. (2024). Biomechanical behavior of customized splint for the patient with temporomandibular disorders: A three-dimensional finite element analysis. Biocybern. Biomed. Eng..

[551] Herpich C.M., Amaral A.P., Leal-Junior E.C.P. (2015). Analysis of laser therapy and assessment methods in the rehabilitation of temporomandibular disorder: A systematic review of the literature. J. Phys. Ther. Sci..

[552] Serrano-Muñoz D., Beltran-Alacreu H., Martín-Caro Álvarez D. (2023). Effectiveness of different electrical stimulation modalities for pain and masticatory function in temporomandibular disorders: A systematic review and meta-analysis. J. Pain.

[553] Zheng J., Chen X., Jiang W. (2019). An innovative total temporomandibular joint prosthesis with customized design and 3D printing additive fabrication: A prospective clinical study. J. Transl. Med..

[554] Prasad S., Farella M. (2023). Wearables for personalized monitoring of masticatory muscle activity - opportunities, challenges, and the future. Clin. Oral Invest..

[555] Huiskes R., Chao E.Y. (1983). A survey of finite-element analysis in orthopedic biomechanics - the 1st decade. J. Biomech..

[556] Galbusera F., Bellini C.M., Brayda-Bruno M. (2008). Biomechanical studies on cervical total disc arthroplasty: A literature review. Clin. Biomech..

[557] Lathouwers E., Díaz M.A., Maricot A. (2023). Therapeutic benefits of lower limb prostheses: A systematic review. J. NeuroEng. Rehabil..

[558] Zhang M., Mak A.F., Roberts V.C. (1998). Finite element modelling of a residual lower-limb in a prosthetic socket: A survey of the development in the first decade. Med. Eng. Phys..

[559] Au S.K., Dilworth P., Herr H. (2006). IEEE Int. Conf. Robot. Autom..

[560] Joshi M.G., Advani S.G., Miller F. (2000). Analysis of a femoral hip prosthesis designed to reduce stress shielding. J. Biomech..

[561] Au S.K., Weber J., Herr H. (2007). IEEE 10th Int. Conf. Rehabil. Robot..

[562] Lee J.T., Bartlett H.L., Goldfarb M. (2020). Design of a semipowered stance-control swing-assist transfemoral prosthesis. IEEE ASME Trans. Mechatron..

[563] Peng Y., Wang Y., Wong D.W.C. (2021). Extrinsic foot muscle forces and joint contact forces in flexible flatfoot adult with foot orthosis: A parametric study of tibialis posterior muscle weakness. Gait Posture.

[564] Azocar A.F., Mooney L.M., Duval J.F. (2020). Design and clinical implementation of an open-source bionic leg. Nat. Biomed. Eng..

[565] Gabert L., Hood S., Tran M. (2020). A compact, lightweight robotic ankle-foot prosthesis: Featuring a powered polycentric design. IEEE Robot. Autom. Mag..

[566] Hood S., Gabert L., Lenzi T. (2022). Powered knee and ankle prosthesis with adaptive control enables climbing stairs with different stair heights, cadences, and gait patterns. IEEE Trans. Robot..

[567] van der Woude L.H., Dallmeijer A.J., Janssen T.W. (2001). Alternative modes of manual wheelchair ambulation - an overview. Am. J. Phys. Med. Rehabil..

[568] Collinger J.L., Boninger M.L., Koontz A.M. (2008). Shoulder biomechanics during the push phase of wheelchair propulsion: A multisite study of persons with paraplegia. Arch. Phys. Med. Rehabil..

[569] van der Woude L.H., Veeger H.E., Dallmeijer A.J. (2001). Biomechanics and physiology in active manual wheelchair propulsion. Med. Eng. Phys..

[570] de Klerk R., Vegter R.J.K., Veeger H.E.J. (2020). Technical note: A novel servo-driven dual-roller handrim wheelchair ergometer. IEEE Trans. Neural Syst. Rehabil. Eng..

[571] Chow J.W., Millikan T.A., Carlton L.G. (2009). Kinematic and electromyographic analysis of wheelchair propulsion on ramps of different slopes for young men with paraplegia. Arch. Phys. Med. Rehabil..

[572] Liu Y., Hu W., Kasal A. (2023). The state of the art of biomechanics applied in ergonomic furniture design. Appl. Sci. (Basel).

[573] Krebs H.I., Hogan N., Aisen M.L. (1998). Robot-aided neurorehabilitation. IEEE Trans. Rehabil. Eng..

[574] Sugar T.G., He J., Koeneman E.J. (2007). Design and control of rupert: A device for robotic upper extremity repetitive therapy. IEEE Trans. Neural Syst. Rehabil. Eng..

[575] Young A.J., Ferris D.P. (2017). State of the art and future directions for lower limb robotic exoskeletons. IEEE Trans. Neural Syst. Rehabil. Eng..

[576] Sawers A., Ting L.H. (2014). Perspectives on human-human sensorimotor interactions for the design of rehabilitation robots. J. NeuroEng. Rehabil..

[577] Asbeck A.T., De Rossi S.M.M., Holt K.G. (2015). A biologically inspired soft exosuit for walking assistance. Int. J. Robot Res..

[578] Vallery H., Veneman J., Van Asseldonk E. (2008). Compliant actuation of rehabilitation robots - benefits and limitations of series elastic actuators. IEEE Robot. Autom. Mag..

[579] Shi D., Zhang W., Zhang W. (2019). A review on lower limb rehabilitation exoskeleton robots. Chin. J. Mech. Eng..

[580] Li W., Liu K., Li C. (2022). Development and evaluation of a wearable lower limb rehabilitation robot. J. Bionic Eng..

