# Supplementary material for: Transformative biomechanics and mechanobiology breakthroughs shaping the future of health and medicine
Source: Innovation (Camb). 2026 Feb 6;7(4):101307. doi: 10.1016/j.xinn.2026.101307 (PMC13069430; doi:10.1016/j.xinn.2026.101307)
Supplement: Document S1. Tables S1 and S2 [file mmc1.pdf]

The Innovation, Volume 7

Supplemental Information

Transformative biomechanics and mechanobiology breakthroughs shaping the future of health and medicine

Long Li, Jing Ji, He Ren, Lilan Gao, Xiaona Li, Ning Li, Songbai Zhang, Kai Tang, Zedong Li, Weiyan Ren, Qing-ping Yao, Kai Huang, He Gong, Yingfeng Shao, Xianglong Lin, Xin Wang, Xiuqing Qian, Jie Song, Yiran Jiang, Hui Chen, Bo Che, Dongyuan Lü, Yu Du, Fan Feng, Yanli Liu, Yan Li, Meiying Luo, Ruotian Du, Cunyu Zhang, Guanshuo Hu, Yufei Ma, Shutong Wang, Rui Yang, Fang Pu, Bingjie Xiang, Ming Zhang, Xinghua Shi, Lizhen Wang, Bo Li, Damir Kračun, Qian Chen, Ahmed Elsheikh, Zi-Jun Liu, Baoyu Liu, Chuanrong Zhao, Yonggang Lü, Zhu Zeng, Zhiyong Li, Yiyao Liu, Guixue Wang, Wenchang Tan, Chunqiu Zhang, Min Zhang, Jizhong Lou, Youhua Tan, Linhong Deng, Mian Long, Ying-Xin Qi, Weiyi Chen, Feng Xu, Yubo Fan, and Fan Song

Supplemental Information

Transformative biomechanics and mechanobiology breakthroughs shaping the future of health and medicine

Long Li,[1,2,42] Jing Ji,[3,42] He Ren,[3] Lilan Gao,[4] Xiaona Li,[5] Ning Li,[6] Songbai Zhang,[7] Kai Tang,[8,9] Zedong Li,[10,11] Weiyan Ren,[12] Qing-ping Yao,[13] Kai Huang,[13] He Gong,[3,14] Yingfeng Shao,[1] Xianglong Lin,[4] Xin Wang,[4] Xiuqing Qian[15], Jie Song[5], Yiran Jiang,[16] Hui Chen,[16] Bo Che,[17] Dongyuan Lü,[6] Yu Du,[6] Fan Feng,[7] Yanli Liu,[7] Yan Li,[1] Meiying Luo,[1] Ruotian Du,[1] Cunyu Zhang,[8,9] Guanshuo Hu,[8,9] Yufei Ma,[10,11] Shutong Wang,[10,11] Rui Yang,[10,11] Fang Pu,[3,14] Bingjie Xiang,[3,14] Ming Zhang,[9] Xinghua Shi,[18] Lizhen Wang,[3] Bo Li,[19] Damir Kračun,[20,21] Qian Chen,[22] Ahmed Elsheikh,[23,24] Zi-Jun Liu,[25] Baoyu Liu,[26] Chuanrong Zhao,[27] Yonggang Lü,[28] Zhu Zeng,[29] Zhiyong Li,[30] Yiyao Liu,[31,32] Guixue Wang,[33] Wenchang Tan,[34,35] Chunqiu Zhang,[4,*] Min Zhang,[7,*] Jizhong Lou,[16,36,*] Youhua Tan,[8,9,*] Linhong Deng,[17,*] Mian Long,[6,*] Ying-Xin Qi,[13,*] Weiyi Chen,[5,*] Feng Xu,[10,11,*] Yubo Fan,[3,12,14,*] and Fan Song,[1,37,*]

1. State Key Laboratory of Nonlinear Mechanics and Beijing Key Laboratory of Engineered Construction and Mechanobiology, Institute of Mechanics, Chinese Academy of Sciences, Beijing 100190, China
2. Center of Materials Science and Optoelectronics Engineering, University of Chinese Academy of Sciences, Beijing 100049, China
3. Key Laboratory for Biomechanics and Mechanobiology of Ministry of Education, School of Biological Science and Medical Engineering, Beihang University, Beijing 100083, China
4. Tianjin Key Laboratory for Advanced Mechatronic System Design and Intelligent Control, National Demonstration Center for Experimental Mechanical and Electrical Engineering Education, School of Mechanical Engineering, Tianjin University of Technology, Tianjin 300384, China
5. Institute of Biomedical Engineering, Taiyuan University of Technology, Taiyuan

030024, China
6. Center for Biomechanics and Bioengineering, Beijing Key Laboratory of Engineered Construction and Mechanobiology and Key Laboratory of Microgravity (National Microgravity Laboratory), Institute of Mechanics, Chinese Academy of Sciences, Beijing 100190, China
7. State Key Laboratory of Oral & Maxillofacial Reconstruction and Regeneration, National Clinical Research Center for Oral Diseases, Shaanxi International Joint Research Center for Oral Diseases, Department of General Dentistry and Emergency, School of Stomatology, Fourth Military Medical University, Xi’an 710032, China
8. The Hong Kong Polytechnic University Shenzhen Research Institute, Shenzhen 518057, China
9. Department of Biomedical Engineering, The Hong Kong Polytechnic University, Hong Kong 999077, China
10. The Key Laboratory of Biomedical Information Engineering of Ministry of Education, School of Life Science and Technology, Xi’an Jiaotong University, Xi’an 710049, China
11. Bioinspired Engineering and Biomechanics Center (BEBC), School of Life Science and Technology, Xi’an Jiaotong University, Xi’an 710049, China
12. School of Engineering Medicine, Beihang University, Beijing 100083, China
13. Institute of Mechanobiology & Medical Engineering, School of Life Sciences & Biotechnology, Shanghai Jiao Tong University, Shanghai 200240, China
14. Innovation Center for Medical Engineering & Engineering Medicine, Hangzhou International Innovation Institute, Beihang University, Hangzhou 311115, China
15. School of Biomedical Engineering, Capital Medical University, Beijing 100086, China
16. State Key Laboratory of Epigenetic Regulation and Intervention, CAS Center for Excellence in Biomacromolecules, Institute of Biophysics, Chinese Academy of Sciences, Beijing 100101, China
17. Institute of Biomedical Engineering and Health Sciences, Changzhou University,

Changzhou 213164, China
18. National Center for Nanoscience and Technology, Chinese Academy of Sciences, Beijing 100190, China
19. School of Aerospace Engineering, Tsinghua University, Beijing 100084, China
20. University Clinic Balgrist, Orthopaedic Biomechanics, Forchstrasse 340, 8008, Zurich, Switzerland
21. Institute for Biomechanics, ETH Zurich, Zurich 8092, Switzerland
22. Laboratory of Molecular Biology and Nanomedicine, Department of Orthopaedics, Warren Alpert Medical School of Brown University, Providence RI 02912, USA
23. School of Engineering, University of Liverpool, Liverpool L69 7ZX, UK
24. National Institute for Health Research (NIHR) Biomedical Research Centre, Moorfields Eye Hospital NHS Foundation Trust and UCL Institute of Ophthalmology, London EC1V 2PD, UK
25. Department of Orthodontics, University of Washington, Washington WA 98195-7446, USA
26. Department of Pathology, University of Utah, Salt Lake City UT 84111, USA
27. Bioengineering College of Chongqing University, Chongqing, 400044, China
28. State Key Laboratory of New Textile Materials and Advanced Processing, Wuhan Textile University, Wuhan 430200, China
29. Key Laboratory of Infectious Immune and Antibody Engineering of Guizhou Province, Engineering Research Center of Cellular Immunotherapy of Guizhou Province, Guizhou Medical University, Guiyang 561113, China
30. Faculty of Sports Science, Ningbo University, Ningbo 315211, China
31. Sichuan Provincial Key Laboratory for Human Disease Gene Study, Center for Medical Genetics, Sichuan Provincial People’s Hospital, School of Life Science and Technology, University of Electronic Science and Technology of China, Chengdu, 610054, China
32. TCM Regulating Metabolic Diseases Key Laboratory of Sichuan Province, Hospital of Chengdu University of Traditional Chinese Medicine, Chengdu 610072, China

33. Key Laboratory for Biorheological Science and Technology of Ministry of Education, State and Local Joint Engineering Laboratory for Vascular Implants, Bioengineering College of Chongqing University, Chongqing 400030, China
34. Department of Mechanics and Engineering Science, Peking University, Beijing 100871, China
35. Shenzhen Graduate School, Peking University, Shenzhen 518055, China
36. College of Life Sciences, University of Chinese Academy of Sciences, Beijing 100049, China
37. School of Engineering Sciences, University of Chinese Academy of Sciences, Beijing 100049, China
38. These authors contributed equally

*Correspondence: zcqarticle@163.com (C.Z.); zhangmin@fmmu.edu.cn (M.Z.); jlou@ibp.ac.cn (J.L.); youhua.tan@polyu.edu.hk (Y.T.); dlh@cczu.edu.cn (L.D.); mlong@imech.ac.cn (M.L.); qiyx@sjtu.edu.cn (Y.Q.); chenweiyi@tyut.edu.cn (W.C); fengxu@mail.xjtu.edu.cn (F.X.); yubofan@buaa.edu.cn (Y.F.); songf@lnm.imech.ac.cn (F.S.)

Table S1 Piezo channel-associated mechanotransduction pathway in different systems

Mechanosensor	Biological system	Cell type	Mechanical cues	Mechanotransduction pathway	Target	Reference
Piezo	Cardiovascular system	Vascular endothelial cells	Disturbed flow	Piezo1 → ATP → $P2Y_2$ → Gq/G_{11} → mechanosignaling complex → PI3k → Integrin α5 → FAK → IKK → NFκB	Vascular remodeling	1
		VICs	Oscillatory stress	Piezo1 → Ca^{2+} → YAP → GLS1 → Acetyl-CoA → RUNX2	CAVD	2
		VSMCs	Stiffness	Netrin-1 → α-actinin2 → cytoskeletal stiffness → Piezo1 → Ca^{2+} → MMP3	AAA	3
	Bone and joints	BMSCs	Shear stress	Piezo1/2 → Ca^{2+} → Calcineurin → NFAT/YAP1/β-catenin	Bone formation	4
		Pre-osteoblast	Shear stress	Piezo1 → Ca^{2+} → AKT/GSK-3β/β-catenin → Runx2		5
		Periosteal stem cells	Mechanical stimuli	Piezo1 → Ca^{2+} → YAP → β-catenin		6
	Eye	TM cells	Shear stress	Piezo1 → Ca^{2+} → MMP-2	IOP homeostasis	7
			Stretch	Piezo1 → Ca^{2+}→ cPLA2 → arachidonic acid → PGE2		8
	Liver	Hepatocyte	Membrane tension	Piezo1 → Ca^{2+} → Nrf2 → Nqo1/Gsta1 → ROS level	Acute liver injury	9
		LSECs	Stretch	Notch/Piezo → Hes/Hey → CXCL1	Portal hypertension	10
		Macrophages	ECM Stiffness	Piezo1 → Ca^{2+}/Rac1	Liver fibrosis	11
	Lung	Epithelial cells	Compression	Piezo1/TRP → S1P → $S1P_2$ receptors → Rho	Airway epithelial damage	12
		Fibroblasts	Stretch	Piezo1 → Ca^{2+} → ERK1/2	Pulmonary fibrosis	13
	Craniomandibular system	PDL cell	Orthodontic force	Piezo1/TRPV4 → Ca^{2+} → NFκB/M-CSF/RANKL	Bone remodeling	14
		Dental pulp MSCs	Hydrostatic pressure	Piezo1 → Ca^{2+} → BMP2/Runx2	MSC migration	15
		Macrophages	Mechanical force	Piezo1 → Ca^{2+} → AKT → GSK-3β → Ccnd1	Orthodontic tooth movement	16

Table S2 Integrin-associated mechanotransduction pathway in different systems

Mechanosensor	Biological system	Cell type	Mechanical cues	Mechanotransduction pathway	Target	Reference
Integrin	Cardiovascular system	Vascular endothelial cells	Shear stress	Integrin → Integrin-$G\alpha_{13}$ interaction → RhoA/YAP → JNK signalling/Pro-inflammatory genes	Atherosclerosis	17
		Smooth muscle cells	Stretch	Thbs1 → αvβ1-integrin → GTPase Rap2/Hippo → YAP	Vascular remodeling	18
	Bone and joints	Osteoblasts	Shear stress	β1-integrin → ERK/p38 MAPK → c-fos/Cox-2	Bone formation	19
				Integrin → FAK/Shc → PI3K/Akt/mTOR/MAPK		20
		Chondrocytes	ECM stiffness	α5β1-integrin → FAK/β-catenin/paxillin → IL-4/MMP-3	Osteoarthritis	21
				α5β1-integrin → PKCδ/PYK2 → ERK1/2, JNK1/2, p38 → NFκB/AP-1 → MMP		
	Eye	Scleral fibroblasts	ECM Stiffness	Integrinα1β1 → F-actin → YAP/TAZ	Myopia	22
		Lamina cribrosa cells		Integrinαvβ3 → F-actin → YAP	Glaucoma	23
	Liver	LSECs	Stretch	β1-integrin → F-actin reorganization → BAG-3 → YAP → HB-EGF	Liver regeneration	24
				β1-integrin → VEGFR-3 → HGF/IL-6/TNFα		25
		Hepatocytes	Shear Stress	β1-integrin → FAK → Hippo (p-LATS/LATS) → YAP		26
	Lung	Myofibroblasts	ECM Stiffness	ROCK → c-Fos/c-Jun → α6-integrin → MMP-2	Pulmonary fibrosis	27
	Craniomandibular system	PDL cells	Stretch	Integrin → Rho GTPase → mDia1	Bone remodeling	28
			Compression	Integrin → FAK/Src/Paxillin/Tensin/Filamin		29

REFERENCES

1. Albarrán-Juárez, J., Iring, A., Wang, S.P., et al. (2018). Piezo1 and Gq/G11 promote endothelial inflammation depending on flow pattern and integrin activation. *J. Exp. Med.* **215**:2655-2672. DOI:10.1084/jem.20180483
2. Zhong, G.H., Su, S.W., Li, J.C., et al. (2023). Activation of Piezo1 promotes osteogenic differentiation of aortic valve interstitial cell through YAP-dependent glutaminolysis. *Sci. Adv.* **9**:eadg0478. DOI:10.1126/sciadv.adg0478
3. Qian, W.Y., Hadi, T., Silvestro, M., et al. (2022). Microskeletal stiffness promotes aortic aneurysm by sustaining pathological vascular smooth muscle cell mechanosensation via Piezo1. *Nat. Commun.* **13**:512. DOI:10.1038/s41467-021-27874-5
4. Zhou, T.F., Gao, B., Fan, Y., et al. (2020). Piezo1/2 mediate mechanotransduction essential for bone formation through concerted activation of NFAT-YAP1-β-catenin. *eLife* **9**:e52779. DOI:10.7554/eLife.52779
5. Song, J.D., Liu, L.Y., Lv, L.F., et al. (2020). Fluid shear stress induces Runx-2 expression via upregulation of piezo1 in MC3T3-E1 cells. *Cell Biol. Int.* **44**:1491-1502. DOI:10.1002/cbin.11344
6. Liu, Y.L., Tian, H.T., Hu, Y.X., et al. (2022). Mechanosensitive Piezo1 is crucial for periosteal stem cell-mediated fracture healing. *Int. J. Biol. Sci.* **18**:3961-3980. DOI:10.7150/ijbs.71390
7. Morozumi, W., Aoshima, K., Inagaki, S., et al. (2021). Piezo 1 is involved in intraocular pressure regulation. *J. Pharmacol. Sci.* **147**:211-221. DOI:10.1016/j.jphs.2021.06.005
8. Uchida, T., Shimizu, S., Yamagishi, R., et al. (2021). Mechanical stretch induces Ca^{2+} influx and extracellular release of PGE_2 through Piezo1 activation in trabecular meshwork cells. *Sci. Rep.* **11**:4044. DOI:10.1038/s41598-021-83713-z
9. Wang, Q.M., Peng, X.Y., Chen, Y.F., et al. (2023). Piezo1 alleviates acetaminophen-induced acute liver injury by activating Nrf2 and reducing mitochondrial reactive oxygen species. *Biochem. Biophys. Res. Commun.*

652:88-94. DOI:10.1016/j.bbrc.2023.02.043

10. Hilscher, M.B., Sehrawat, T., Arab, J.P., et al. (2019). Mechanical stretch increases expression of CXCL1 in liver sinusoidal endothelial cells to recruit neutrophils, generate sinusoidal microthombi, and promote portal hypertension. *Gastroenterology* **157**:193-209. DOI:10.1053/j.gastro.2019.03.013

11. Wang, Y., Wang, J., Zhang, J.H., et al. (2024). Stiffness sensing via Piezo1 enhances macrophage efferocytosis and promotes the resolution of liver fibrosis. *Sci. Adv.* **10**:eadj3289. DOI:10.1126/sciadv.adj3289

12. Bagley, D.C., Russell, T., Ortiz-Zapater, E., et al. (2024). Bronchoconstriction damages airway epithelia by crowding-induced excess cell extrusion. *Science* **384**:66-73. DOI:10.1126/science.adk2758

13. Zheng, M.N., Yao, Y., Borkar, N.A., et al. (2024). Piezo channels modulate human lung fibroblast function. *Am. J. Physiol.-Lung Cell. Mol. Physiol.* **327**:L547-L556. DOI:10.1152/ajplung.00356.2023

14. Jin, Y., Li, J., Wang, Y.T., et al. (2015). Functional role of mechanosensitive ion channel Piezo1 in human periodontal ligament cells. *Angle Orthod.* **85**:87-94. DOI:10.2319/123113-955.1

15. Sugimoto, A., Miyazaki, A., Kawarabayashi, K., et al. (2017). Piezo type mechanosensitive ion channel component 1 functions as a regulator of the cell fate determination of mesenchymal stem cells. *Sci. Rep.* **7**:17696. DOI:10.1038/s41598-017-18089-0

16. Xu, H., Guan, J.N., Jin, Z.C., et al. (2022). Mechanical force modulates macrophage proliferation via Piezo1-AKT-Cyclin D1 axis. *FASEB J.* **36**:e22423. DOI:10.1096/fj.202200314R

17. Wang, L., Luo, J.Y., Li, B.C., et al. (2016). Integrin-YAP/TAZ-JNK cascade mediates atheroprotective effect of unidirectional shear flow. *Nature* **540**:579-582. DOI:10.1038/nature20602

18. Yamashiro, Y., Thang, B.Q., Ramirez, K., et al. (2020). Matrix mechanotransduction mediated by thrombospondin-1/integrin/YAP in the vascular remodeling. *Proc. Natl. Acad. Sci. U. S. A.* **117**:9896-9905.

DOI:10.1073/pnas.1919702117

19. Yeh, C.R., Chiu, J.J., Lee, C.I., et al. (2010). Estrogen augments shear stress-induced signaling and gene expression in osteoblast-like cells via estrogen receptor-mediated expression of β1-integrin. *J. Bone Miner. Res.* **25**:627-639. DOI:10.1359/jbmr.091008

20. Geoghegan, I.P., Hoey, D.A. and McNamara, L.M. (2019). Integrins in osteocyte biology and mechanotransduction. *Curr. Osteoporos. Rep.* **17**:195-206. DOI:10.1007/s11914-019-00520-2

21. Loeser, R.F. (2014). Integrins and chondrocyte-matrix interactions in articular cartilage. *Matrix Biol.* **39**:11-16. DOI:10.1016/j.matbio.2014.08.007

22. Liu, X., Yuan, Y., Wu, Y., et al. (2025). Extracellular matrix stiffness modulates myopia scleral remodeling through integrin/F-actin/YAP axis. *Invest. Ophthalmol. Vis. Sci.* **66**:22. DOI:10.1167/iovs.66.2.22

23. Murphy, R., Irnaten, M., Hopkins, A., et al. (2022). Matrix mechanotransduction via yes-associated protein in human lamina cribrosa cells in glaucoma. *Invest. Ophthalmol. Vis. Sci.* **63**:1-12. DOI:10.1167/iovs.63.1.16

24. Wu, Y., Li, L.D., Li, W., et al. (2024). Stretch-induced hepatic endothelial mechanocrine promotes hepatocyte proliferation. *Hepatology* **82**:370-387. DOI:10.1097/hep.0000000000001082

25. Lorenz, L., Axnick, J., Buschmann, T., et al. (2018). Mechanosensing by β1 integrin induces angiocrine signals for liver growth and survival. *Nature* **562**:128-132. DOI:10.1038/s41586-018-0522-3

26. Li, W., Wu, Y., Hu, W.H., et al. (2023). Direct mechanical exposure initiates hepatocyte proliferation. *JHEP Rep.* **5**:100905. DOI:10.1016/j.jhepr.2023.100905

27. Chen, H.P., Qu, J., Huang, X.W., et al. (2016). Mechanosensing by the α6-integrin confers an invasive fibroblast phenotype and mediates lung fibrosis. *Nat. Commun.* **7**:12564. DOI:10.1038/ncomms12564

28. Wu, J.N., Song, M., Li, T.Y., et al. (2015). The Rho-mDial signaling pathway is required for cyclic strain-induced cytoskeletal rearrangement of human

periodontal ligament cells. *Exp. Cell Res.* **337**:28-36. DOI:10.1016/j.yexcr.2015.07.016

29. Schröder, A., Bauer, K., Spanier, G., et al. (2018). Expression kinetics of human periodontal ligament fibroblasts in the early phases of orthodontic tooth movement. *J. Orofac. Orthop.* **79**:337-351. DOI:10.1007/s00056-018-0145-1
